# Data for identification of GPI-anchored peptides and ω-sites in cancer cell lines

**DOI:** 10.1016/j.dib.2016.04.001

**Published:** 2016-04-08

**Authors:** Yusuke Masuishi, Yayoi Kimura, Noriaki Arakawa, Hisashi Hirano

**Affiliations:** a Graduate School of Nanobioscience, Yokohama City University, Fukuura 3-9, 8 Kanazawa, Yokohama 236-0004, Japan; b Advanced Medical Research Center, Yokohama City University, Fukuura 3-9, 8 Kanazawa, Yokohama 236-0004, Japan

## Abstract

We present data obtained using a focused proteomics approach to identify the glycosylphosphatidylinositol (GPI)-anchored peptides in 19 human cancer cell lines. GPI-anchored proteins (GPI-APs), which localize to the outer leaflet of the membrane microdomains commonly referred to as lipid rafts play important roles in diverse biological processes. Due to the complex structure of the GPI-anchor moiety, it has been difficult to identify GPI-anchored peptide sequences on the proteomic scale by database searches using tools such as MASCOT. Here we provide data from 73 ω-sites derived from 49 GPI-APs in 19 human cancer cell lines. This article contains data related to the research article entitled “Identification of glycosylphosphatidylinositol-anchored proteins and ω-sites using TiO_2_-based affinity purification followed by hydrogen fluoride treatment” (Masuishi et al., 2016) [1].

**Specifications Table**

**Table:** 

Subject area	Biology
More specific subject area	Proteomics, Post-translational modification
Type of data	MS data, Table, Figure,
How data was acquired	MS data were acquired using data-dependent acquisition mode on a LTQ Orbitrap Velos mass spectrometer (Thermo Fisher Scientific).
Data format	Raw, Analyzed
Experimental factors	Digested GPI-anchored peptides were treated with hydrogen fluoride.
Experimental features	GPI-anchored proteins were isolated from human cancer cell lines using Triton X-114 phase separation and PI-PLC treatment. The digested peptides were analyzed by nano-LC-MS/MS.
Data source location	*Fukuura 3-9, 8 Kanazawa, Yokohama 236-0004, Japan*
Data accessibility	Data is within this article and have been deposited in the ProteomeXchange Consortium (http://proteomecentral.proteomexchange.org) via the PRIDE partner repository with the dataset identifier PRIDE: PXD003105.

**Value of the data**

• Identification of novel GPI-APs and ω-sites.
• These data provide the first evidence that the GPI-anchor attaches to multiple amino acids in the C-terminal site, yielding a variety of protein species.
• These data may be helpful in understanding the mechanisms of GPI anchoring.

## 1 Data

We present 46 RAW mass spectrometry data files with negative controls that correspond to our LC-MS/MS analysis of 19 human cancer cell lines (ovarian cancer cell lines OVISE, A2780, OVCAR-3, OVMANA, OVSAHO, and OVSAYO, renal cancer cell lines 786-O, A498, ACHN, Caki-1, Caki-2 and UMRC3, bladder cancer cell lines 5637, T24 and UMUC3, prostate cancer cell lines DU145, LNCaP and PC3, and neuroblastoma cell line SH-SY5Y).

The mass spectrometry proteomics data have been deposited to the ProteomeXchange Consortium via the PRIDE partner repository [2] with the dataset identifier PRIDE: PXD003105.

## 2 Experimental design, materials and methods

The detailed method is described elsewhere [1].

### 2.1 Isolation of GPI-Aps

This procedure was performed as previously described [3]. The GPI-AP–enriched fraction, termed the detergent-resistant membrane (DRM) fraction, was purified by sucrose gradient fractionation. The DRM fraction was resuspended in 50 mM HEPES [pH 7.5] and 1% (v/v) Triton X-114. This solution was subjected to Triton X-114 two-phase separation with or without Phosphatidylinositol-specific phospholipase C (PI-PLC) (from *Bacillus cereus*; Molecular-Probes USA) treatment. Supernatants were concentrated by trichloroacetic acid (TCA)/acetone precipitation and subjected to in-solution digestion (digestion with either trypsin, chymotrypsin).

### 2.2 GPI-APs digestion

For in-solution digestion, PI-PLC treated GPI-APs were resuspended in 20 µl of 8 M urea, and dithiothreitol (DTT) was added to a final concentration of 10 mM. This mixture was incubated for 30 min at 37 °C, chilled, brought to a final concentration of 10 mM iodoacetic acid for S-alkylation, and incubated in the dark at room temperature for 15 min. After the alkylation, protein mixture was diluted with 3 volumes of 50 mM NH_4_HCO_3_ at a final concentration of 2 M urea. Proteins were digested with 0.1 µg trypsin (Trypsin Gold, MS Grade; Promega, USA) or 0.1 µg chymotrypsin (Chymotrypsin Sequencing Grade; Roche Applied Science, USA), and the sample was incubated at 37 °C for 18 h. To stop the digestion, 1 µl of 20% trifluoroacetic acid (TFA) (Wako, Japan) was added to the sample. The peptide fragments were desalted using StageTips [4] with C18 Empore disk membranes (3M Company, USA) and SDB (3M Company), and then eluted with 200 µl of 60% (v/v) acetonitrile/0.1% (v/v) TFA. The eluates were immediately dried under vacuum and dissolved in 0.1% TFA.

### 2.3 GPI-anchored peptide enrichment and aqueous hydrogen fluoride (HF) treatment

The GPI-anchored peptide enrichment procedure was carried out using the Titansphere Phos-TiO Kit (GL Sciences, Japan) according to the manufacturer׳s instructions. Bound GPI-anchored peptides were eluted with 50 µl of 5% NH_3_–H_2_O. Eluates were immediately dried under vacuum, and then treated with or without 20 µl of 50% (v/v) aqueous hydrogen fluoride (HF) (Wako, Japan) for 12 h at 4 °C to cleave the ethanolamine–phosphate bond. The peptide samples were again completely dried under vacuum, and then dissolved in 0.1% TFA for LC–MS/MS analysis. The negative control PI-PLC(-) or HF(-) samples from OVISE cell and SH-SY5Y cell are shown in Table S1.

### 2.4 Nano-LC and LTQ orbitrap velos setup

Prior to injection into the mass spectrometer, The peptide samples were loaded in a reverse-phase precolumn (C18 PepMap column, LC Packings, USA) and resolved on a nanoscale C18 PepMap capillary column (75 µm id×15 cm, LC Packings, USA) at a flow rate of 300 nl/min with a gradient of acetonitrile/0.1% (v/v) formic acid. The mobile phases were solvent A (0.1% formic acid/2% (v/v) acetonitrile) and B (95% acetonitrile, 5% water, 0.1% formic acid). Peptides were separated using a linear gradient from 5% to 40% solvent B for 30 min. The full-scan mass spectra were measured from *m/z* 350–1200 in positive-ion electrospray ionization mode on a LTQ Orbitrap Velos mass spectrometer (Thermo Fisher Scientific, USA) operated in data-dependent mode and subjected to CID fragmentation using the TOP15 strategy. In brief, a scan cycle was initiated with a full scan of high mass accuracy in the Orbitrap, followed by MS/MS scans of the fifteen most intense precursor ions in the linear ion trap, with dynamic exclusion of previously selected ions. The other parameter settings were as follows: normalized collision energy, 35%; electrospray voltage, 1.8 kV; capillary temperature, 250 °C, and isolation width, 2 (*m/z*).

### 2.5 Data analysis

The raw MS spectrum was processed using Proteome Discoverer (v.1.3.0.339, Thermo Fisher Scientific), applying Mascot (v.2.4.0, Matrix Science) for peptide identification. The data were queried against UniProt/SWISS-PROT database (v2012-0711; *Homo sapiens* 20,232 sequences). All database searches were performed using a precursor mass tolerance of ±5 ppm, fragment ion mass tolerance of ±0.5 Da, enzyme name set to semi-specific trypsin or semi-specific chymotrypsin, and a maximum missed-cleavages value of 2. Oxidation of Met were set as variable modifications. Fixed modifications were set to carboxymethyl of Cys. To identify GPI-anchored peptide sequences, the EtN moiety (+43.0422 Da) was set as a variable modifications of the C-terminal peptide. As a control, carbamylation of Lys, Arg, Cys., and the N-terminus were used as variable modifications (Table S2).GPI-anchored peptide identification was considered positive when a match yielded one peptide with an ion score greater than 30 and the false-discovery rate was fixed to a threshold of 0.01 at peptide level. Table S1 provides a detailed list of all identified GPI-anchored peptides from 19 human cancer cell lines along with the following additional information: accession number, gene name, enzyme used, identified peptide sequence, ion score, and molecular mass. The MS/MS spectra derived from 73 GPI-anchored peptides (EtN modified peptides) are shown in Figure S1. A detailed list of all GPI-anchored peptides identified using an FDR of 0.05 is provided in Table S3. The mass spectrometry proteomics data have been deposited to the ProteomeXchange Consortium (http://proteomecentral.proteomexchange.org) via the PRIDE partner repository [2] with the dataset identifier PRIDE: PXD003105.
