# Supplementary material for: Data for identification of GPI-anchored peptides and ω-sites in cancer cell lines
Source: Data Brief. 2016 Apr 8;7:1302–5. doi: 10.1016/j.dib.2016.04.001 (PMC4838930; doi:10.1016/j.dib.2016.04.001)
Supplement: Supplementary file 2 — Supplementary material [file mmc2.pptx]

## Slide 1
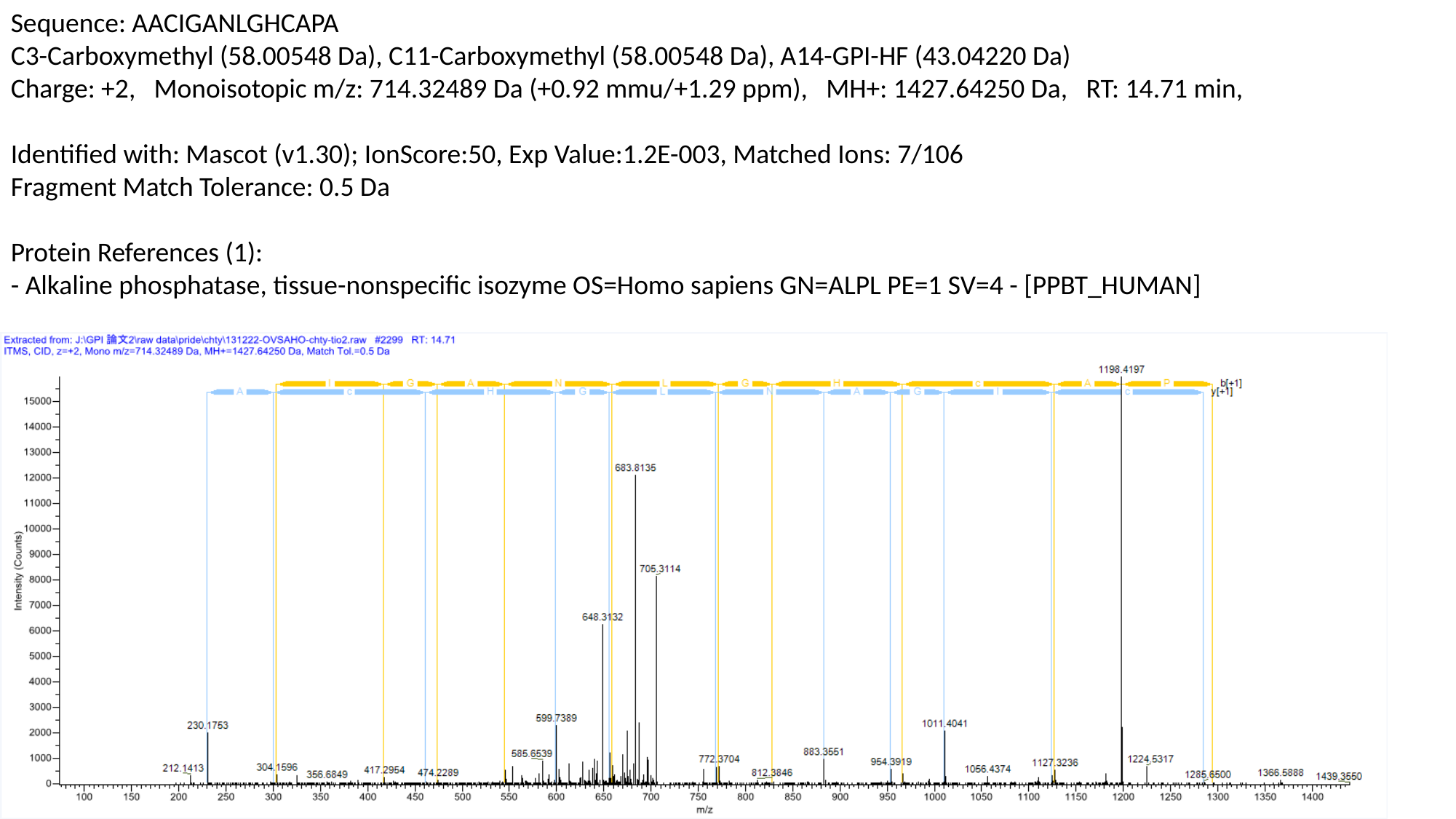

Sequence: AACIGANLGHCAPA
C3-Carboxymethyl (58.00548 Da), C11-Carboxymethyl (58.00548 Da), A14-GPI-HF (43.04220 Da)
Charge: +2, Monoisotopic m/z: 714.32489 Da (+0.92 mmu/+1.29 ppm), MH+: 1427.64250 Da, RT: 14.71 min,
Identified with: Mascot (v1.30); IonScore:50, Exp Value:1.2E-003, Matched Ions: 7/106
Fragment Match Tolerance: 0.5 Da
Protein References (1):
- Alkaline phosphatase, tissue-nonspecific isozyme OS=Homo sapiens GN=ALPL PE=1 SV=4 - [PPBT_HUMAN]

## Slide 2
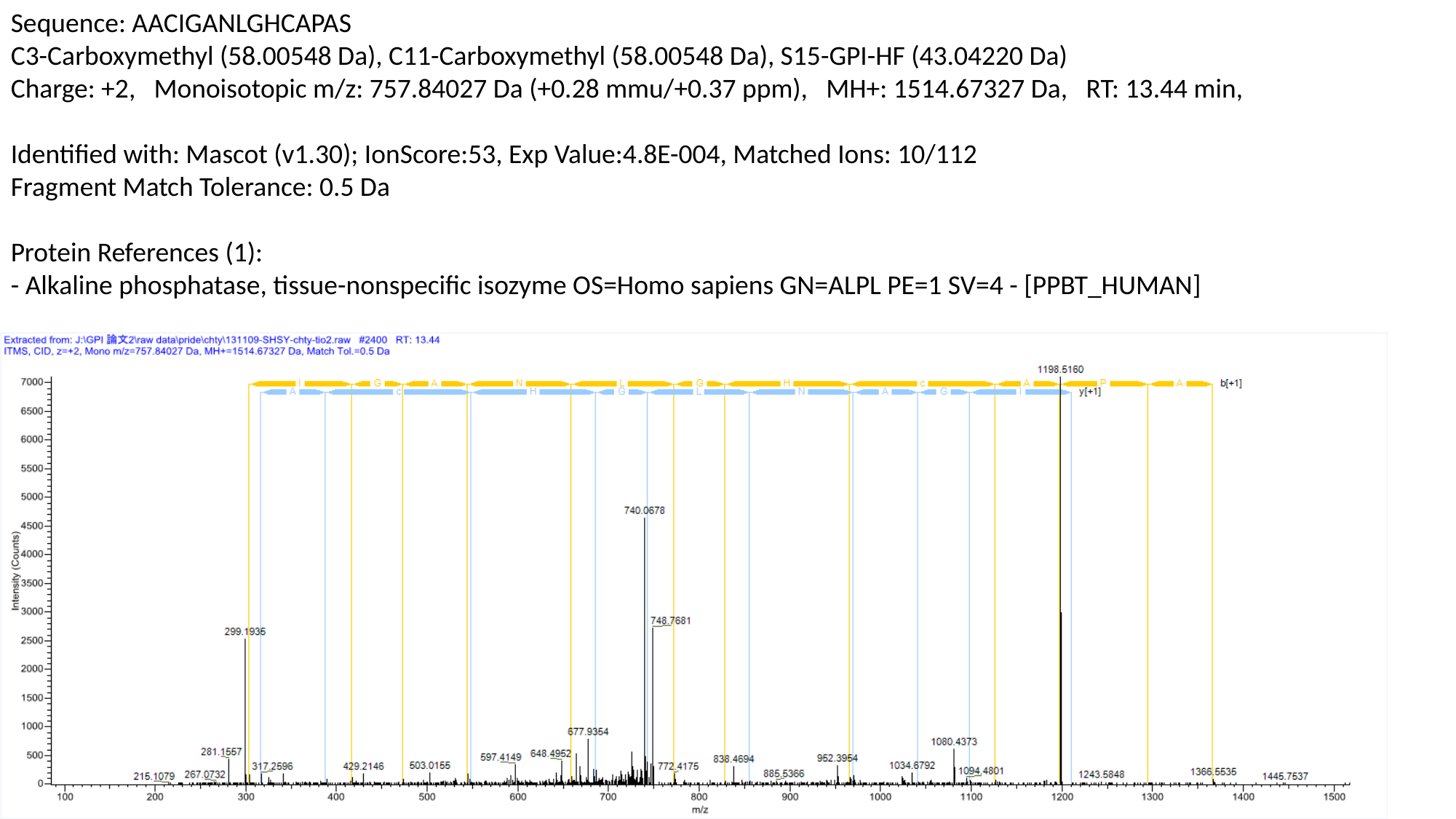

Sequence: AACIGANLGHCAPAS
C3-Carboxymethyl (58.00548 Da), C11-Carboxymethyl (58.00548 Da), S15-GPI-HF (43.04220 Da)
Charge: +2, Monoisotopic m/z: 757.84027 Da (+0.28 mmu/+0.37 ppm), MH+: 1514.67327 Da, RT: 13.44 min,
Identified with: Mascot (v1.30); IonScore:53, Exp Value:4.8E-004, Matched Ions: 10/112
Fragment Match Tolerance: 0.5 Da
Protein References (1):
- Alkaline phosphatase, tissue-nonspecific isozyme OS=Homo sapiens GN=ALPL PE=1 SV=4 - [PPBT_HUMAN]

## Slide 3
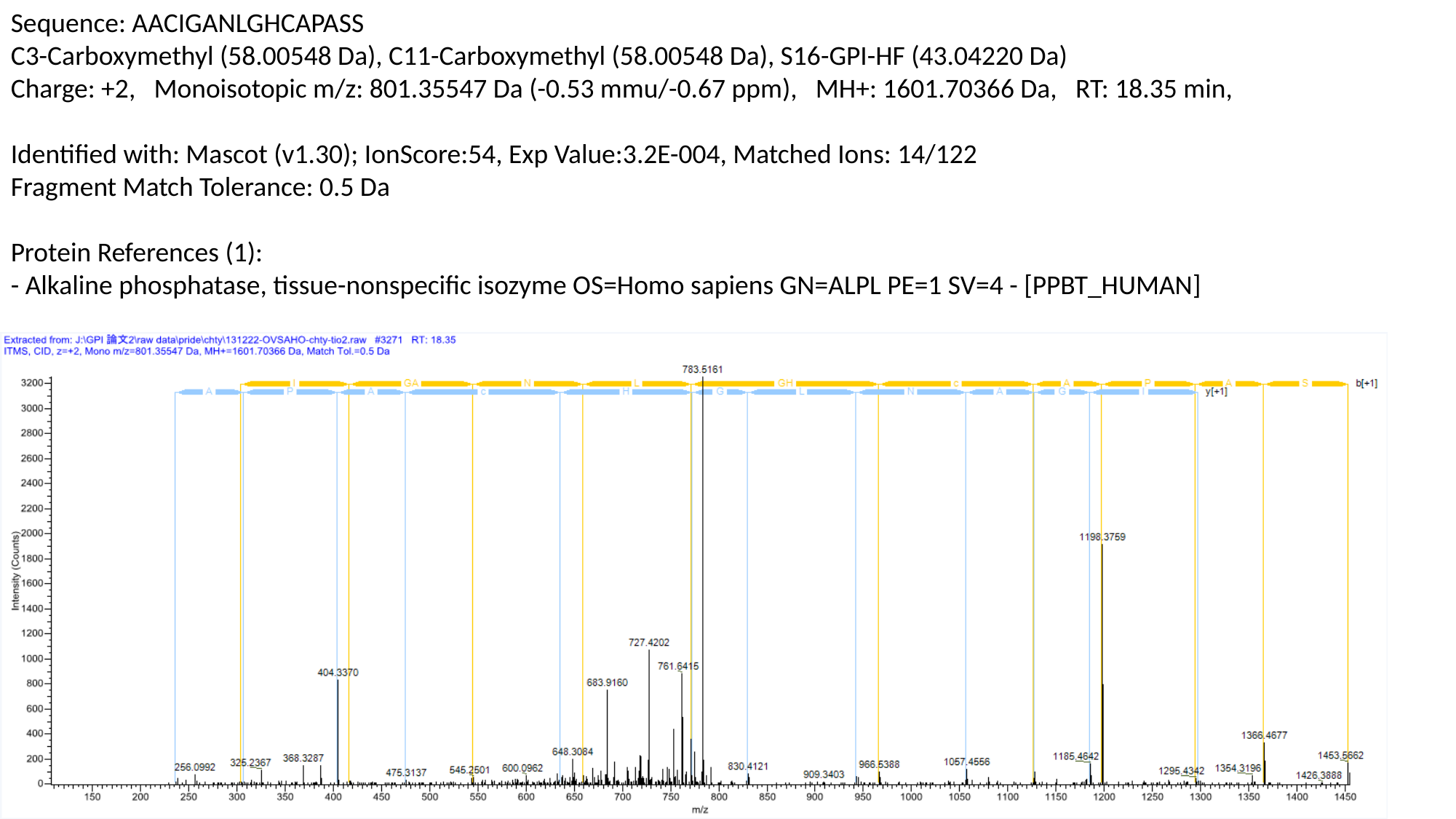

Sequence: AACIGANLGHCAPASS
C3-Carboxymethyl (58.00548 Da), C11-Carboxymethyl (58.00548 Da), S16-GPI-HF (43.04220 Da)
Charge: +2, Monoisotopic m/z: 801.35547 Da (-0.53 mmu/-0.67 ppm), MH+: 1601.70366 Da, RT: 18.35 min,
Identified with: Mascot (v1.30); IonScore:54, Exp Value:3.2E-004, Matched Ions: 14/122
Fragment Match Tolerance: 0.5 Da
Protein References (1):
- Alkaline phosphatase, tissue-nonspecific isozyme OS=Homo sapiens GN=ALPL PE=1 SV=4 - [PPBT_HUMAN]

## Slide 4
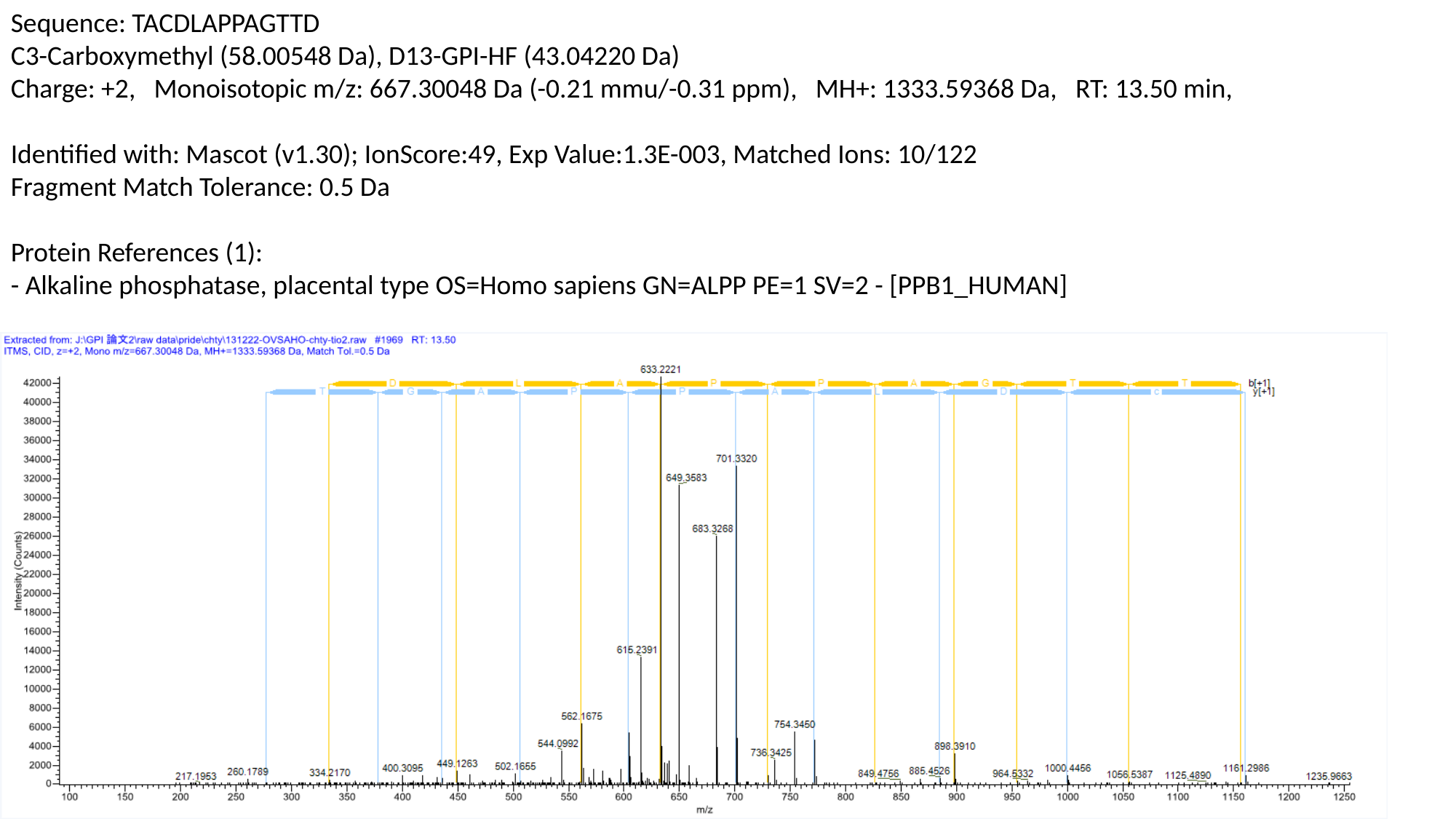

Sequence: TACDLAPPAGTTD
C3-Carboxymethyl (58.00548 Da), D13-GPI-HF (43.04220 Da)
Charge: +2, Monoisotopic m/z: 667.30048 Da (-0.21 mmu/-0.31 ppm), MH+: 1333.59368 Da, RT: 13.50 min,
Identified with: Mascot (v1.30); IonScore:49, Exp Value:1.3E-003, Matched Ions: 10/122
Fragment Match Tolerance: 0.5 Da
Protein References (1):
- Alkaline phosphatase, placental type OS=Homo sapiens GN=ALPP PE=1 SV=2 - [PPB1_HUMAN]

## Slide 5
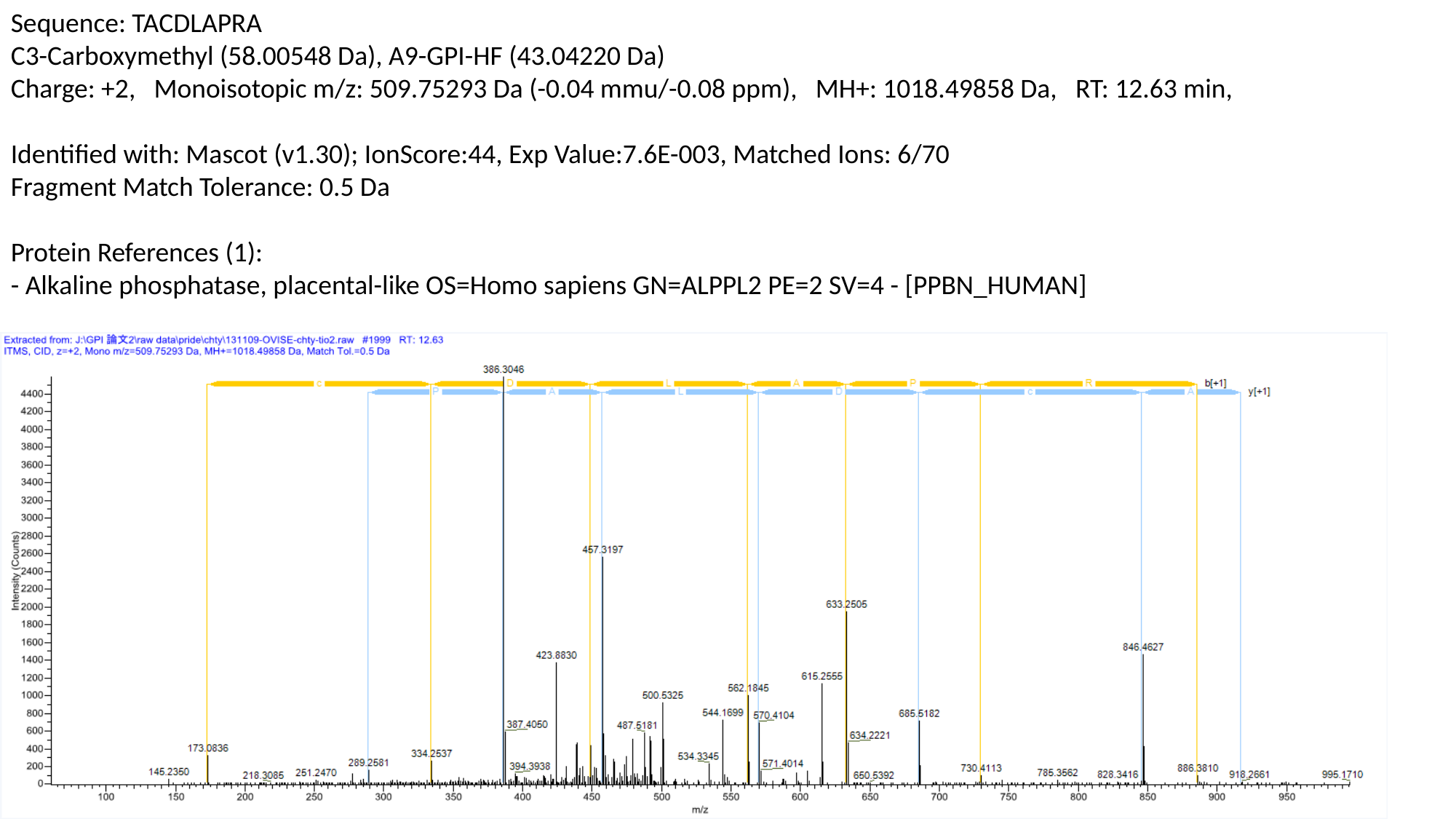

Sequence: TACDLAPRA
C3-Carboxymethyl (58.00548 Da), A9-GPI-HF (43.04220 Da)
Charge: +2, Monoisotopic m/z: 509.75293 Da (-0.04 mmu/-0.08 ppm), MH+: 1018.49858 Da, RT: 12.63 min,
Identified with: Mascot (v1.30); IonScore:44, Exp Value:7.6E-003, Matched Ions: 6/70
Fragment Match Tolerance: 0.5 Da
Protein References (1):
- Alkaline phosphatase, placental-like OS=Homo sapiens GN=ALPPL2 PE=2 SV=4 - [PPBN_HUMAN]

## Slide 6
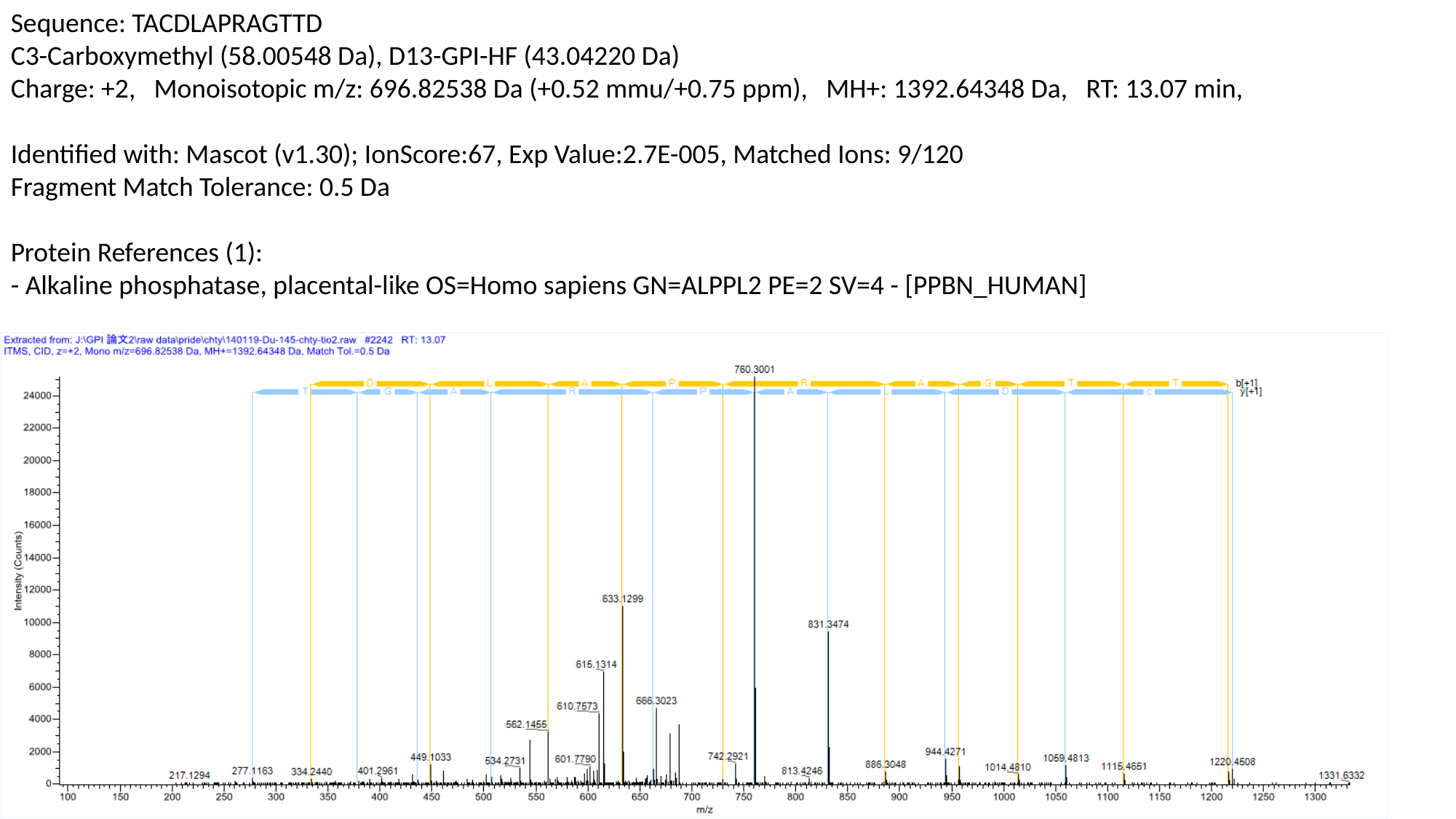

Sequence: TACDLAPRAGTTD
C3-Carboxymethyl (58.00548 Da), D13-GPI-HF (43.04220 Da)
Charge: +2, Monoisotopic m/z: 696.82538 Da (+0.52 mmu/+0.75 ppm), MH+: 1392.64348 Da, RT: 13.07 min,
Identified with: Mascot (v1.30); IonScore:67, Exp Value:2.7E-005, Matched Ions: 9/120
Fragment Match Tolerance: 0.5 Da
Protein References (1):
- Alkaline phosphatase, placental-like OS=Homo sapiens GN=ALPPL2 PE=2 SV=4 - [PPBN_HUMAN]

## Slide 7
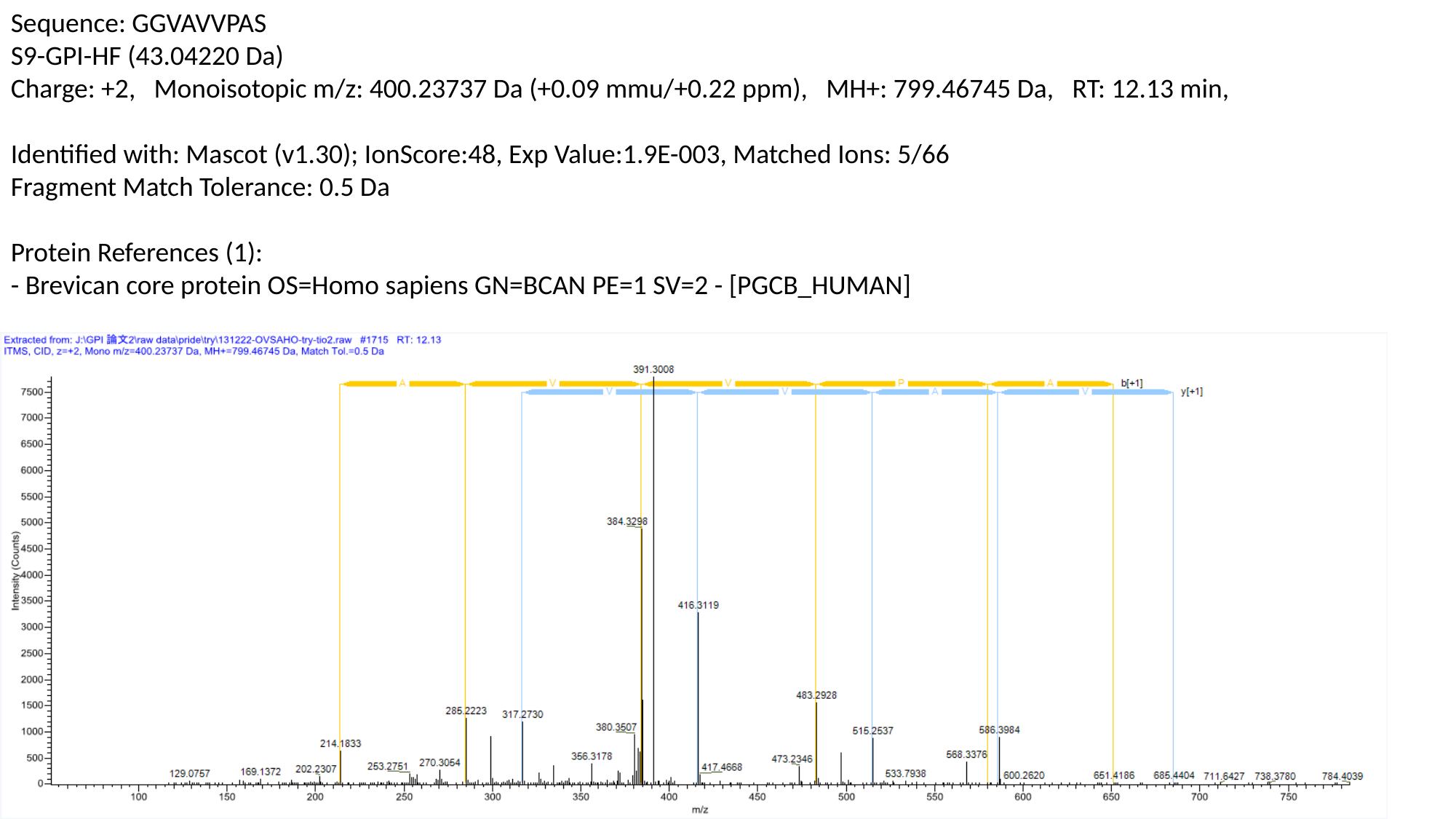

Sequence: GGVAVVPAS
S9-GPI-HF (43.04220 Da)
Charge: +2, Monoisotopic m/z: 400.23737 Da (+0.09 mmu/+0.22 ppm), MH+: 799.46745 Da, RT: 12.13 min,
Identified with: Mascot (v1.30); IonScore:48, Exp Value:1.9E-003, Matched Ions: 5/66
Fragment Match Tolerance: 0.5 Da
Protein References (1):
- Brevican core protein OS=Homo sapiens GN=BCAN PE=1 SV=2 - [PGCB_HUMAN]

## Slide 8
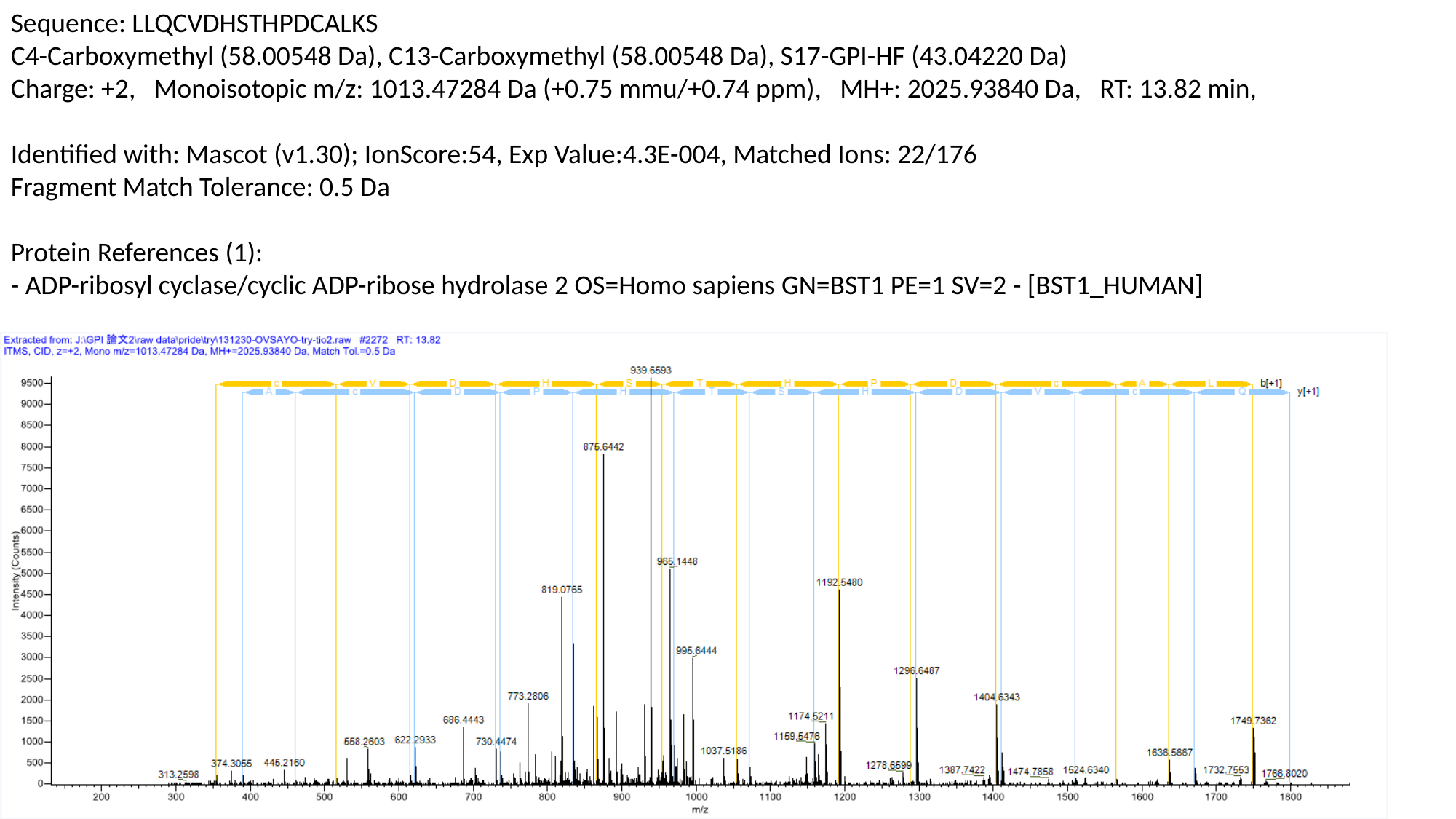

Sequence: LLQCVDHSTHPDCALKS
C4-Carboxymethyl (58.00548 Da), C13-Carboxymethyl (58.00548 Da), S17-GPI-HF (43.04220 Da)
Charge: +2, Monoisotopic m/z: 1013.47284 Da (+0.75 mmu/+0.74 ppm), MH+: 2025.93840 Da, RT: 13.82 min,
Identified with: Mascot (v1.30); IonScore:54, Exp Value:4.3E-004, Matched Ions: 22/176
Fragment Match Tolerance: 0.5 Da
Protein References (1):
- ADP-ribosyl cyclase/cyclic ADP-ribose hydrolase 2 OS=Homo sapiens GN=BST1 PE=1 SV=2 - [BST1_HUMAN]

## Slide 9
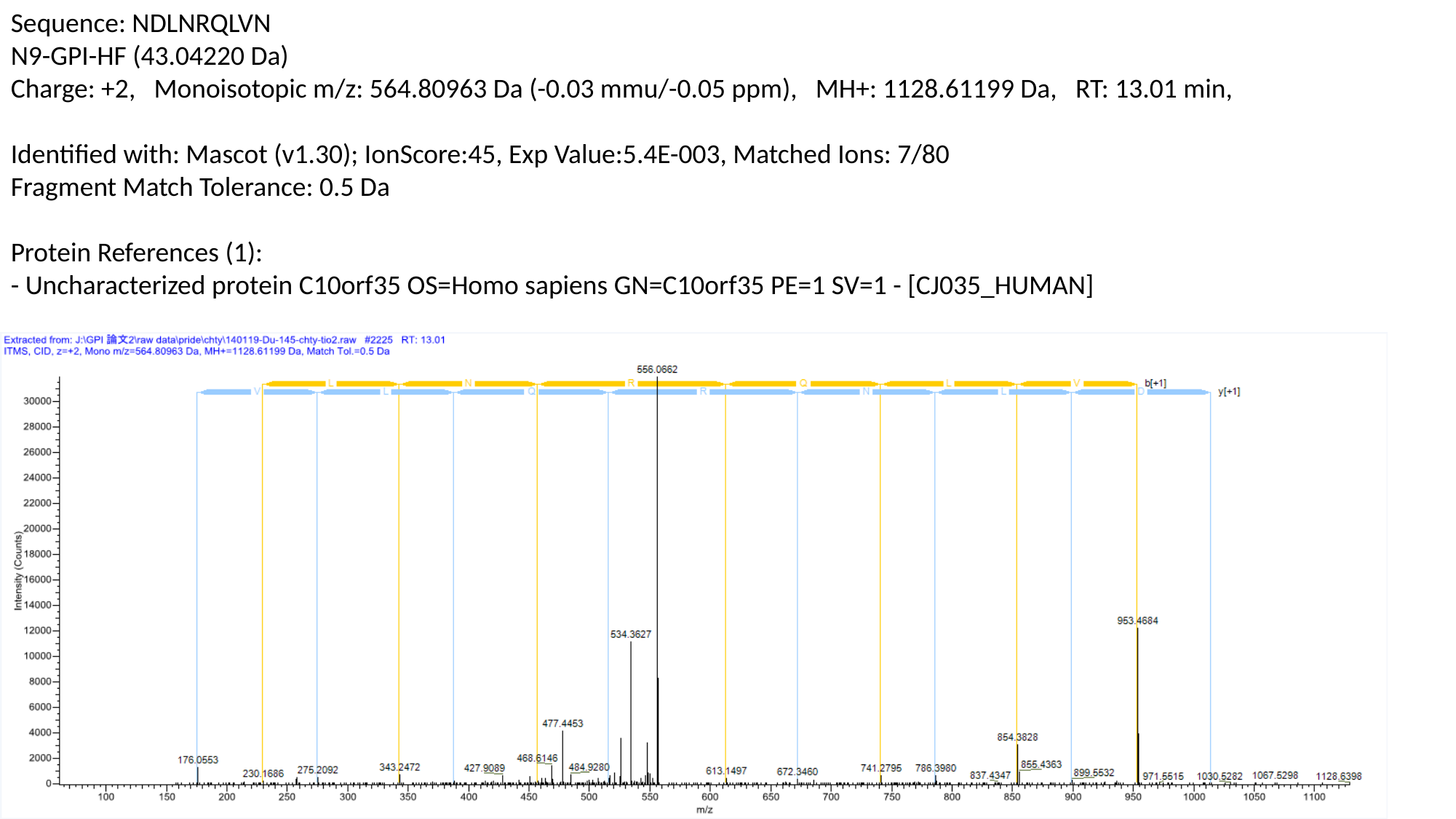

Sequence: NDLNRQLVN
N9-GPI-HF (43.04220 Da)
Charge: +2, Monoisotopic m/z: 564.80963 Da (-0.03 mmu/-0.05 ppm), MH+: 1128.61199 Da, RT: 13.01 min,
Identified with: Mascot (v1.30); IonScore:45, Exp Value:5.4E-003, Matched Ions: 7/80
Fragment Match Tolerance: 0.5 Da
Protein References (1):
- Uncharacterized protein C10orf35 OS=Homo sapiens GN=C10orf35 PE=1 SV=1 - [CJ035_HUMAN]

## Slide 10
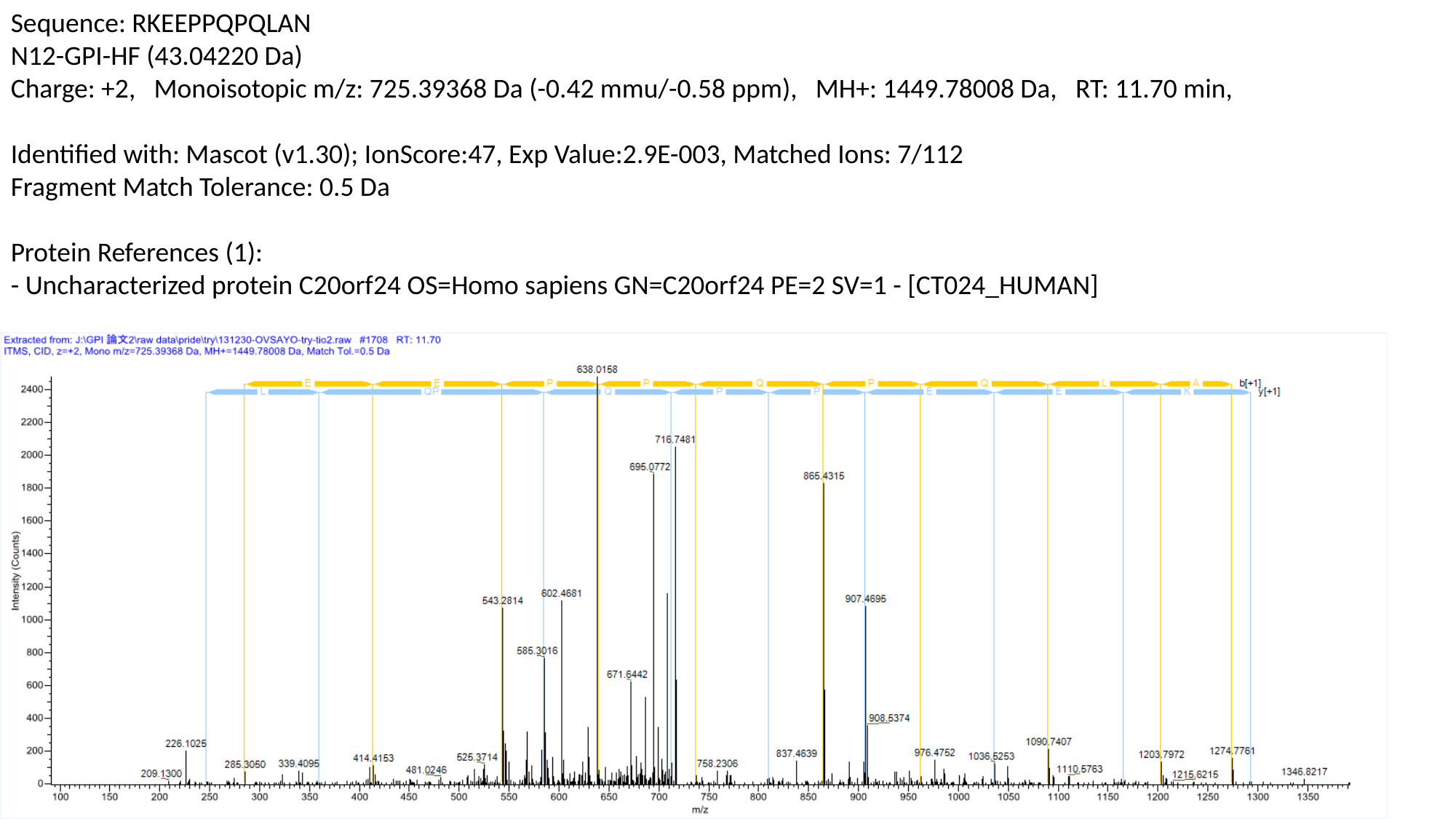

Sequence: RKEEPPQPQLAN
N12-GPI-HF (43.04220 Da)
Charge: +2, Monoisotopic m/z: 725.39368 Da (-0.42 mmu/-0.58 ppm), MH+: 1449.78008 Da, RT: 11.70 min,
Identified with: Mascot (v1.30); IonScore:47, Exp Value:2.9E-003, Matched Ions: 7/112
Fragment Match Tolerance: 0.5 Da
Protein References (1):
- Uncharacterized protein C20orf24 OS=Homo sapiens GN=C20orf24 PE=2 SV=1 - [CT024_HUMAN]

## Slide 11
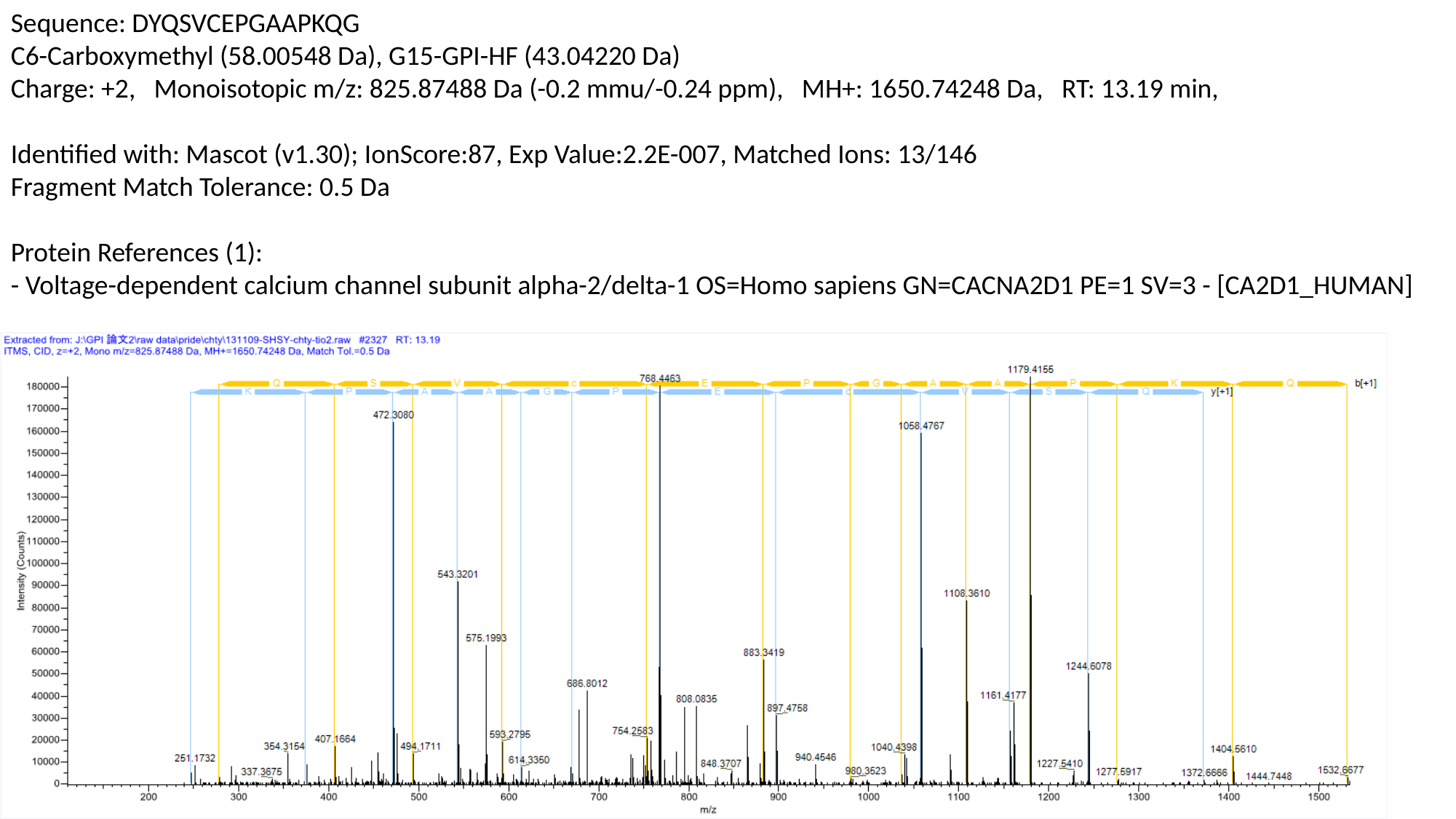

Sequence: DYQSVCEPGAAPKQG
C6-Carboxymethyl (58.00548 Da), G15-GPI-HF (43.04220 Da)
Charge: +2, Monoisotopic m/z: 825.87488 Da (-0.2 mmu/-0.24 ppm), MH+: 1650.74248 Da, RT: 13.19 min,
Identified with: Mascot (v1.30); IonScore:87, Exp Value:2.2E-007, Matched Ions: 13/146
Fragment Match Tolerance: 0.5 Da
Protein References (1):
- Voltage-dependent calcium channel subunit alpha-2/delta-1 OS=Homo sapiens GN=CACNA2D1 PE=1 SV=3 - [CA2D1_HUMAN]

## Slide 12
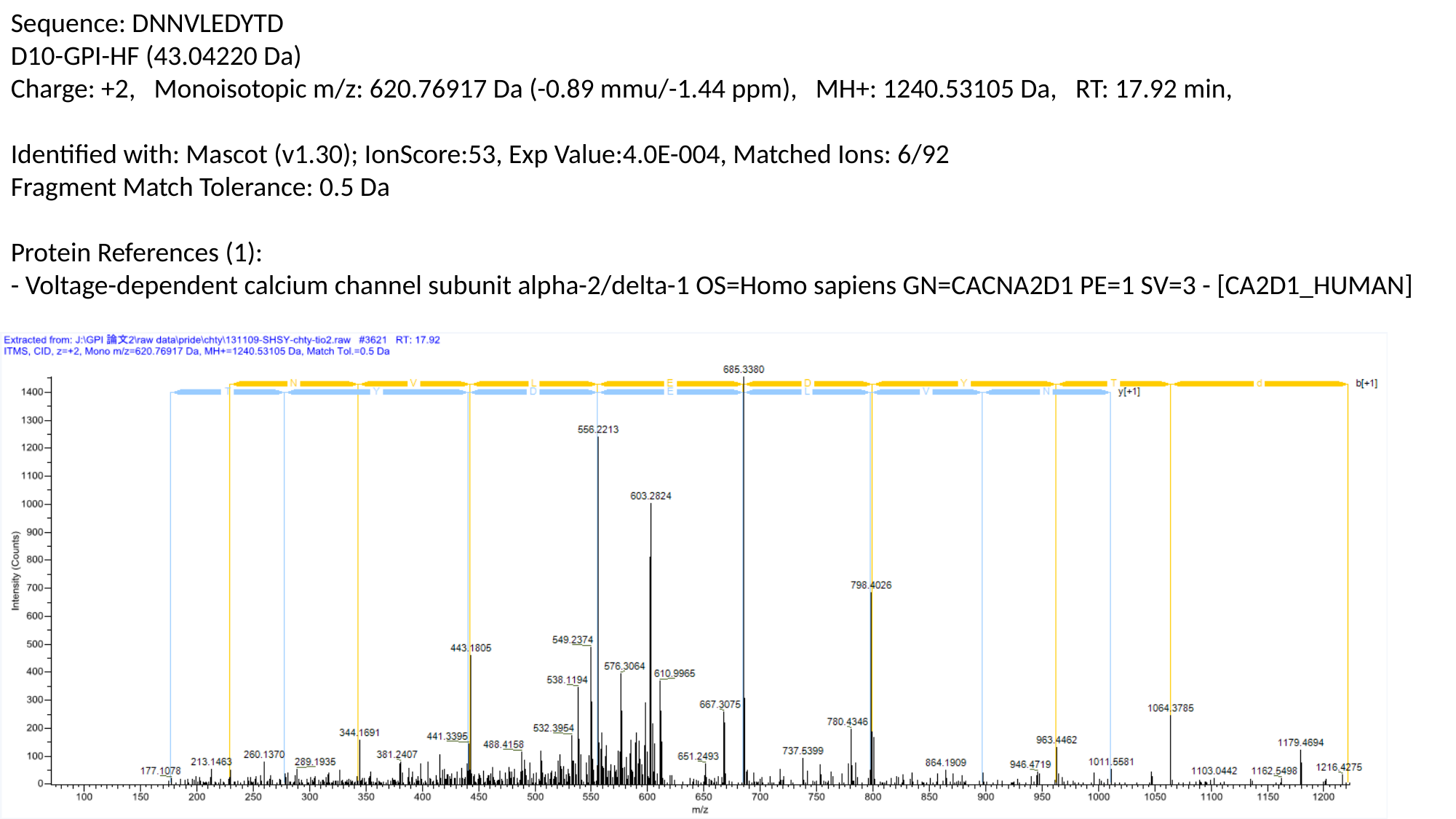

Sequence: DNNVLEDYTD
D10-GPI-HF (43.04220 Da)
Charge: +2, Monoisotopic m/z: 620.76917 Da (-0.89 mmu/-1.44 ppm), MH+: 1240.53105 Da, RT: 17.92 min,
Identified with: Mascot (v1.30); IonScore:53, Exp Value:4.0E-004, Matched Ions: 6/92
Fragment Match Tolerance: 0.5 Da
Protein References (1):
- Voltage-dependent calcium channel subunit alpha-2/delta-1 OS=Homo sapiens GN=CACNA2D1 PE=1 SV=3 - [CA2D1_HUMAN]

## Slide 13
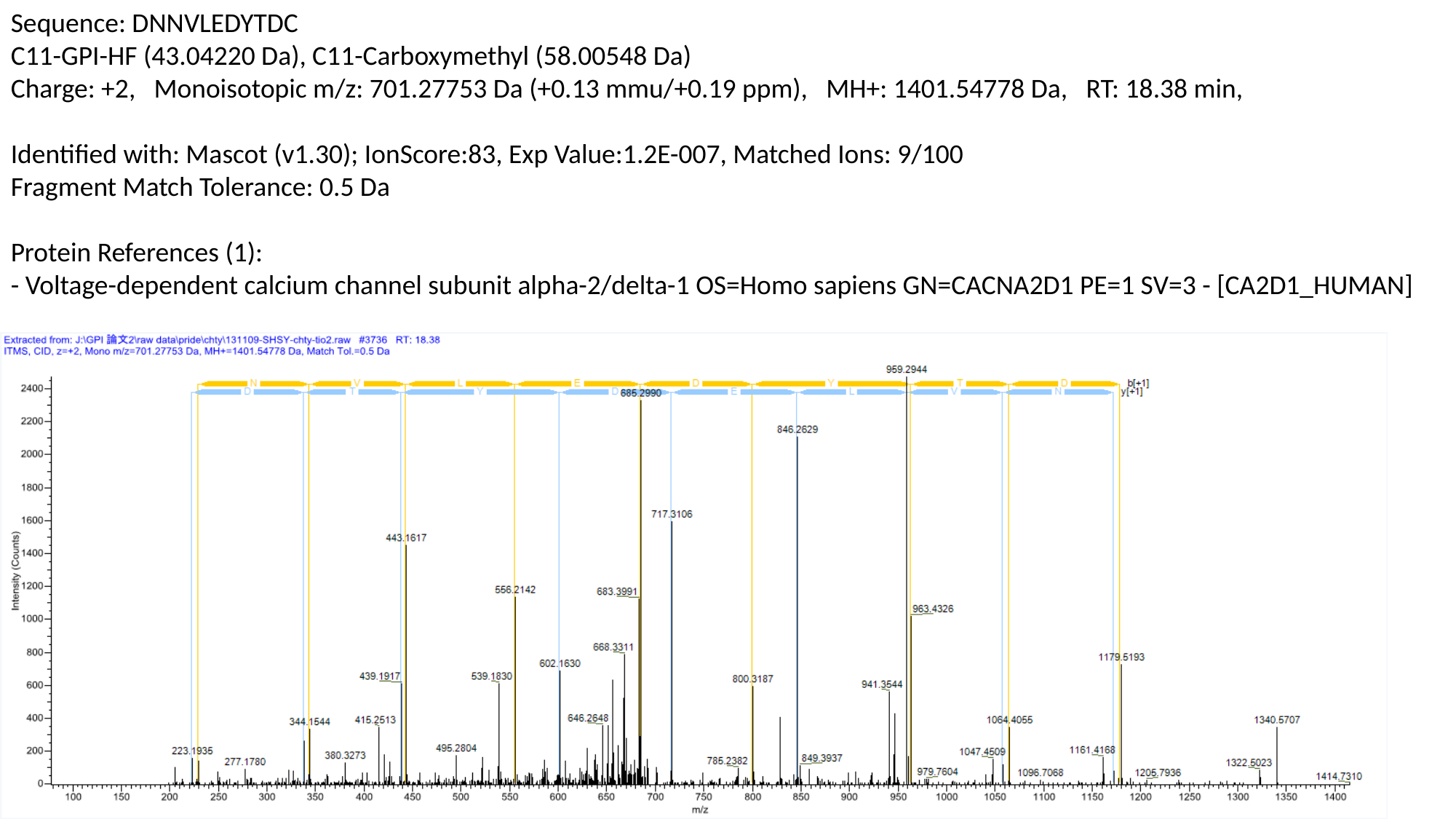

Sequence: DNNVLEDYTDC
C11-GPI-HF (43.04220 Da), C11-Carboxymethyl (58.00548 Da)
Charge: +2, Monoisotopic m/z: 701.27753 Da (+0.13 mmu/+0.19 ppm), MH+: 1401.54778 Da, RT: 18.38 min,
Identified with: Mascot (v1.30); IonScore:83, Exp Value:1.2E-007, Matched Ions: 9/100
Fragment Match Tolerance: 0.5 Da
Protein References (1):
- Voltage-dependent calcium channel subunit alpha-2/delta-1 OS=Homo sapiens GN=CACNA2D1 PE=1 SV=3 - [CA2D1_HUMAN]

## Slide 14
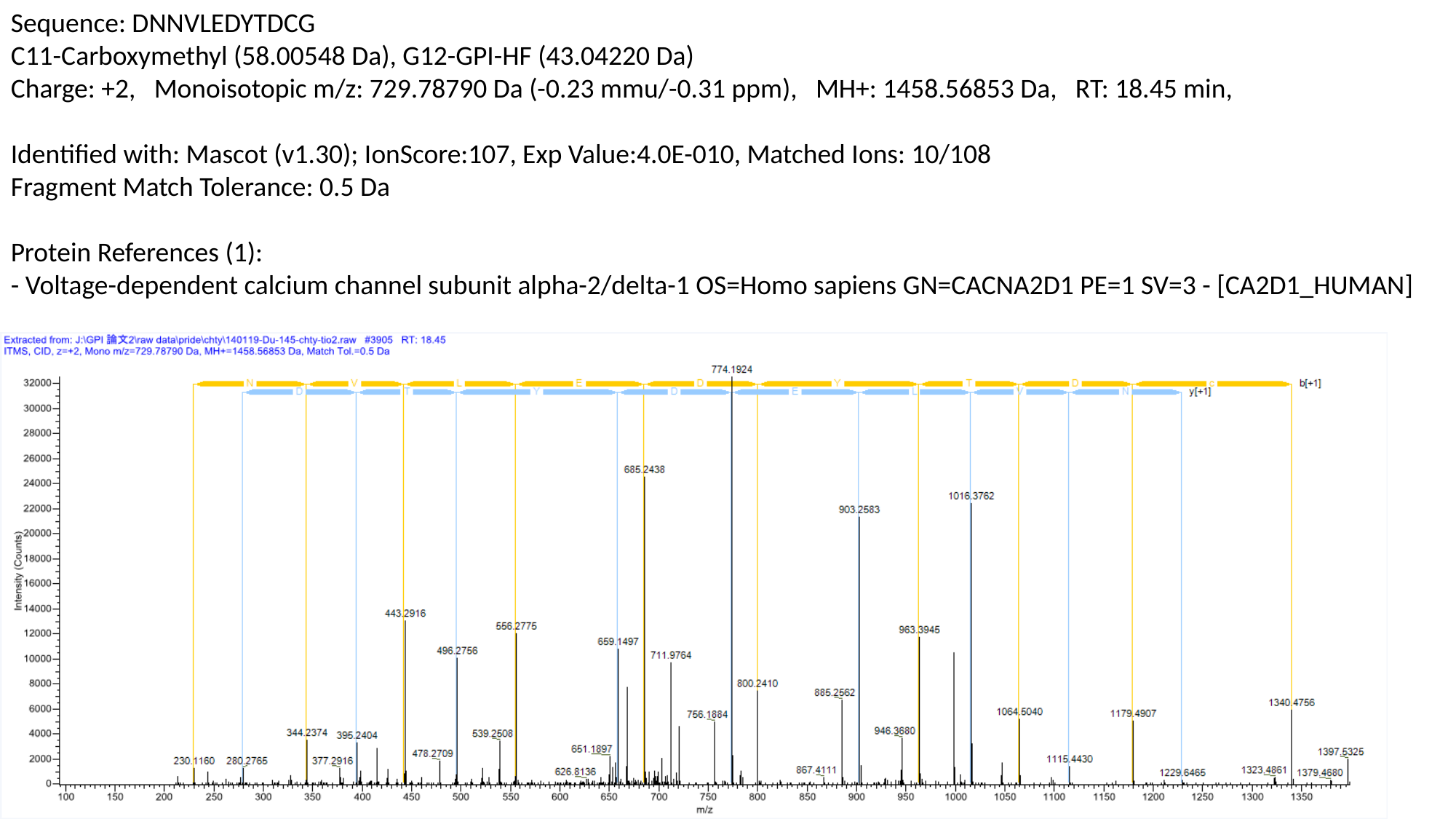

Sequence: DNNVLEDYTDCG
C11-Carboxymethyl (58.00548 Da), G12-GPI-HF (43.04220 Da)
Charge: +2, Monoisotopic m/z: 729.78790 Da (-0.23 mmu/-0.31 ppm), MH+: 1458.56853 Da, RT: 18.45 min,
Identified with: Mascot (v1.30); IonScore:107, Exp Value:4.0E-010, Matched Ions: 10/108
Fragment Match Tolerance: 0.5 Da
Protein References (1):
- Voltage-dependent calcium channel subunit alpha-2/delta-1 OS=Homo sapiens GN=CACNA2D1 PE=1 SV=3 - [CA2D1_HUMAN]

## Slide 15
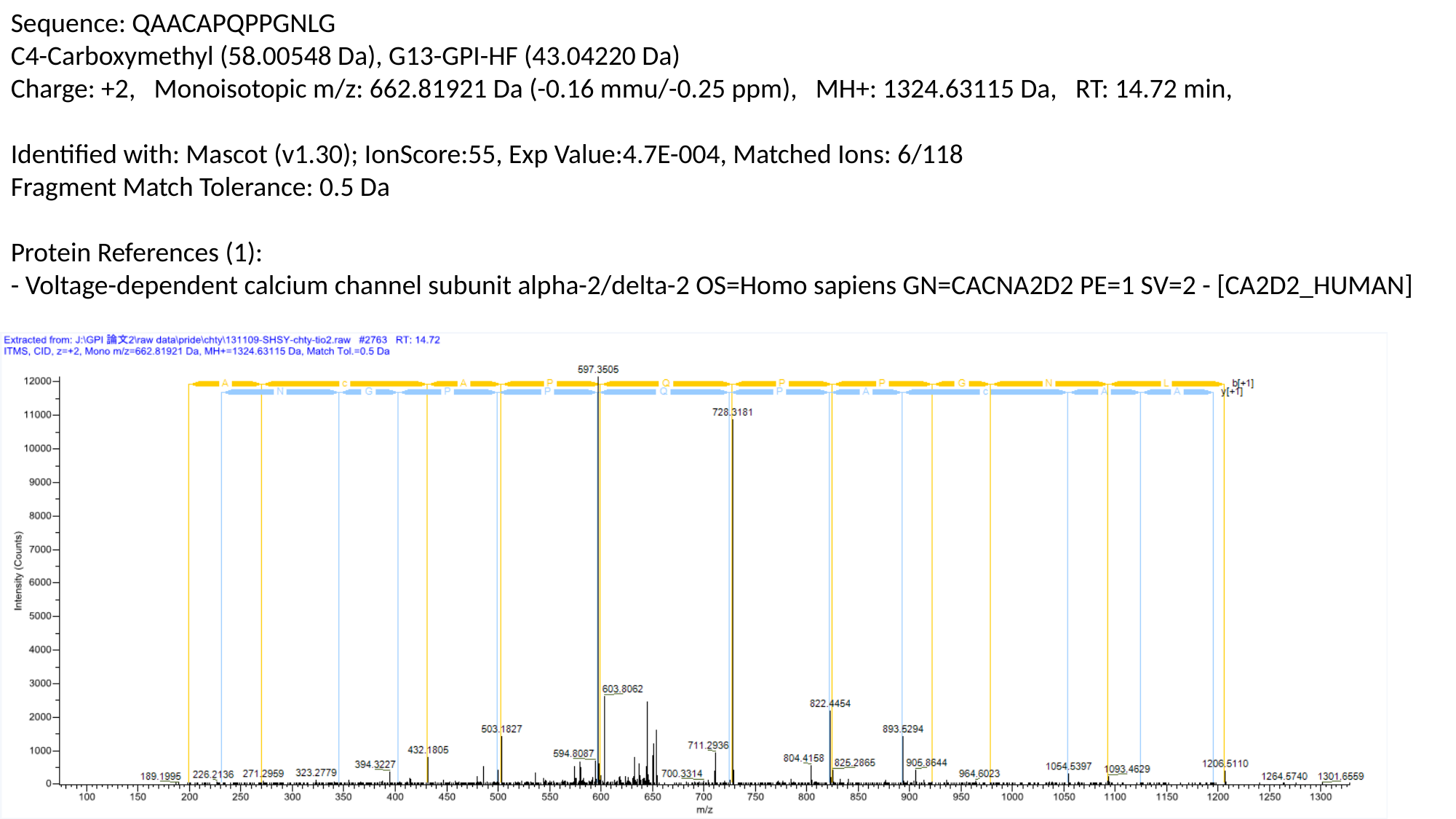

Sequence: QAACAPQPPGNLG
C4-Carboxymethyl (58.00548 Da), G13-GPI-HF (43.04220 Da)
Charge: +2, Monoisotopic m/z: 662.81921 Da (-0.16 mmu/-0.25 ppm), MH+: 1324.63115 Da, RT: 14.72 min,
Identified with: Mascot (v1.30); IonScore:55, Exp Value:4.7E-004, Matched Ions: 6/118
Fragment Match Tolerance: 0.5 Da
Protein References (1):
- Voltage-dependent calcium channel subunit alpha-2/delta-2 OS=Homo sapiens GN=CACNA2D2 PE=1 SV=2 - [CA2D2_HUMAN]

## Slide 16
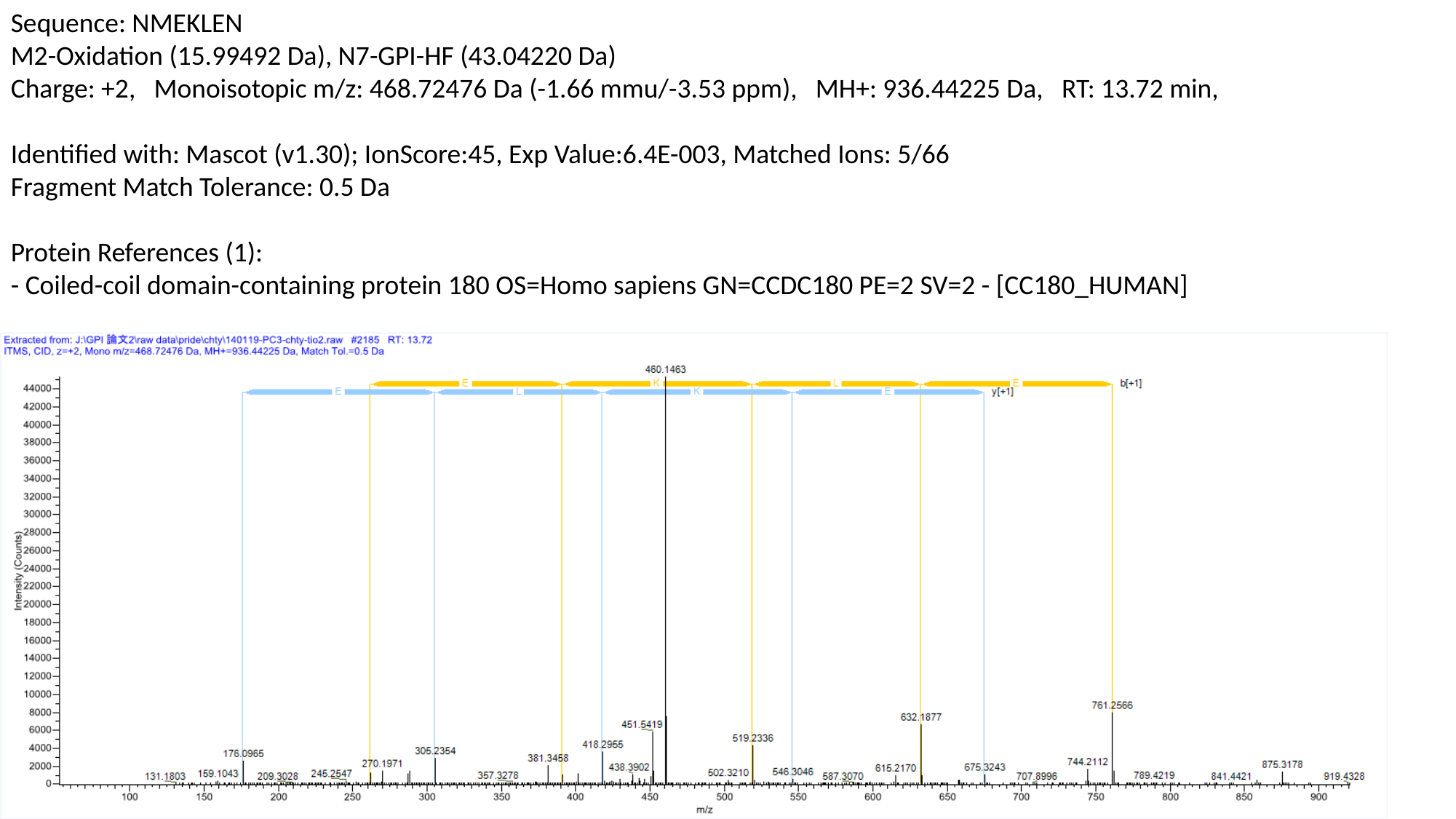

Sequence: NMEKLEN
M2-Oxidation (15.99492 Da), N7-GPI-HF (43.04220 Da)
Charge: +2, Monoisotopic m/z: 468.72476 Da (-1.66 mmu/-3.53 ppm), MH+: 936.44225 Da, RT: 13.72 min,
Identified with: Mascot (v1.30); IonScore:45, Exp Value:6.4E-003, Matched Ions: 5/66
Fragment Match Tolerance: 0.5 Da
Protein References (1):
- Coiled-coil domain-containing protein 180 OS=Homo sapiens GN=CCDC180 PE=2 SV=2 - [CC180_HUMAN]

## Slide 17
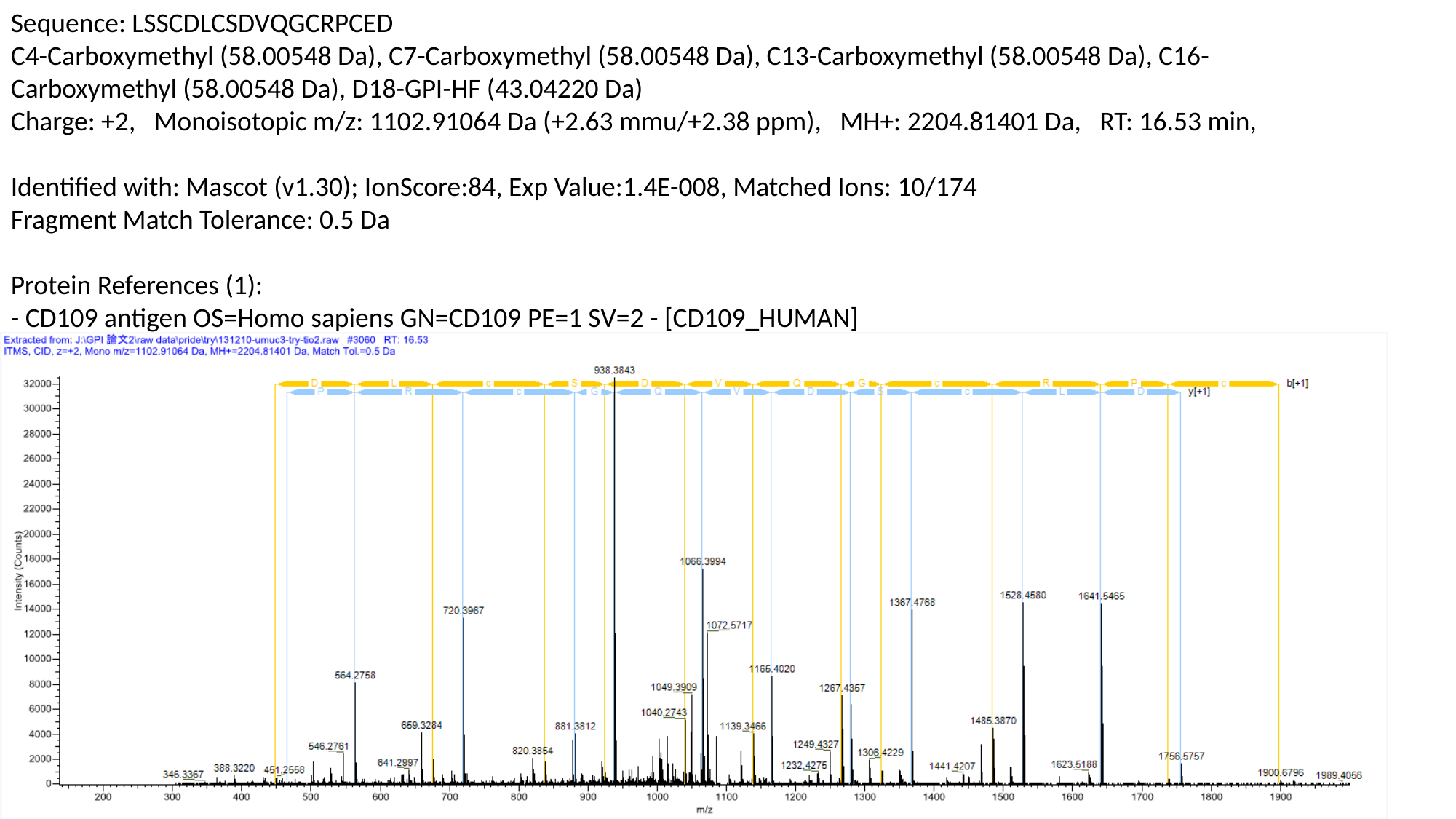

Sequence: LSSCDLCSDVQGCRPCED
C4-Carboxymethyl (58.00548 Da), C7-Carboxymethyl (58.00548 Da), C13-Carboxymethyl (58.00548 Da), C16-Carboxymethyl (58.00548 Da), D18-GPI-HF (43.04220 Da)
Charge: +2, Monoisotopic m/z: 1102.91064 Da (+2.63 mmu/+2.38 ppm), MH+: 2204.81401 Da, RT: 16.53 min,
Identified with: Mascot (v1.30); IonScore:84, Exp Value:1.4E-008, Matched Ions: 10/174
Fragment Match Tolerance: 0.5 Da
Protein References (1):
- CD109 antigen OS=Homo sapiens GN=CD109 PE=1 SV=2 - [CD109_HUMAN]

## Slide 18
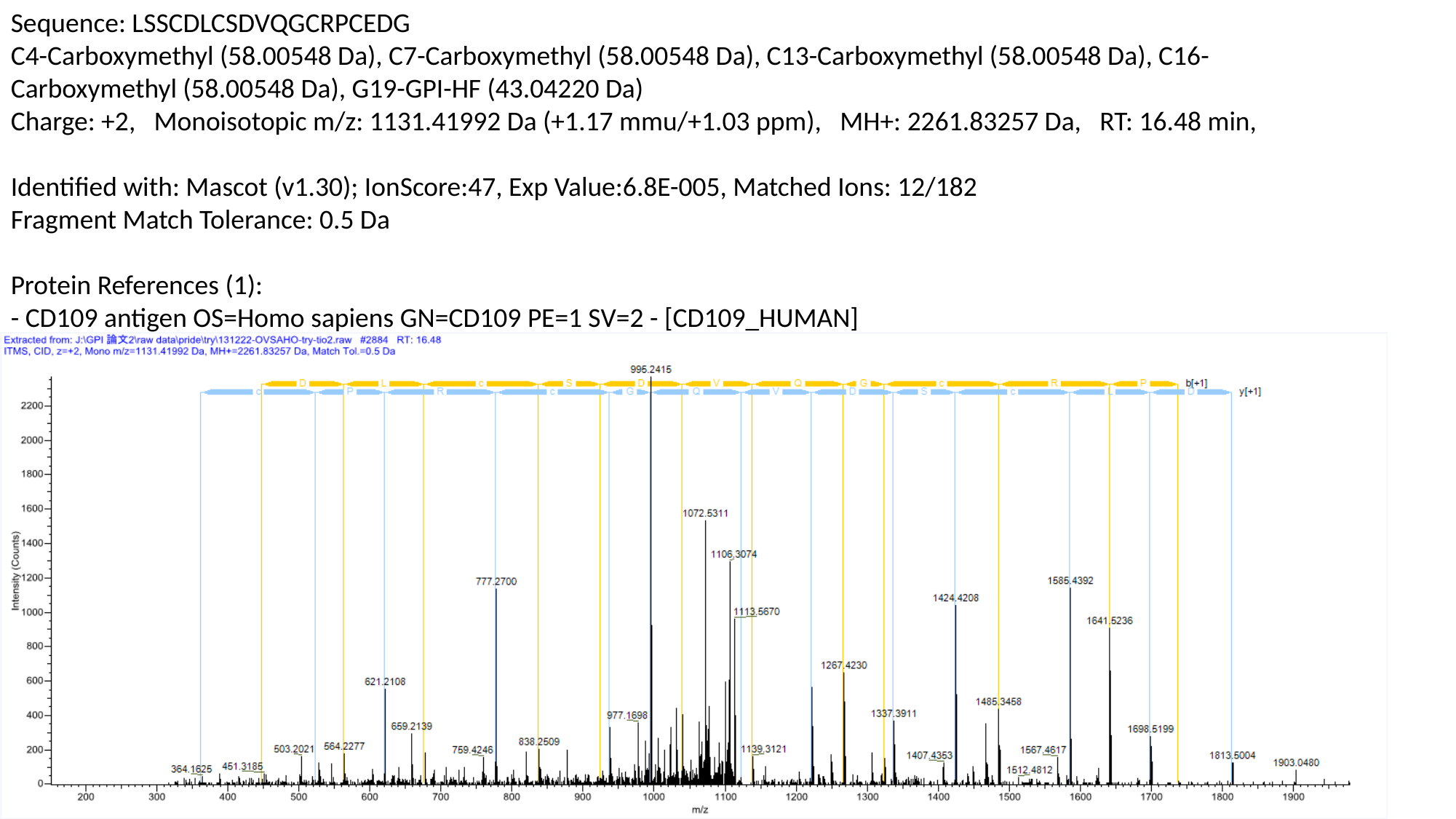

Sequence: LSSCDLCSDVQGCRPCEDG
C4-Carboxymethyl (58.00548 Da), C7-Carboxymethyl (58.00548 Da), C13-Carboxymethyl (58.00548 Da), C16-Carboxymethyl (58.00548 Da), G19-GPI-HF (43.04220 Da)
Charge: +2, Monoisotopic m/z: 1131.41992 Da (+1.17 mmu/+1.03 ppm), MH+: 2261.83257 Da, RT: 16.48 min,
Identified with: Mascot (v1.30); IonScore:47, Exp Value:6.8E-005, Matched Ions: 12/182
Fragment Match Tolerance: 0.5 Da
Protein References (1):
- CD109 antigen OS=Homo sapiens GN=CD109 PE=1 SV=2 - [CD109_HUMAN]

## Slide 19
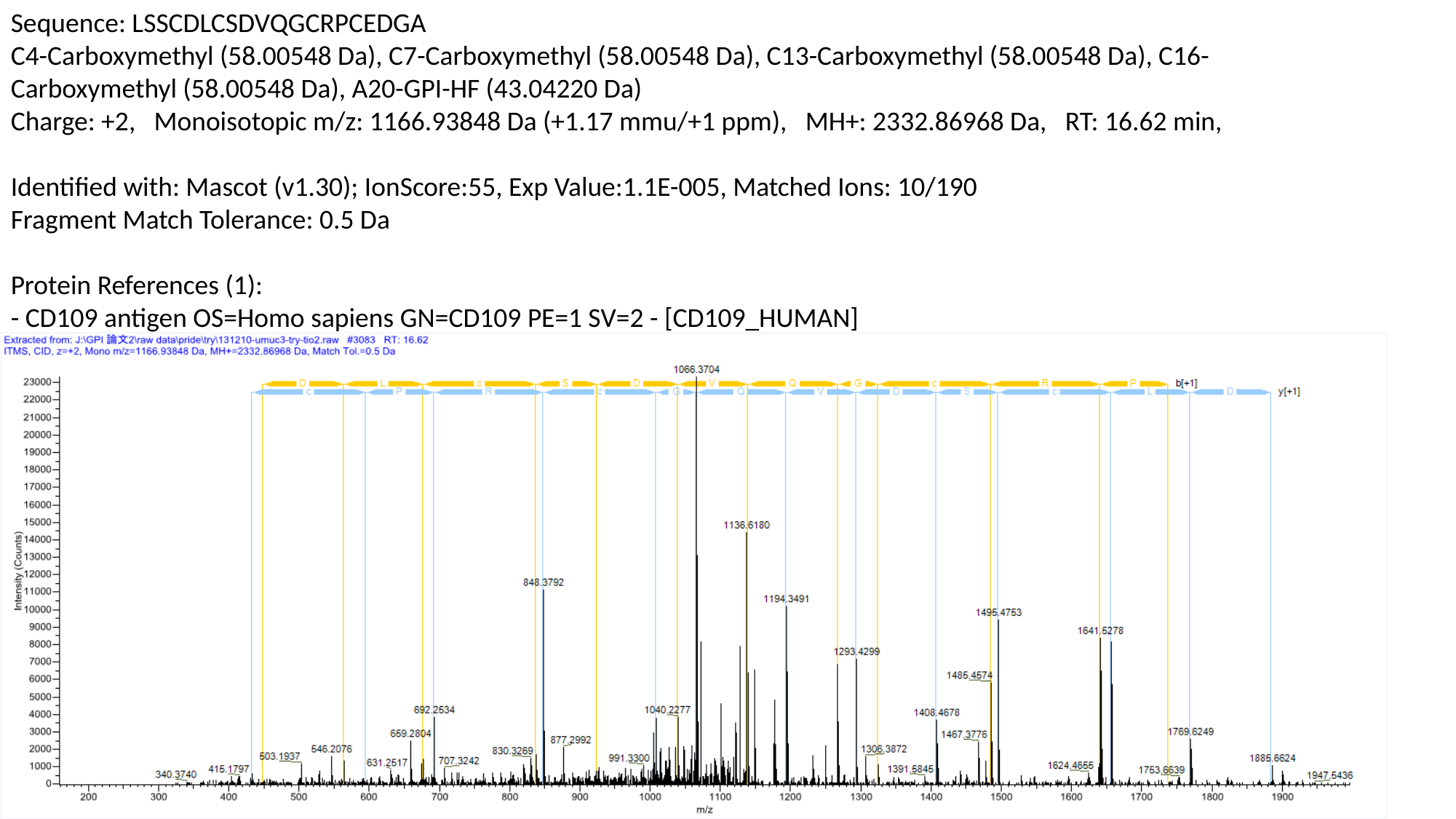

Sequence: LSSCDLCSDVQGCRPCEDGA
C4-Carboxymethyl (58.00548 Da), C7-Carboxymethyl (58.00548 Da), C13-Carboxymethyl (58.00548 Da), C16-Carboxymethyl (58.00548 Da), A20-GPI-HF (43.04220 Da)
Charge: +2, Monoisotopic m/z: 1166.93848 Da (+1.17 mmu/+1 ppm), MH+: 2332.86968 Da, RT: 16.62 min,
Identified with: Mascot (v1.30); IonScore:55, Exp Value:1.1E-005, Matched Ions: 10/190
Fragment Match Tolerance: 0.5 Da
Protein References (1):
- CD109 antigen OS=Homo sapiens GN=CD109 PE=1 SV=2 - [CD109_HUMAN]

## Slide 20
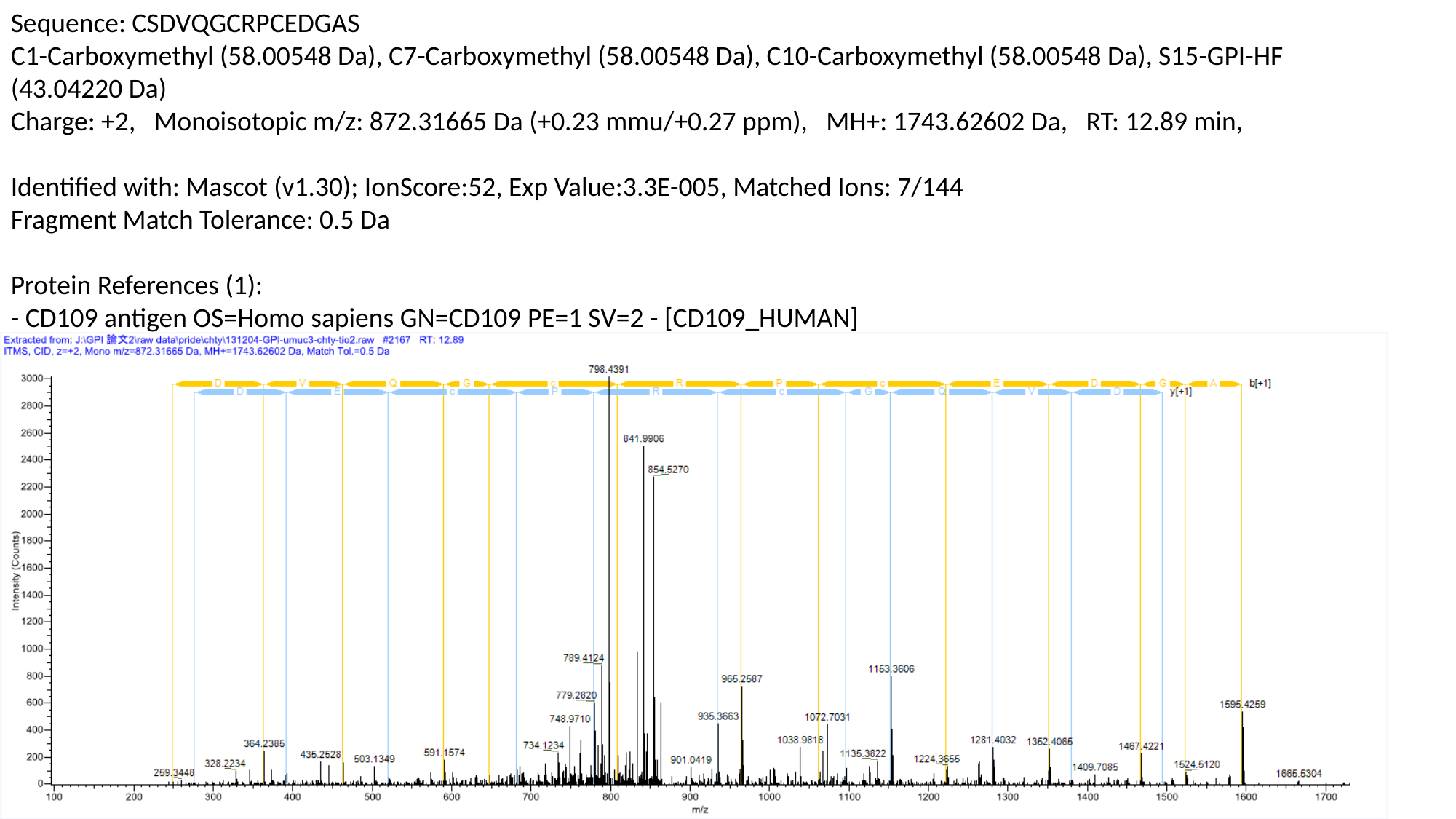

Sequence: CSDVQGCRPCEDGAS
C1-Carboxymethyl (58.00548 Da), C7-Carboxymethyl (58.00548 Da), C10-Carboxymethyl (58.00548 Da), S15-GPI-HF (43.04220 Da)
Charge: +2, Monoisotopic m/z: 872.31665 Da (+0.23 mmu/+0.27 ppm), MH+: 1743.62602 Da, RT: 12.89 min,
Identified with: Mascot (v1.30); IonScore:52, Exp Value:3.3E-005, Matched Ions: 7/144
Fragment Match Tolerance: 0.5 Da
Protein References (1):
- CD109 antigen OS=Homo sapiens GN=CD109 PE=1 SV=2 - [CD109_HUMAN]

## Slide 21
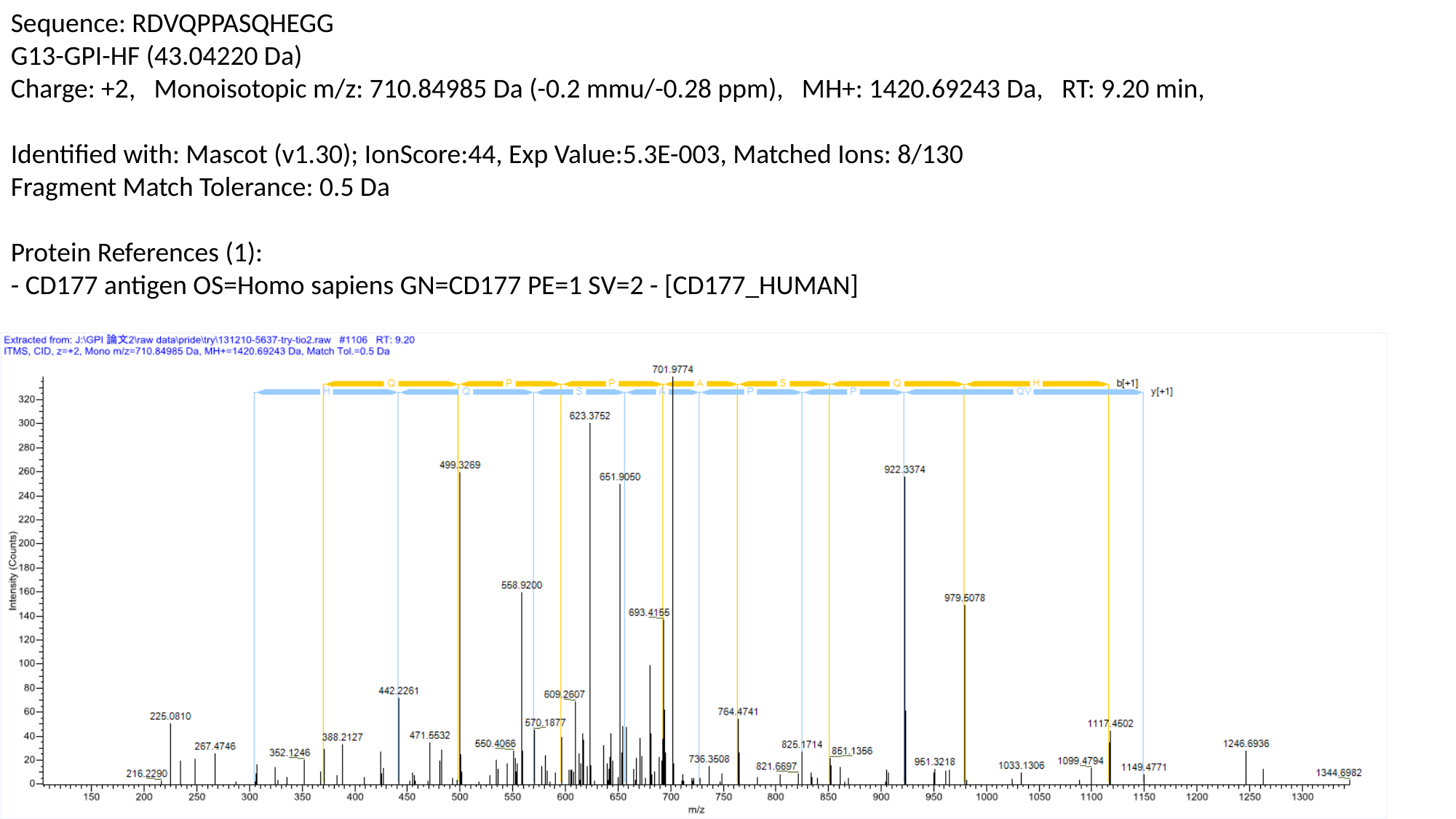

Sequence: RDVQPPASQHEGG
G13-GPI-HF (43.04220 Da)
Charge: +2, Monoisotopic m/z: 710.84985 Da (-0.2 mmu/-0.28 ppm), MH+: 1420.69243 Da, RT: 9.20 min,
Identified with: Mascot (v1.30); IonScore:44, Exp Value:5.3E-003, Matched Ions: 8/130
Fragment Match Tolerance: 0.5 Da
Protein References (1):
- CD177 antigen OS=Homo sapiens GN=CD177 PE=1 SV=2 - [CD177_HUMAN]

## Slide 22
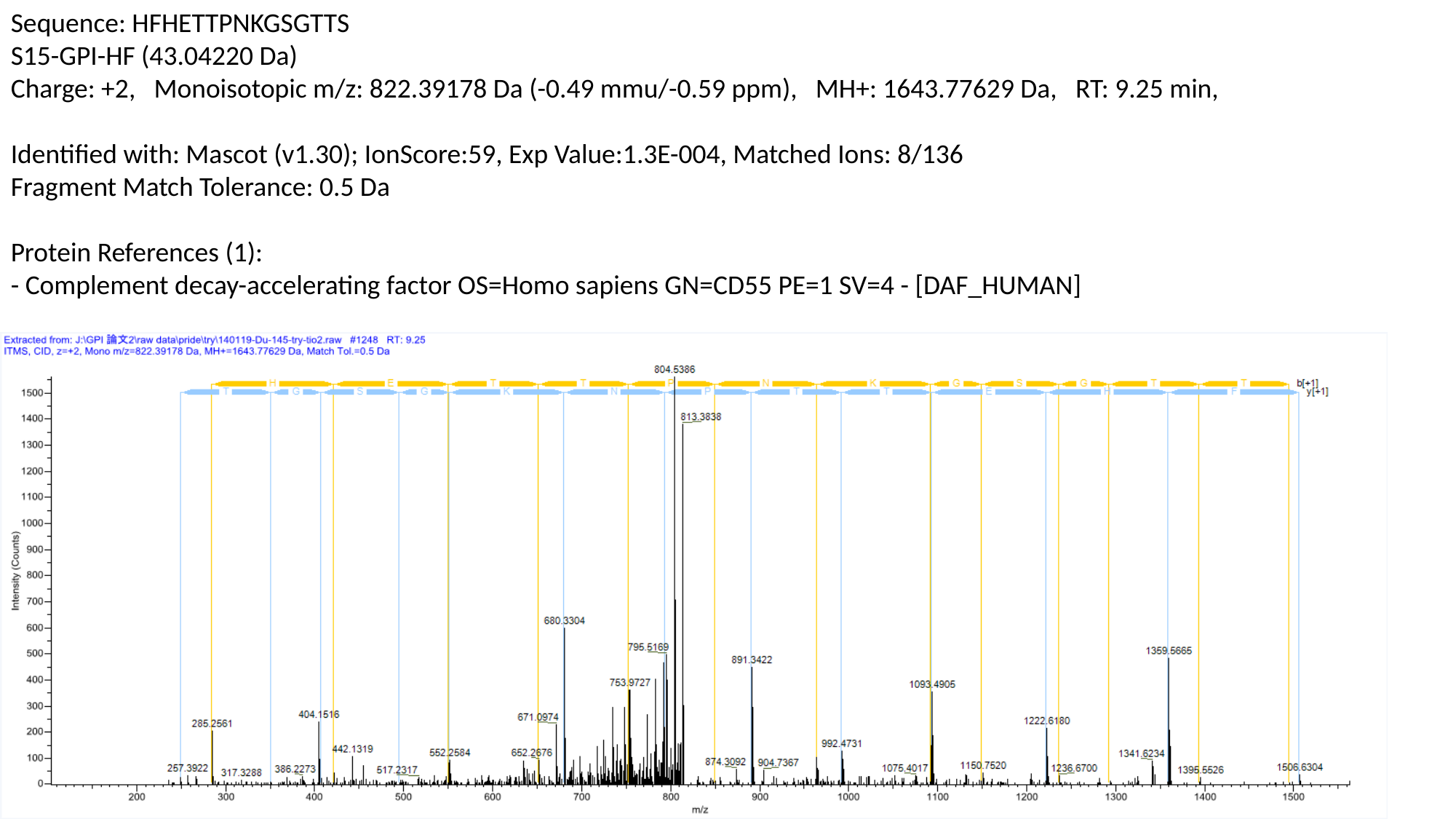

Sequence: HFHETTPNKGSGTTS
S15-GPI-HF (43.04220 Da)
Charge: +2, Monoisotopic m/z: 822.39178 Da (-0.49 mmu/-0.59 ppm), MH+: 1643.77629 Da, RT: 9.25 min,
Identified with: Mascot (v1.30); IonScore:59, Exp Value:1.3E-004, Matched Ions: 8/136
Fragment Match Tolerance: 0.5 Da
Protein References (1):
- Complement decay-accelerating factor OS=Homo sapiens GN=CD55 PE=1 SV=4 - [DAF_HUMAN]

## Slide 23
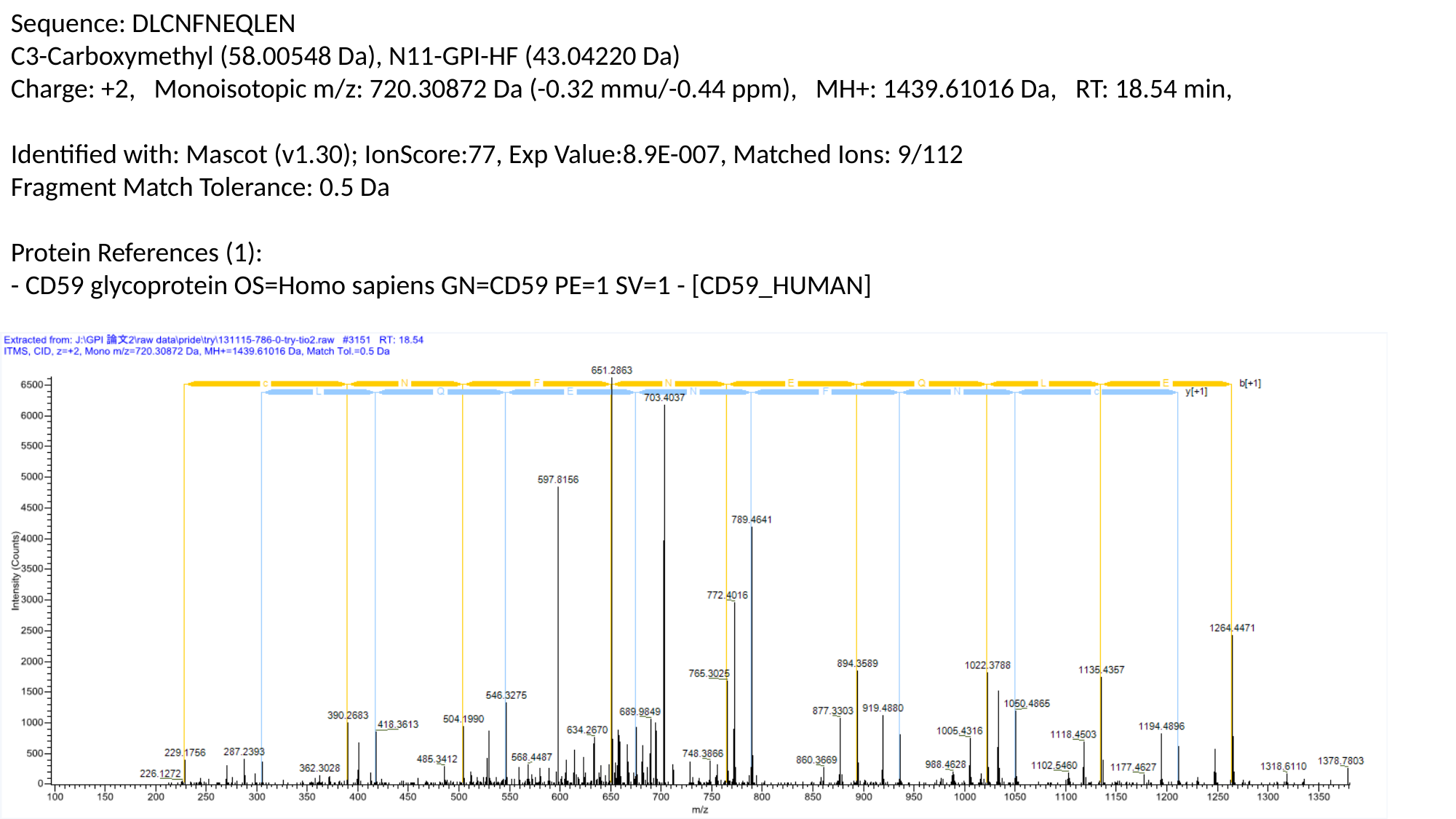

Sequence: DLCNFNEQLEN
C3-Carboxymethyl (58.00548 Da), N11-GPI-HF (43.04220 Da)
Charge: +2, Monoisotopic m/z: 720.30872 Da (-0.32 mmu/-0.44 ppm), MH+: 1439.61016 Da, RT: 18.54 min,
Identified with: Mascot (v1.30); IonScore:77, Exp Value:8.9E-007, Matched Ions: 9/112
Fragment Match Tolerance: 0.5 Da
Protein References (1):
- CD59 glycoprotein OS=Homo sapiens GN=CD59 PE=1 SV=1 - [CD59_HUMAN]

## Slide 24
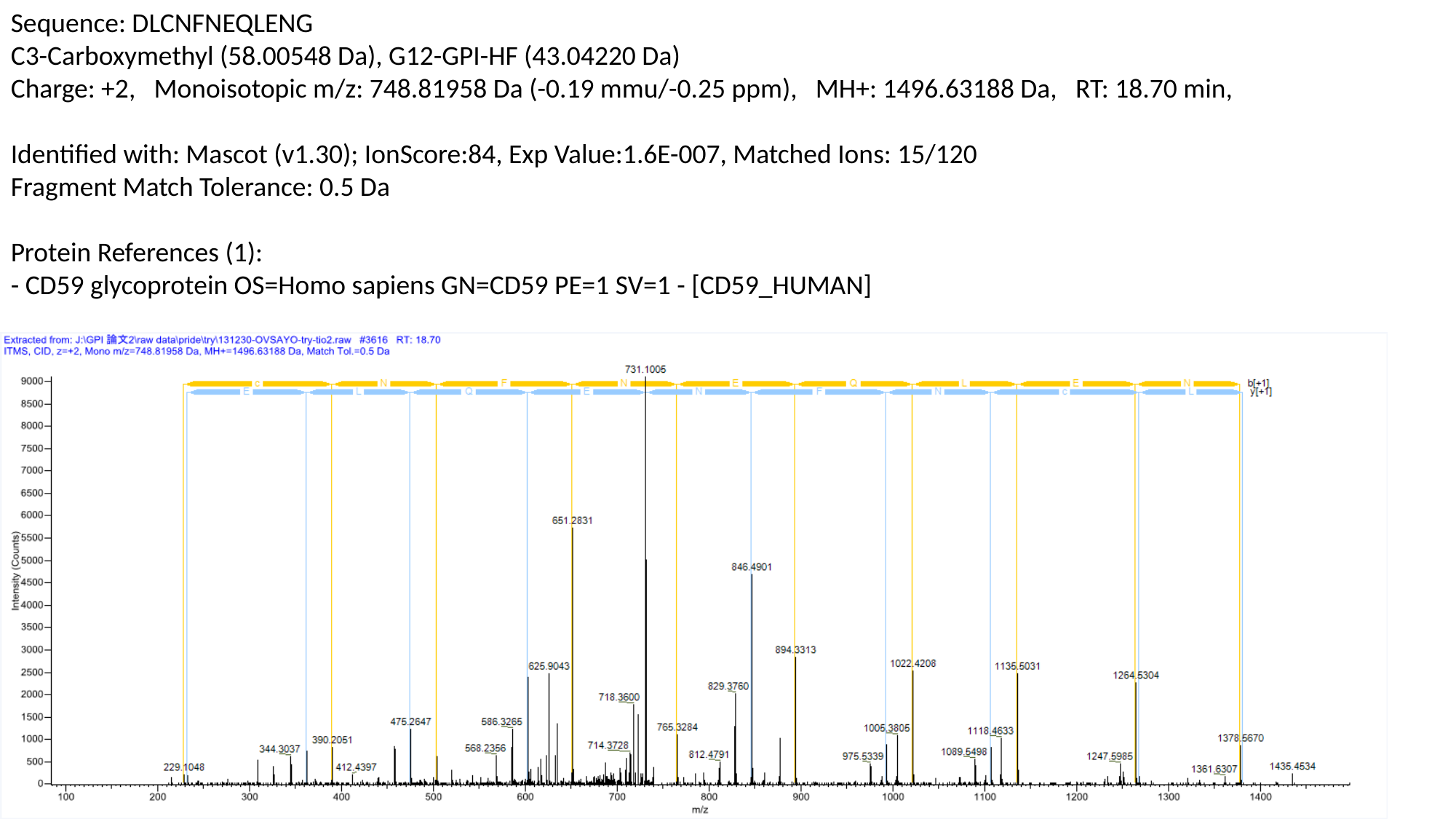

Sequence: DLCNFNEQLENG
C3-Carboxymethyl (58.00548 Da), G12-GPI-HF (43.04220 Da)
Charge: +2, Monoisotopic m/z: 748.81958 Da (-0.19 mmu/-0.25 ppm), MH+: 1496.63188 Da, RT: 18.70 min,
Identified with: Mascot (v1.30); IonScore:84, Exp Value:1.6E-007, Matched Ions: 15/120
Fragment Match Tolerance: 0.5 Da
Protein References (1):
- CD59 glycoprotein OS=Homo sapiens GN=CD59 PE=1 SV=1 - [CD59_HUMAN]

## Slide 25
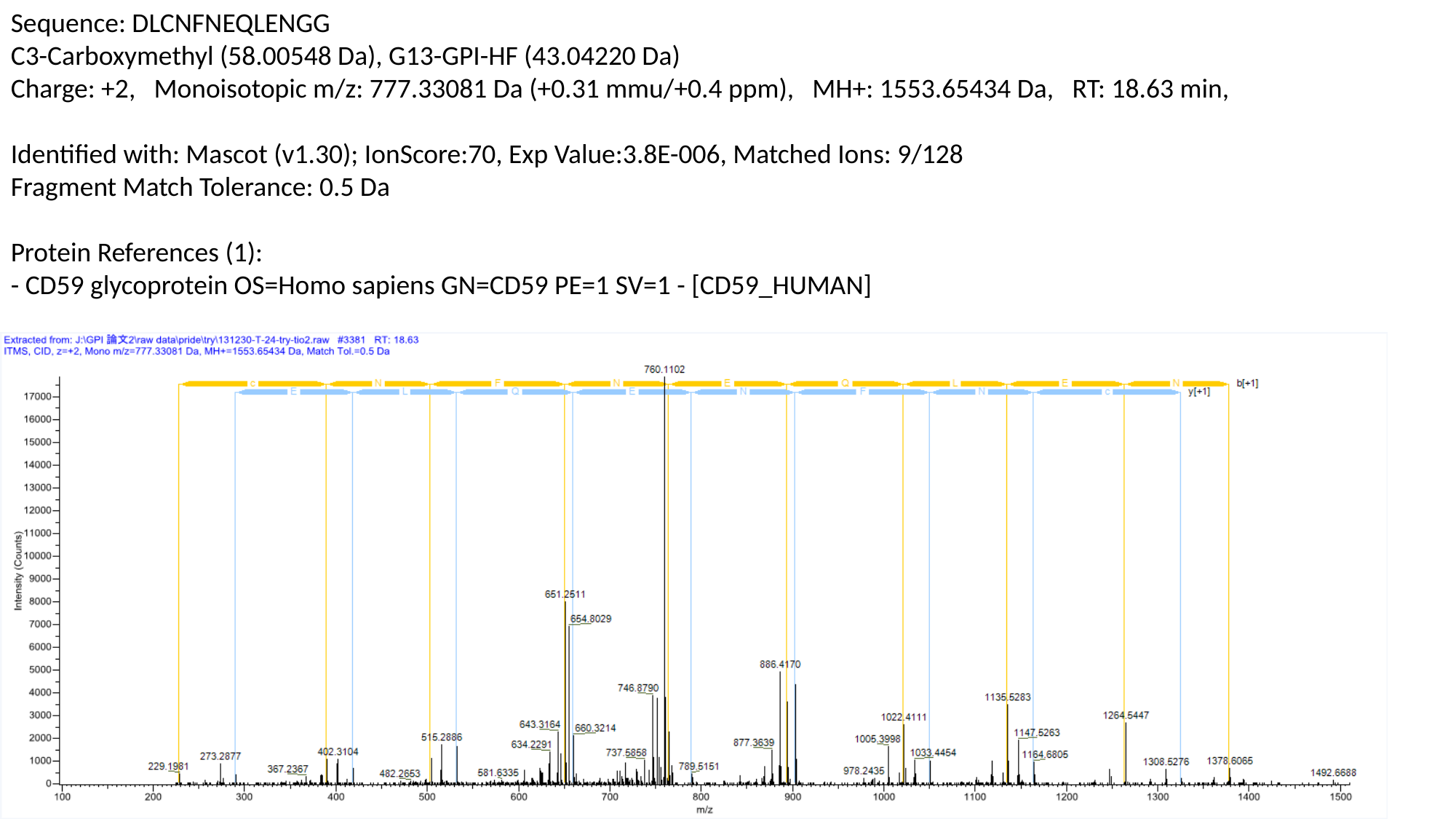

Sequence: DLCNFNEQLENGG
C3-Carboxymethyl (58.00548 Da), G13-GPI-HF (43.04220 Da)
Charge: +2, Monoisotopic m/z: 777.33081 Da (+0.31 mmu/+0.4 ppm), MH+: 1553.65434 Da, RT: 18.63 min,
Identified with: Mascot (v1.30); IonScore:70, Exp Value:3.8E-006, Matched Ions: 9/128
Fragment Match Tolerance: 0.5 Da
Protein References (1):
- CD59 glycoprotein OS=Homo sapiens GN=CD59 PE=1 SV=1 - [CD59_HUMAN]

## Slide 26
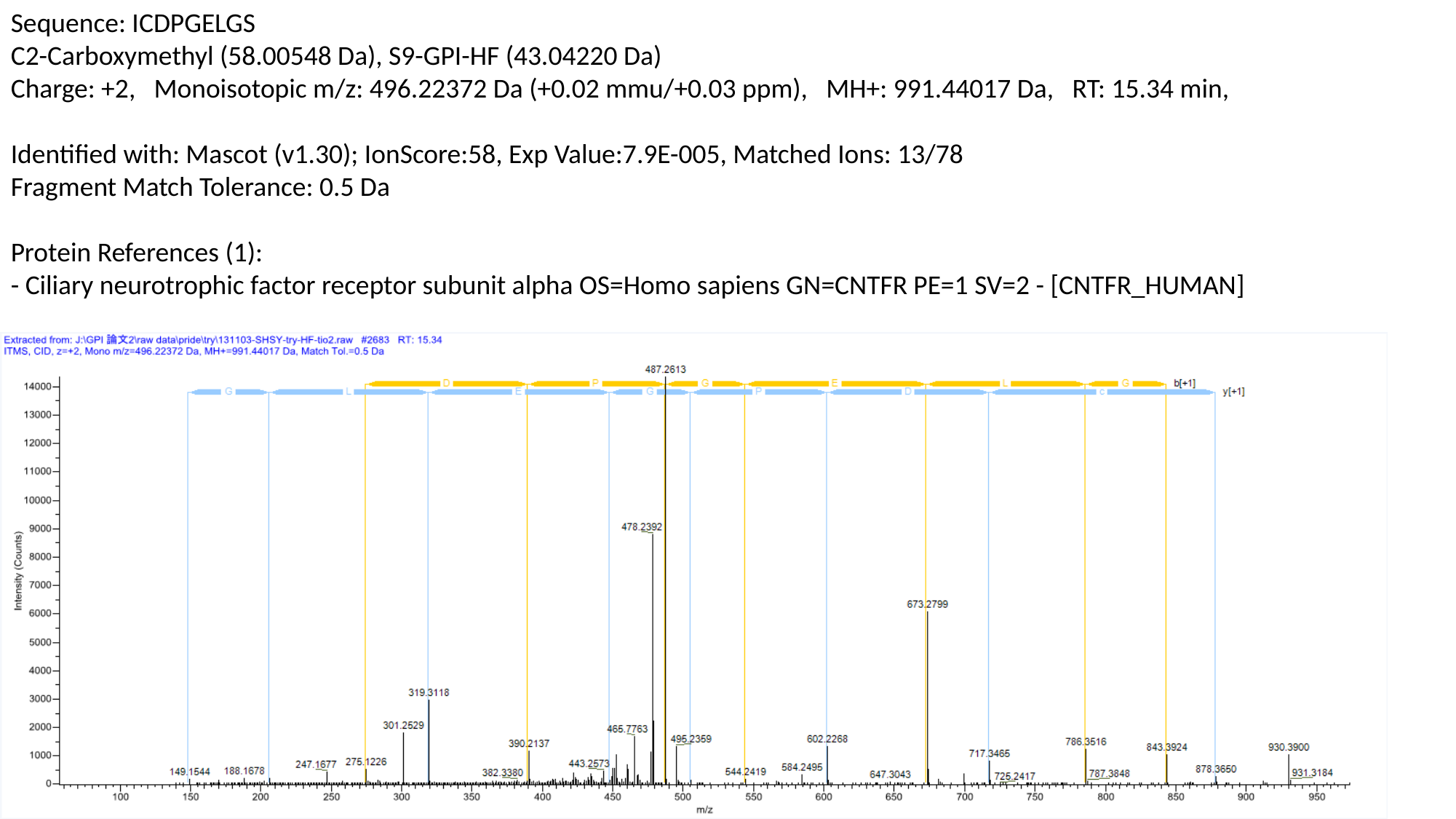

Sequence: ICDPGELGS
C2-Carboxymethyl (58.00548 Da), S9-GPI-HF (43.04220 Da)
Charge: +2, Monoisotopic m/z: 496.22372 Da (+0.02 mmu/+0.03 ppm), MH+: 991.44017 Da, RT: 15.34 min,
Identified with: Mascot (v1.30); IonScore:58, Exp Value:7.9E-005, Matched Ions: 13/78
Fragment Match Tolerance: 0.5 Da
Protein References (1):
- Ciliary neurotrophic factor receptor subunit alpha OS=Homo sapiens GN=CNTFR PE=1 SV=2 - [CNTFR_HUMAN]

## Slide 27
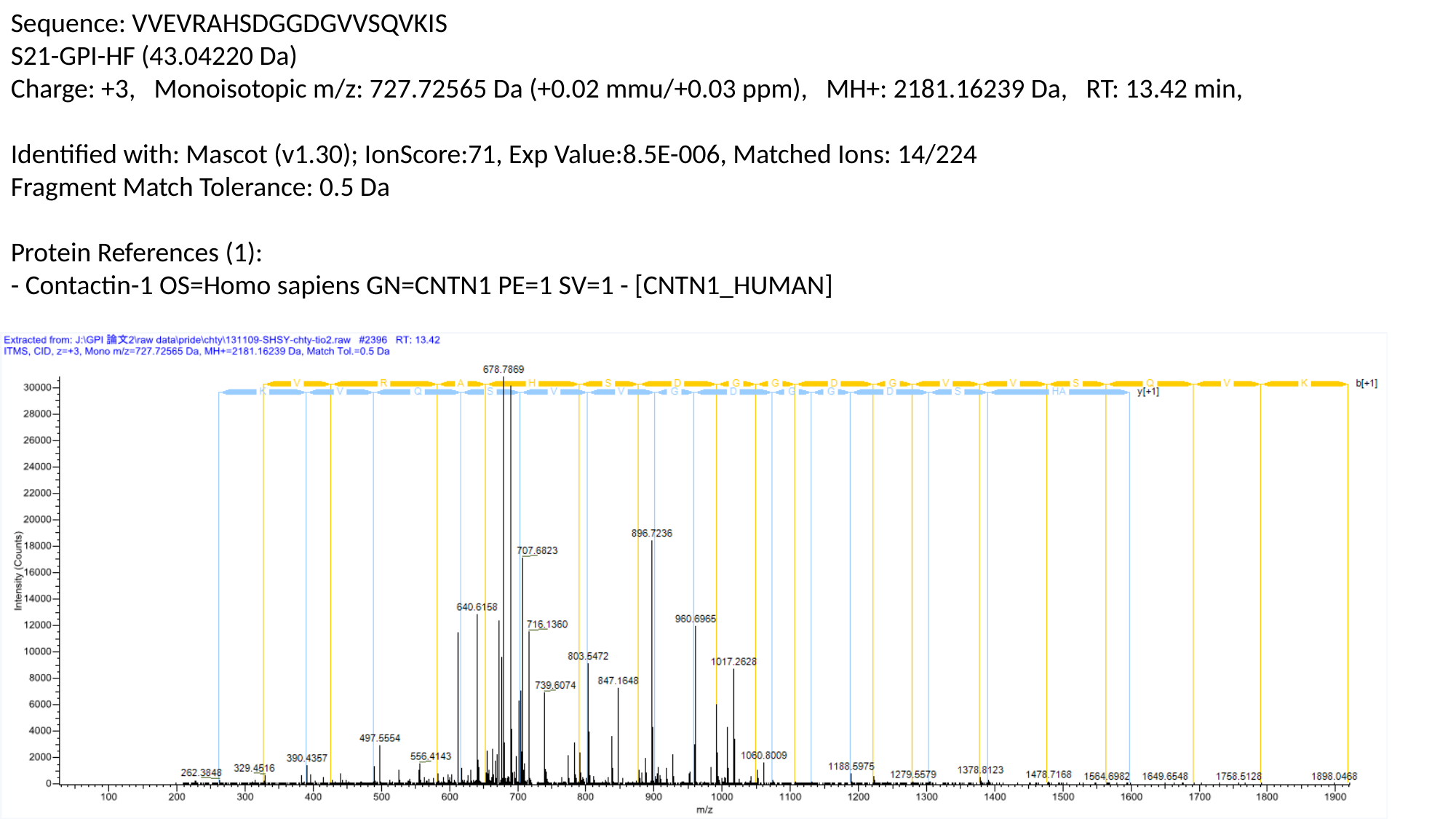

Sequence: VVEVRAHSDGGDGVVSQVKIS
S21-GPI-HF (43.04220 Da)
Charge: +3, Monoisotopic m/z: 727.72565 Da (+0.02 mmu/+0.03 ppm), MH+: 2181.16239 Da, RT: 13.42 min,
Identified with: Mascot (v1.30); IonScore:71, Exp Value:8.5E-006, Matched Ions: 14/224
Fragment Match Tolerance: 0.5 Da
Protein References (1):
- Contactin-1 OS=Homo sapiens GN=CNTN1 PE=1 SV=1 - [CNTN1_HUMAN]

## Slide 28
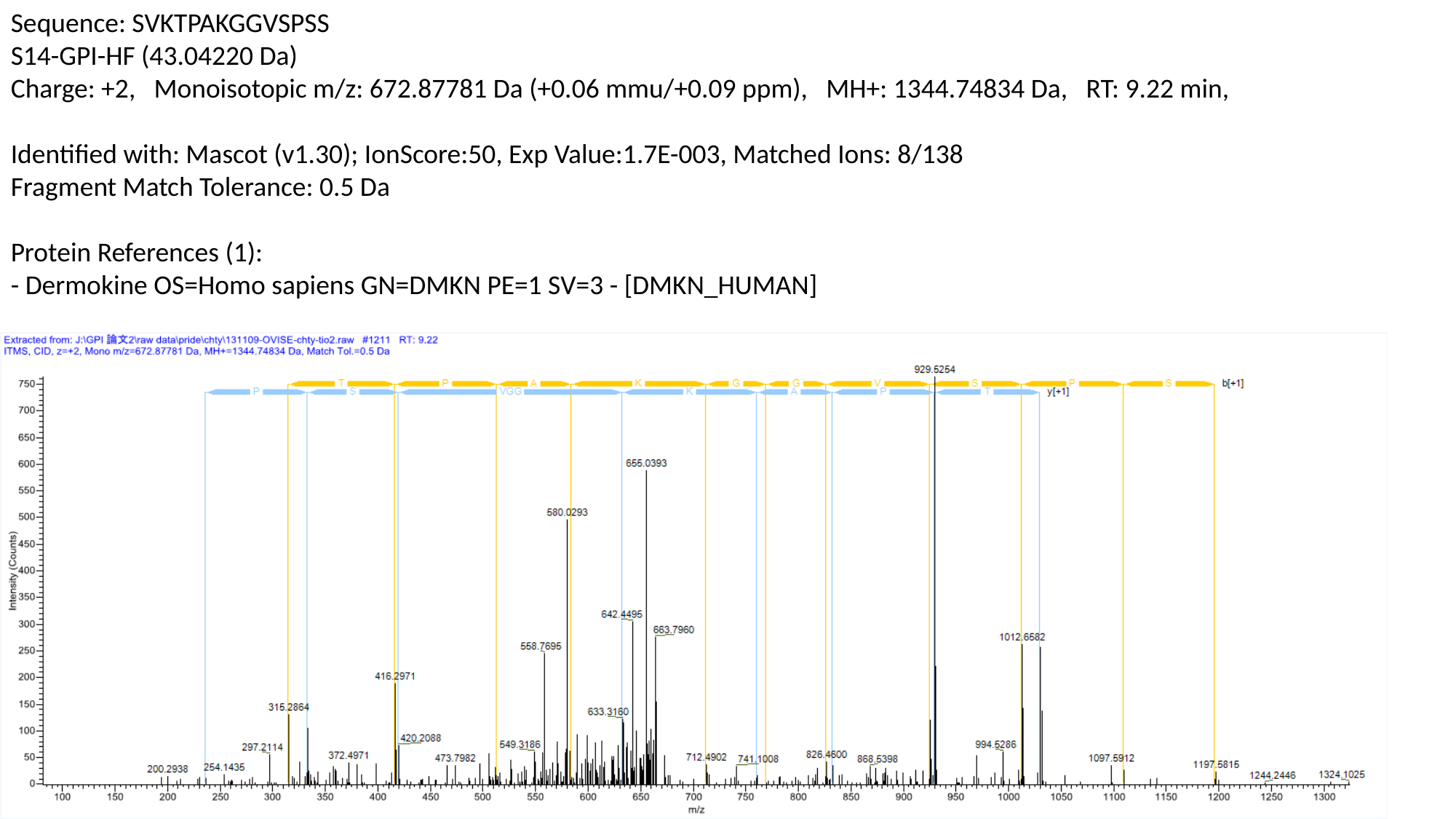

Sequence: SVKTPAKGGVSPSS
S14-GPI-HF (43.04220 Da)
Charge: +2, Monoisotopic m/z: 672.87781 Da (+0.06 mmu/+0.09 ppm), MH+: 1344.74834 Da, RT: 9.22 min,
Identified with: Mascot (v1.30); IonScore:50, Exp Value:1.7E-003, Matched Ions: 8/138
Fragment Match Tolerance: 0.5 Da
Protein References (1):
- Dermokine OS=Homo sapiens GN=DMKN PE=1 SV=3 - [DMKN_HUMAN]

## Slide 29
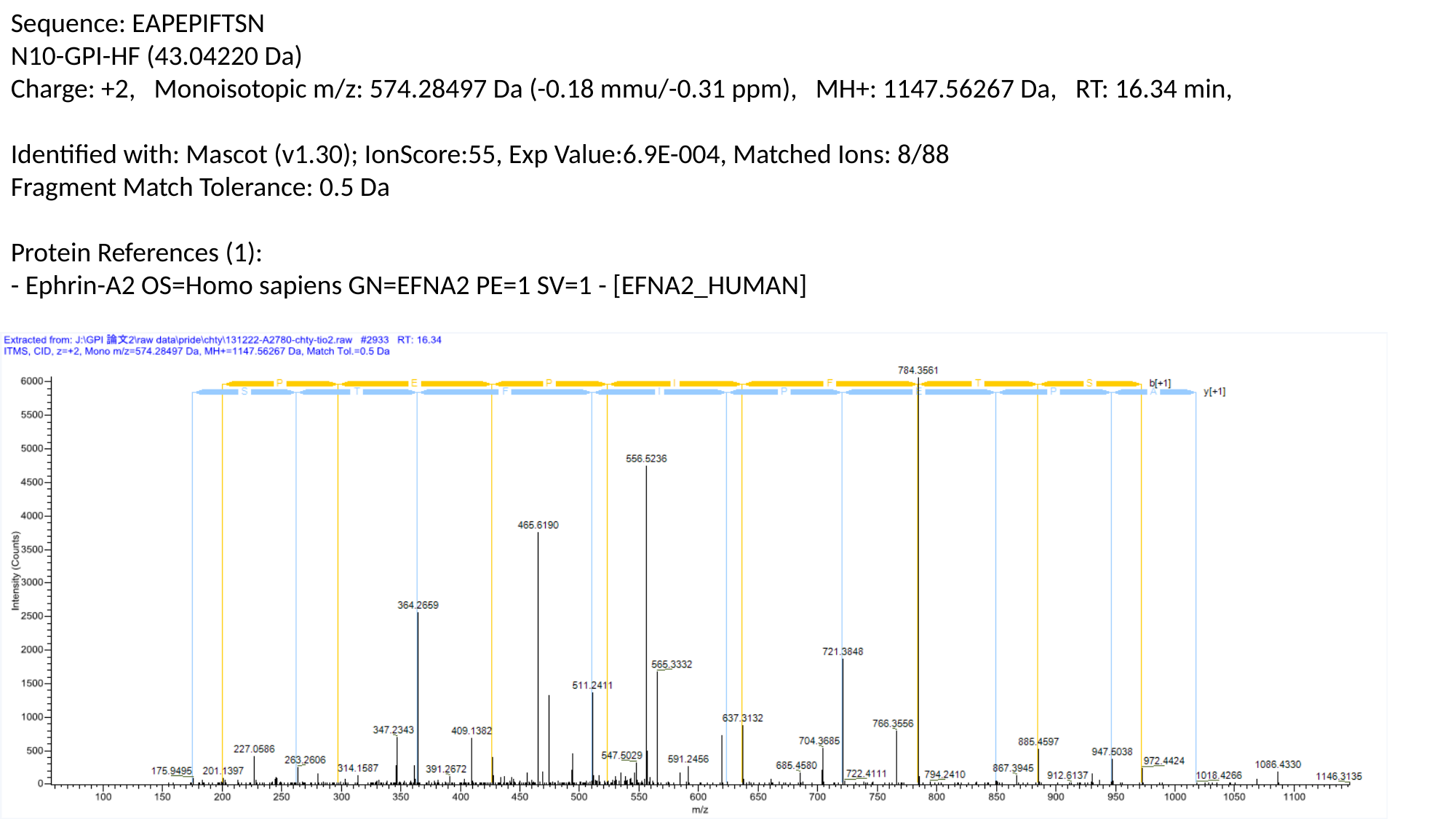

Sequence: EAPEPIFTSN
N10-GPI-HF (43.04220 Da)
Charge: +2, Monoisotopic m/z: 574.28497 Da (-0.18 mmu/-0.31 ppm), MH+: 1147.56267 Da, RT: 16.34 min,
Identified with: Mascot (v1.30); IonScore:55, Exp Value:6.9E-004, Matched Ions: 8/88
Fragment Match Tolerance: 0.5 Da
Protein References (1):
- Ephrin-A2 OS=Homo sapiens GN=EFNA2 PE=1 SV=1 - [EFNA2_HUMAN]

## Slide 30
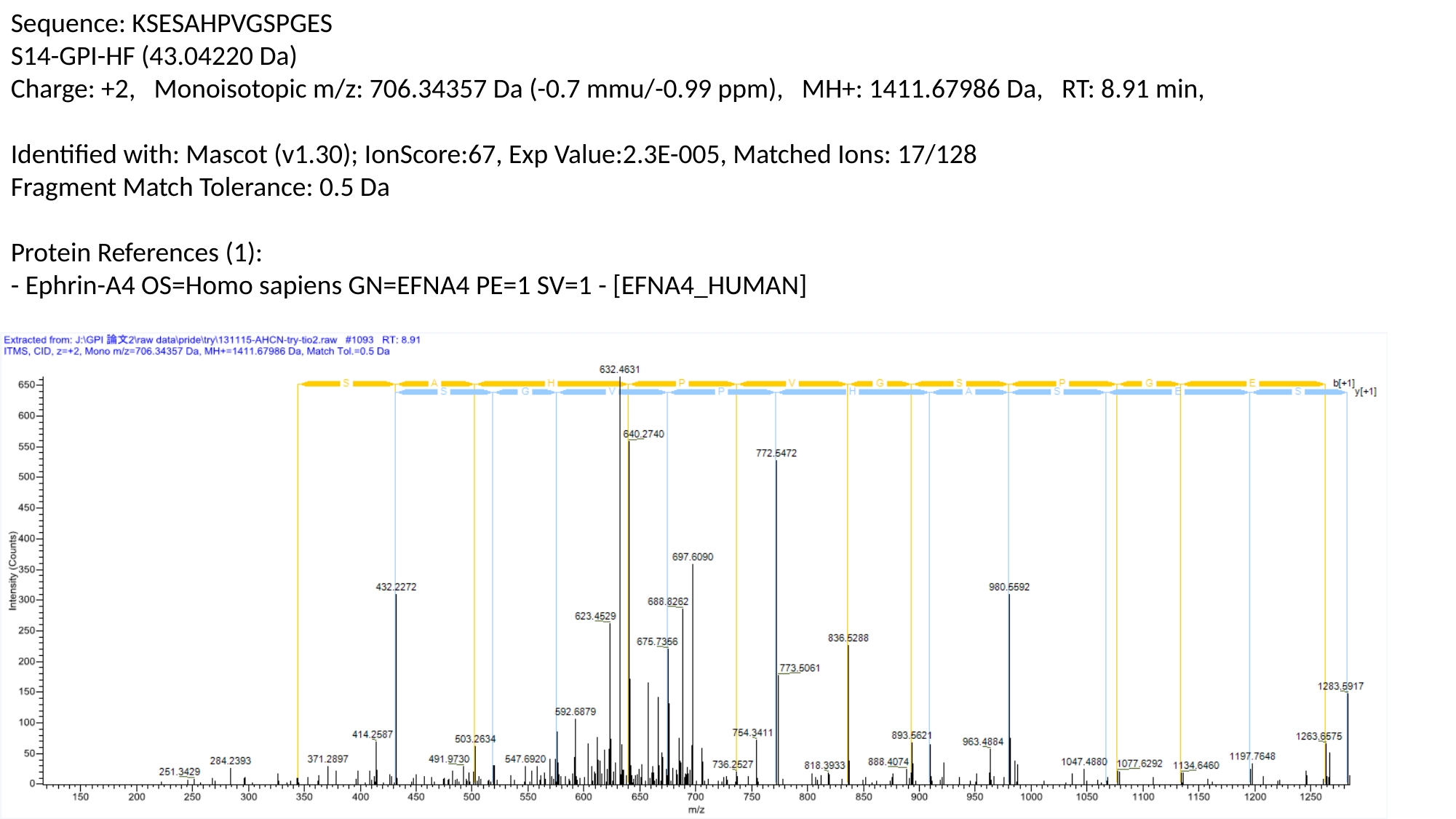

Sequence: KSESAHPVGSPGES
S14-GPI-HF (43.04220 Da)
Charge: +2, Monoisotopic m/z: 706.34357 Da (-0.7 mmu/-0.99 ppm), MH+: 1411.67986 Da, RT: 8.91 min,
Identified with: Mascot (v1.30); IonScore:67, Exp Value:2.3E-005, Matched Ions: 17/128
Fragment Match Tolerance: 0.5 Da
Protein References (1):
- Ephrin-A4 OS=Homo sapiens GN=EFNA4 PE=1 SV=1 - [EFNA4_HUMAN]

## Slide 31
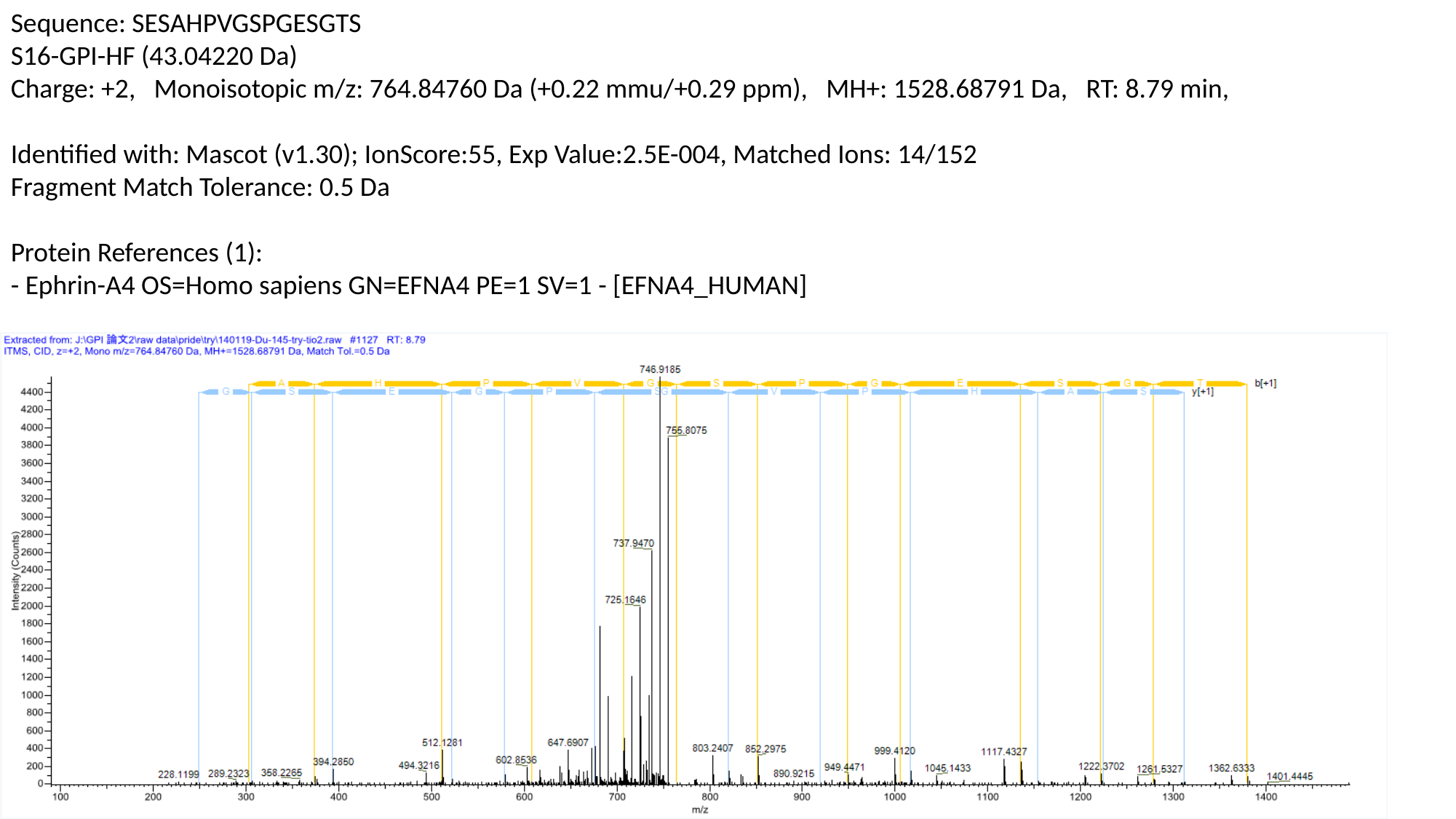

Sequence: SESAHPVGSPGESGTS
S16-GPI-HF (43.04220 Da)
Charge: +2, Monoisotopic m/z: 764.84760 Da (+0.22 mmu/+0.29 ppm), MH+: 1528.68791 Da, RT: 8.79 min,
Identified with: Mascot (v1.30); IonScore:55, Exp Value:2.5E-004, Matched Ions: 14/152
Fragment Match Tolerance: 0.5 Da
Protein References (1):
- Ephrin-A4 OS=Homo sapiens GN=EFNA4 PE=1 SV=1 - [EFNA4_HUMAN]

## Slide 32
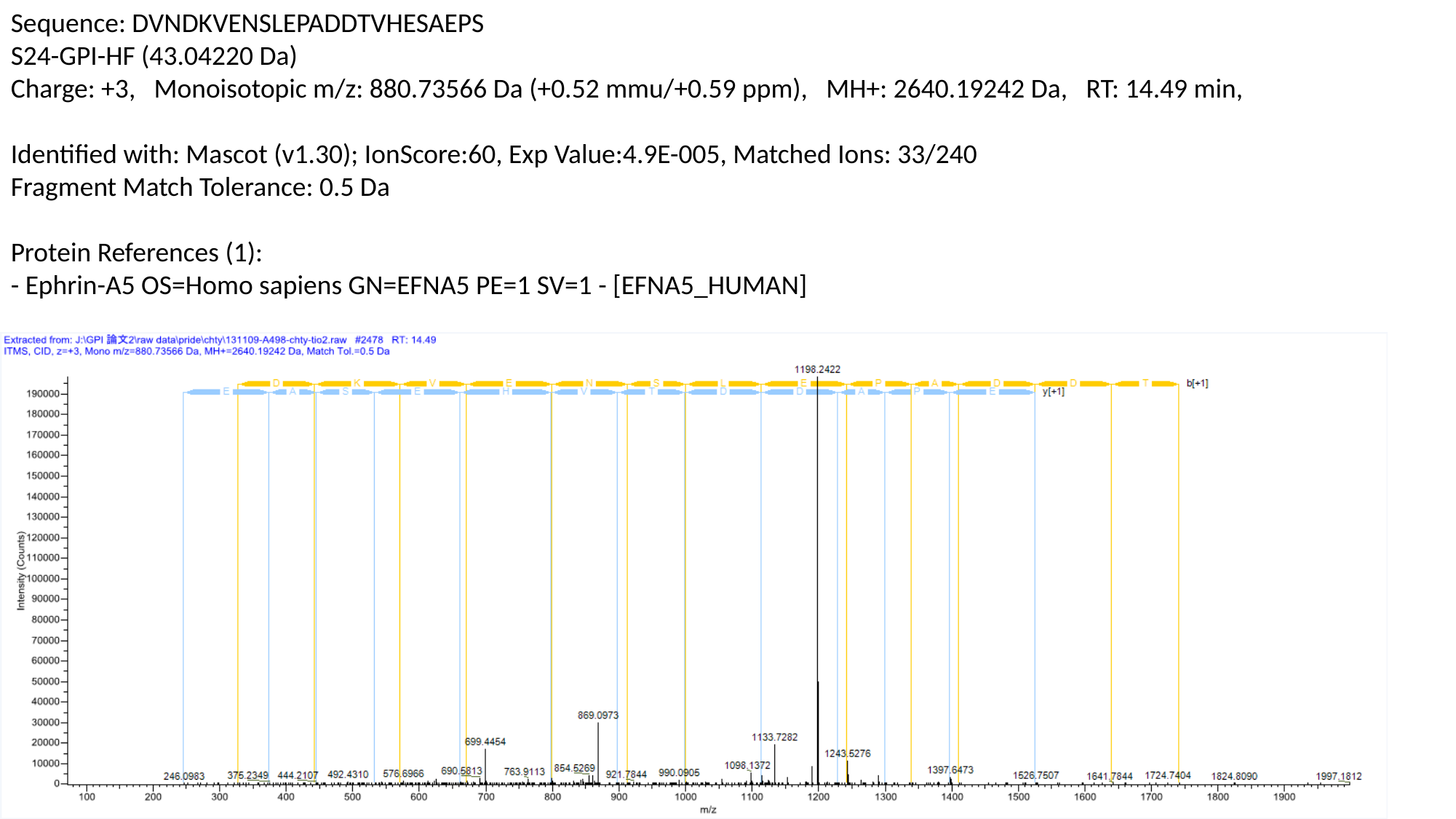

Sequence: DVNDKVENSLEPADDTVHESAEPS
S24-GPI-HF (43.04220 Da)
Charge: +3, Monoisotopic m/z: 880.73566 Da (+0.52 mmu/+0.59 ppm), MH+: 2640.19242 Da, RT: 14.49 min,
Identified with: Mascot (v1.30); IonScore:60, Exp Value:4.9E-005, Matched Ions: 33/240
Fragment Match Tolerance: 0.5 Da
Protein References (1):
- Ephrin-A5 OS=Homo sapiens GN=EFNA5 PE=1 SV=1 - [EFNA5_HUMAN]

## Slide 33
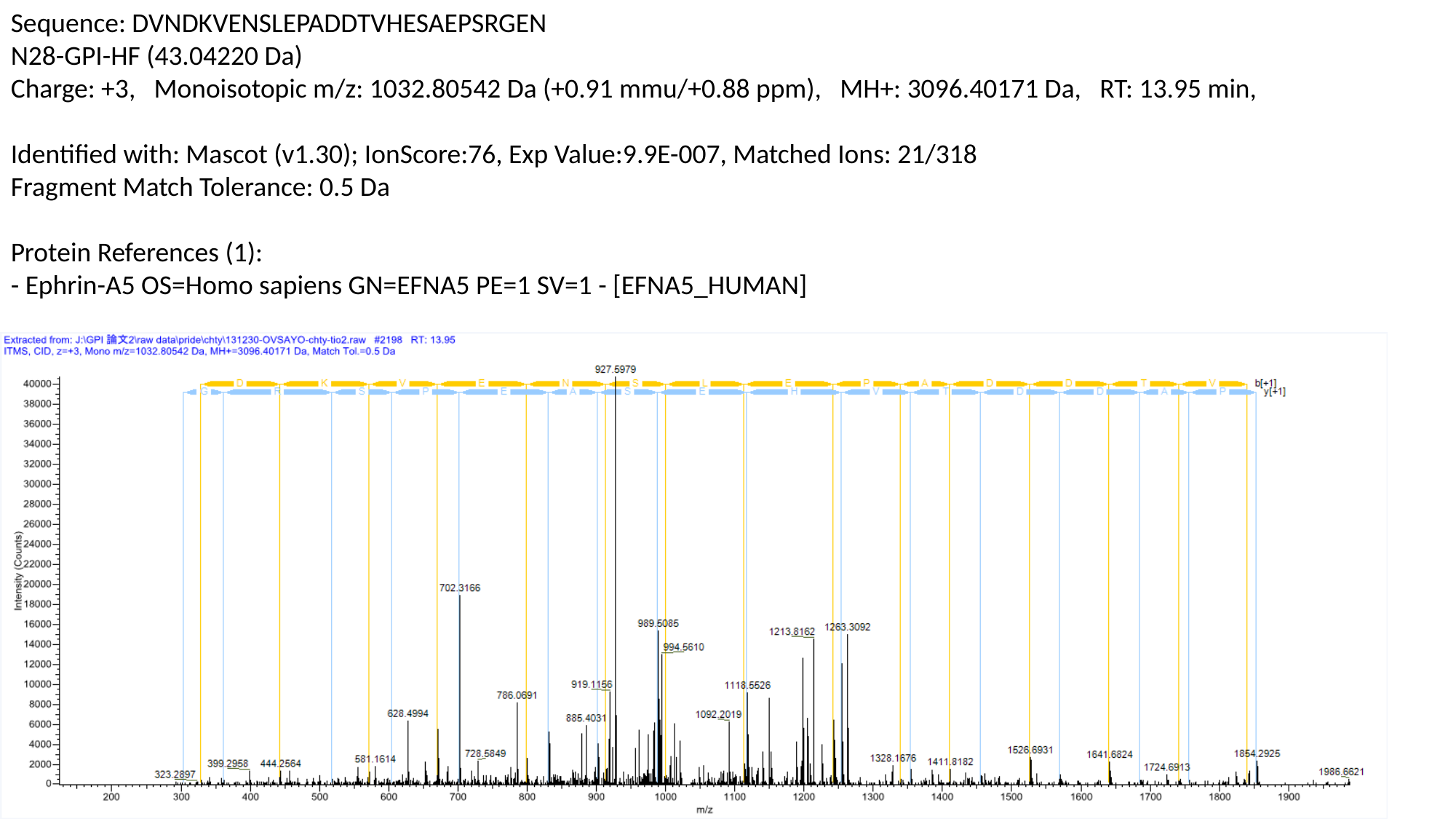

Sequence: DVNDKVENSLEPADDTVHESAEPSRGEN
N28-GPI-HF (43.04220 Da)
Charge: +3, Monoisotopic m/z: 1032.80542 Da (+0.91 mmu/+0.88 ppm), MH+: 3096.40171 Da, RT: 13.95 min,
Identified with: Mascot (v1.30); IonScore:76, Exp Value:9.9E-007, Matched Ions: 21/318
Fragment Match Tolerance: 0.5 Da
Protein References (1):
- Ephrin-A5 OS=Homo sapiens GN=EFNA5 PE=1 SV=1 - [EFNA5_HUMAN]

## Slide 34
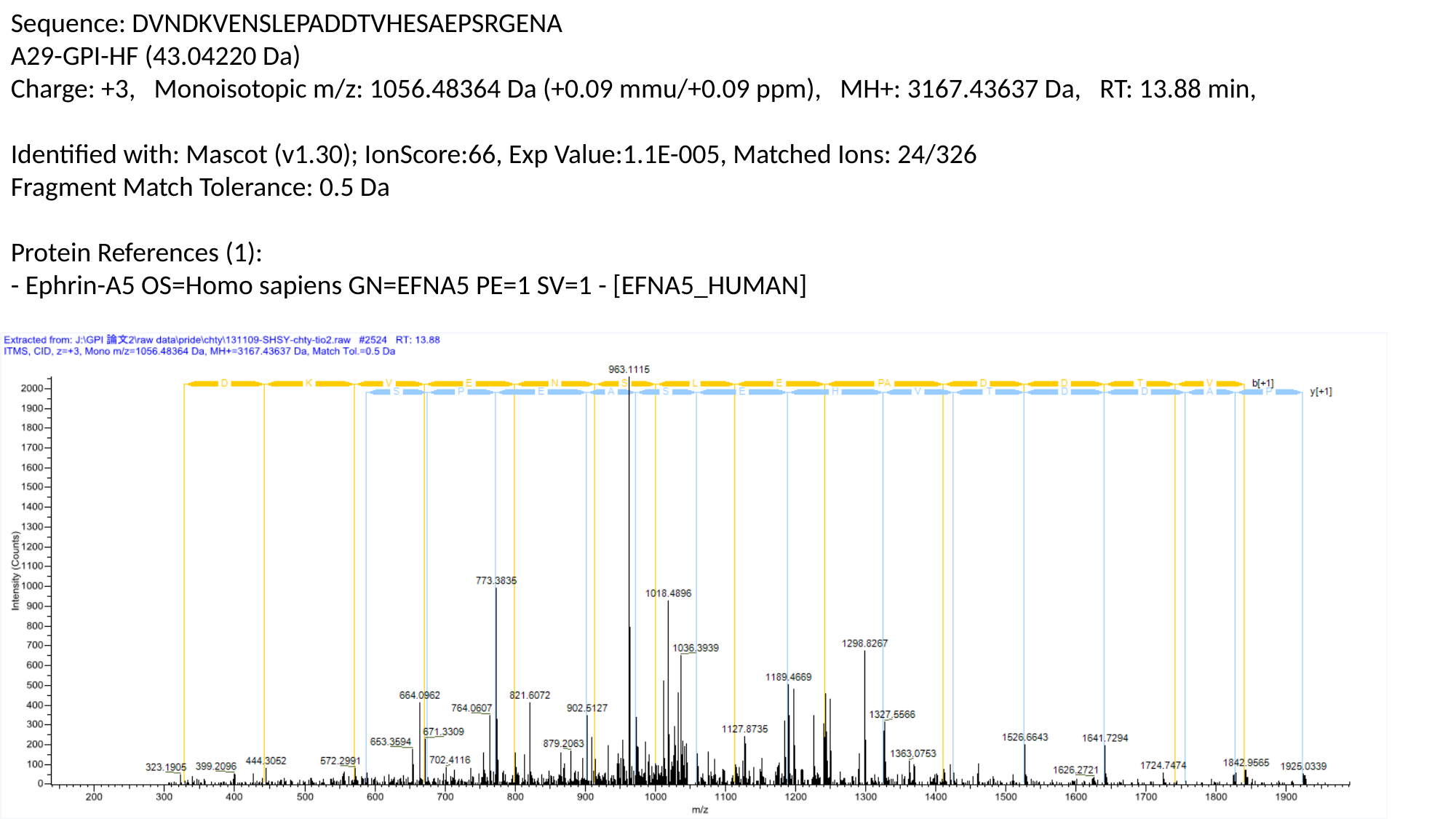

Sequence: DVNDKVENSLEPADDTVHESAEPSRGENA
A29-GPI-HF (43.04220 Da)
Charge: +3, Monoisotopic m/z: 1056.48364 Da (+0.09 mmu/+0.09 ppm), MH+: 3167.43637 Da, RT: 13.88 min,
Identified with: Mascot (v1.30); IonScore:66, Exp Value:1.1E-005, Matched Ions: 24/326
Fragment Match Tolerance: 0.5 Da
Protein References (1):
- Ephrin-A5 OS=Homo sapiens GN=EFNA5 PE=1 SV=1 - [EFNA5_HUMAN]

## Slide 35
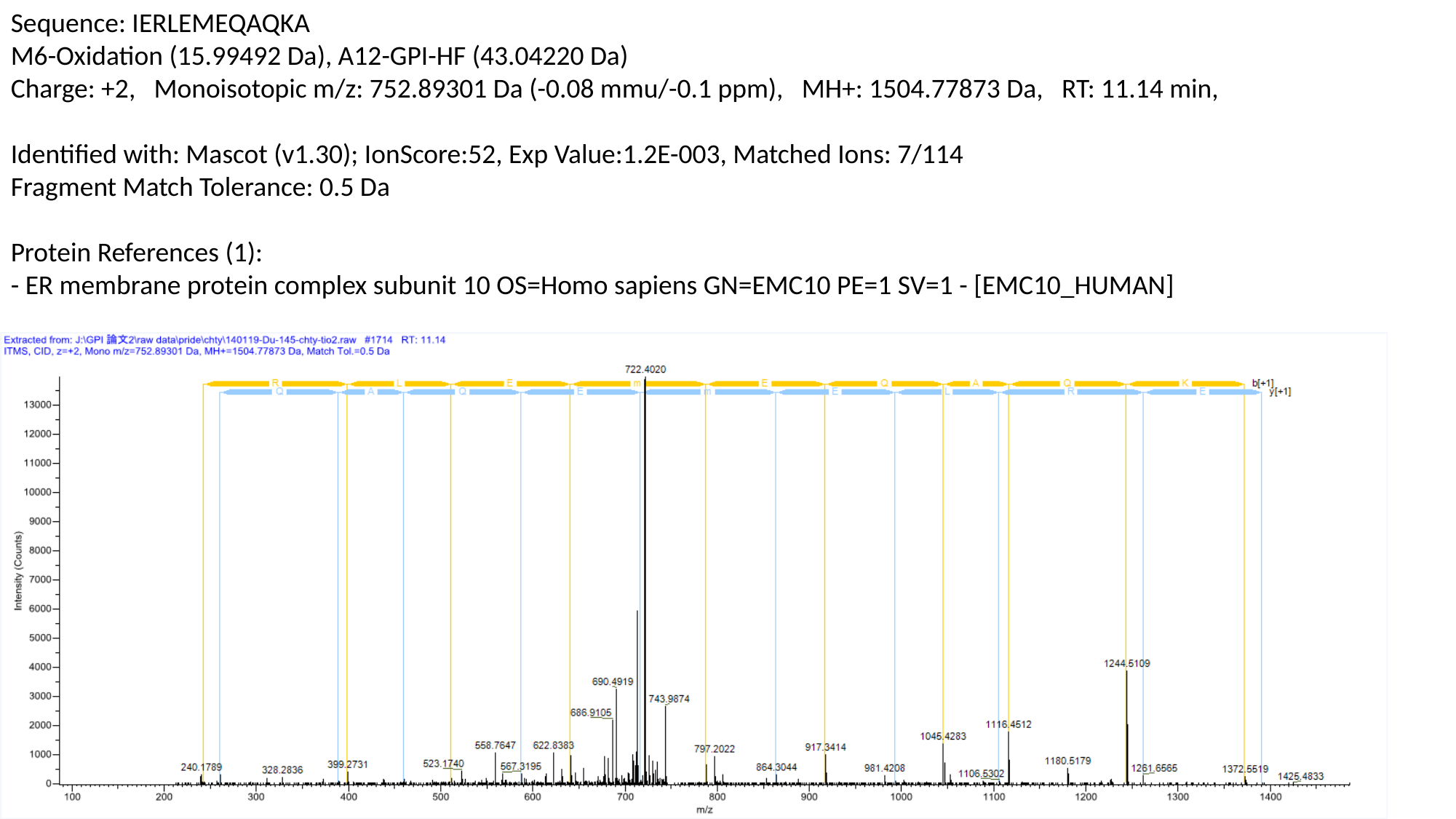

Sequence: IERLEMEQAQKA
M6-Oxidation (15.99492 Da), A12-GPI-HF (43.04220 Da)
Charge: +2, Monoisotopic m/z: 752.89301 Da (-0.08 mmu/-0.1 ppm), MH+: 1504.77873 Da, RT: 11.14 min,
Identified with: Mascot (v1.30); IonScore:52, Exp Value:1.2E-003, Matched Ions: 7/114
Fragment Match Tolerance: 0.5 Da
Protein References (1):
- ER membrane protein complex subunit 10 OS=Homo sapiens GN=EMC10 PE=1 SV=1 - [EMC10_HUMAN]

## Slide 36
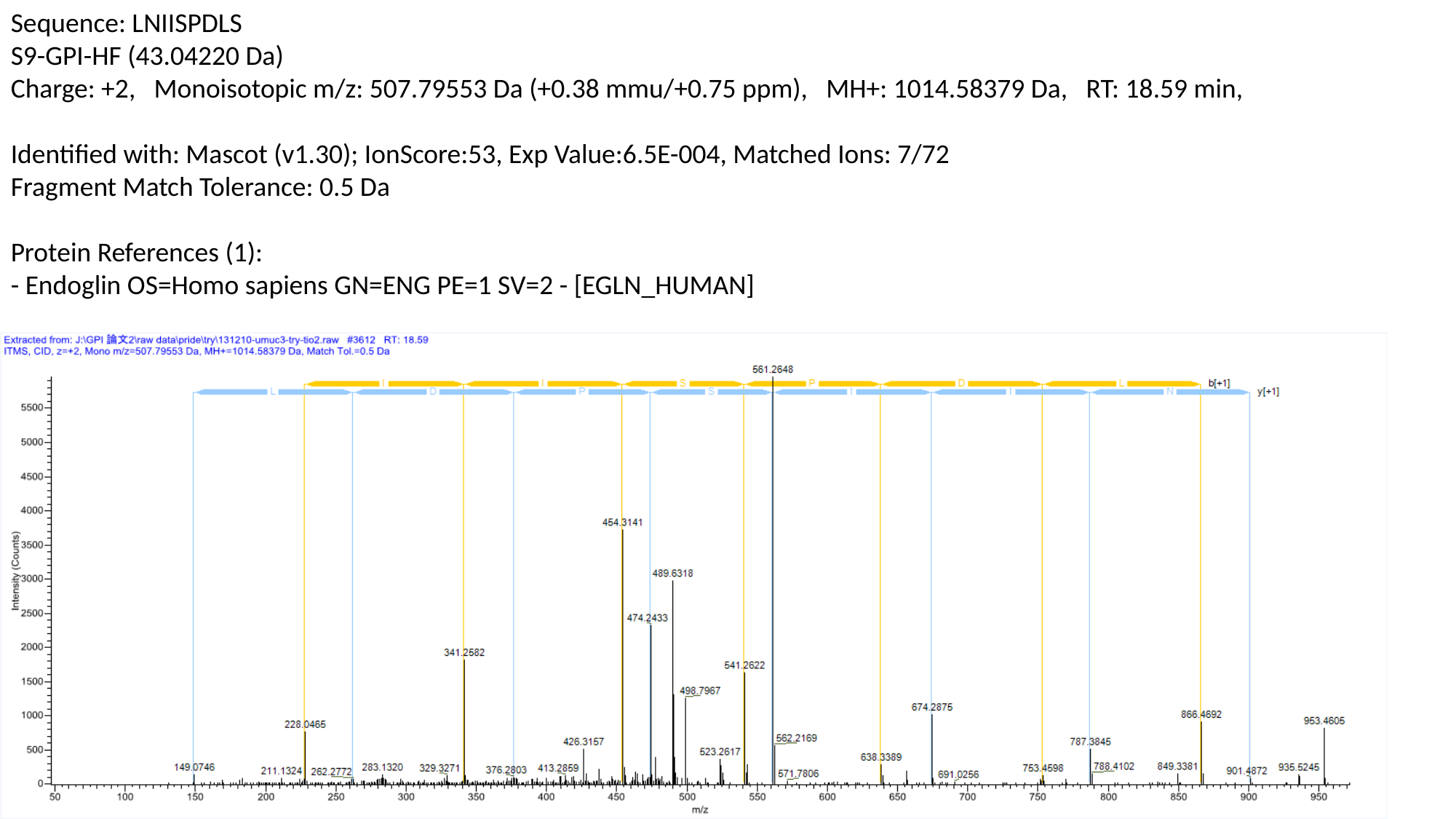

Sequence: LNIISPDLS
S9-GPI-HF (43.04220 Da)
Charge: +2, Monoisotopic m/z: 507.79553 Da (+0.38 mmu/+0.75 ppm), MH+: 1014.58379 Da, RT: 18.59 min,
Identified with: Mascot (v1.30); IonScore:53, Exp Value:6.5E-004, Matched Ions: 7/72
Fragment Match Tolerance: 0.5 Da
Protein References (1):
- Endoglin OS=Homo sapiens GN=ENG PE=1 SV=2 - [EGLN_HUMAN]

## Slide 37
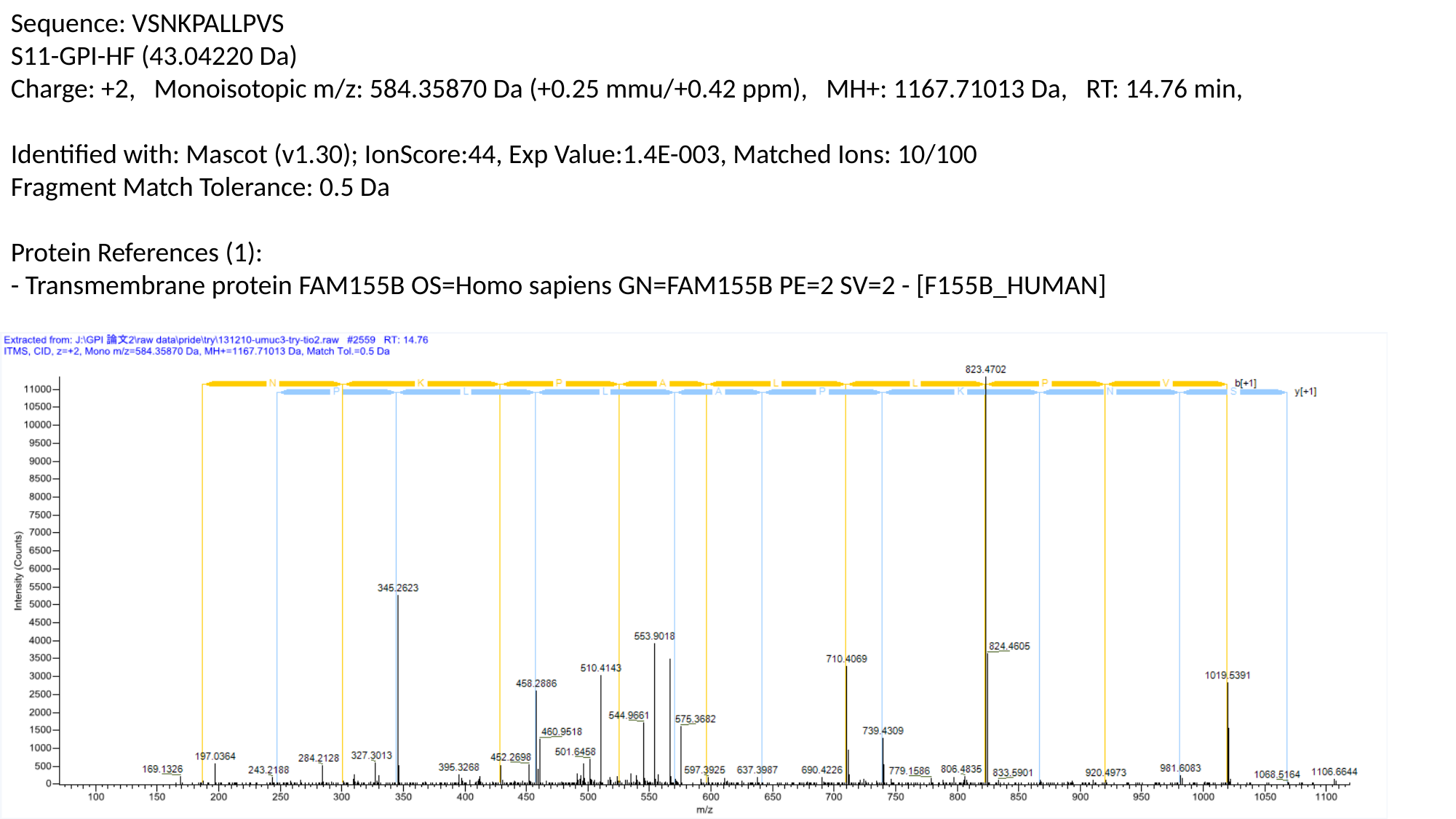

Sequence: VSNKPALLPVS
S11-GPI-HF (43.04220 Da)
Charge: +2, Monoisotopic m/z: 584.35870 Da (+0.25 mmu/+0.42 ppm), MH+: 1167.71013 Da, RT: 14.76 min,
Identified with: Mascot (v1.30); IonScore:44, Exp Value:1.4E-003, Matched Ions: 10/100
Fragment Match Tolerance: 0.5 Da
Protein References (1):
- Transmembrane protein FAM155B OS=Homo sapiens GN=FAM155B PE=2 SV=2 - [F155B_HUMAN]

## Slide 38
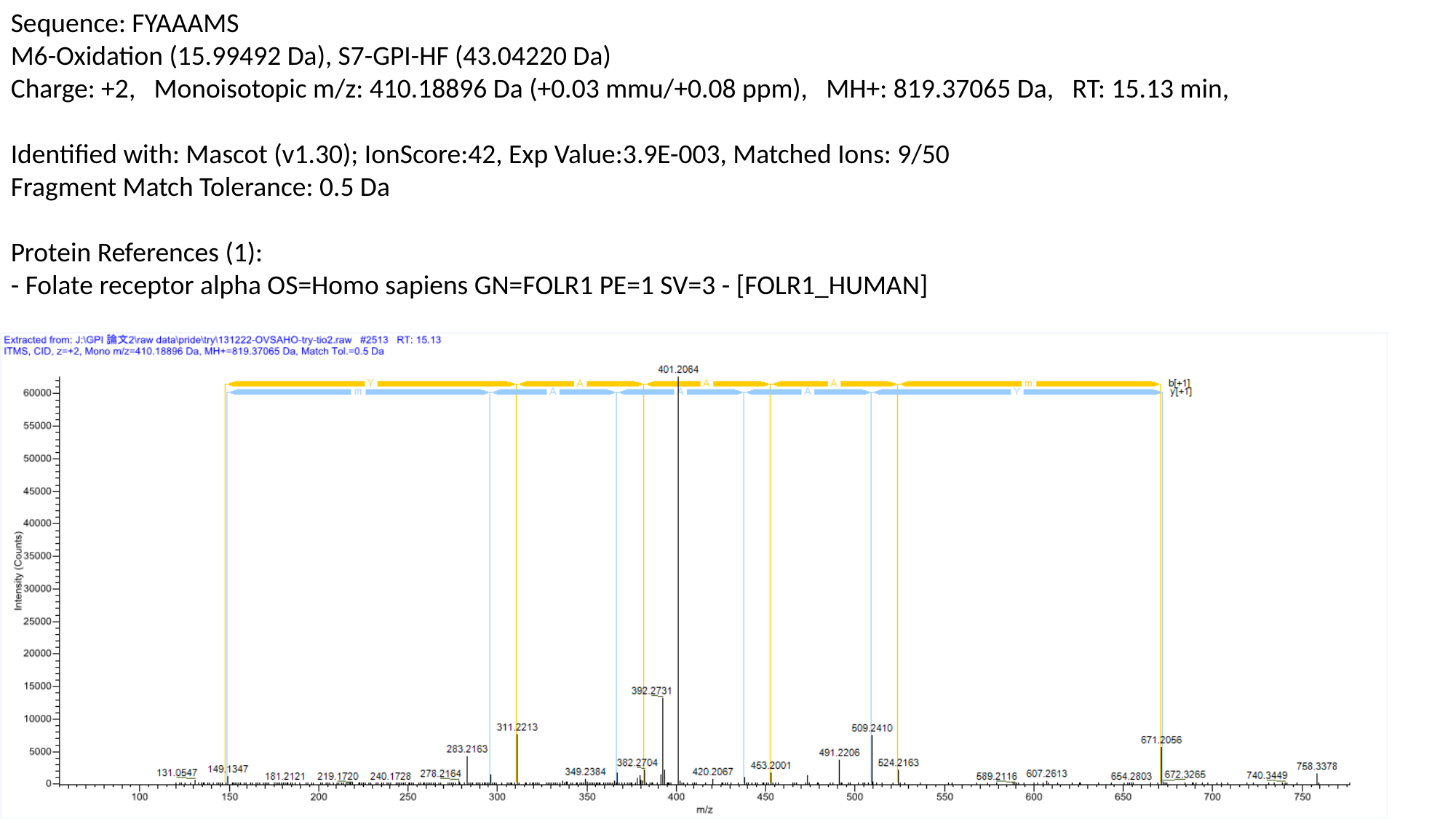

Sequence: FYAAAMS
M6-Oxidation (15.99492 Da), S7-GPI-HF (43.04220 Da)
Charge: +2, Monoisotopic m/z: 410.18896 Da (+0.03 mmu/+0.08 ppm), MH+: 819.37065 Da, RT: 15.13 min,
Identified with: Mascot (v1.30); IonScore:42, Exp Value:3.9E-003, Matched Ions: 9/50
Fragment Match Tolerance: 0.5 Da
Protein References (1):
- Folate receptor alpha OS=Homo sapiens GN=FOLR1 PE=1 SV=3 - [FOLR1_HUMAN]

## Slide 39
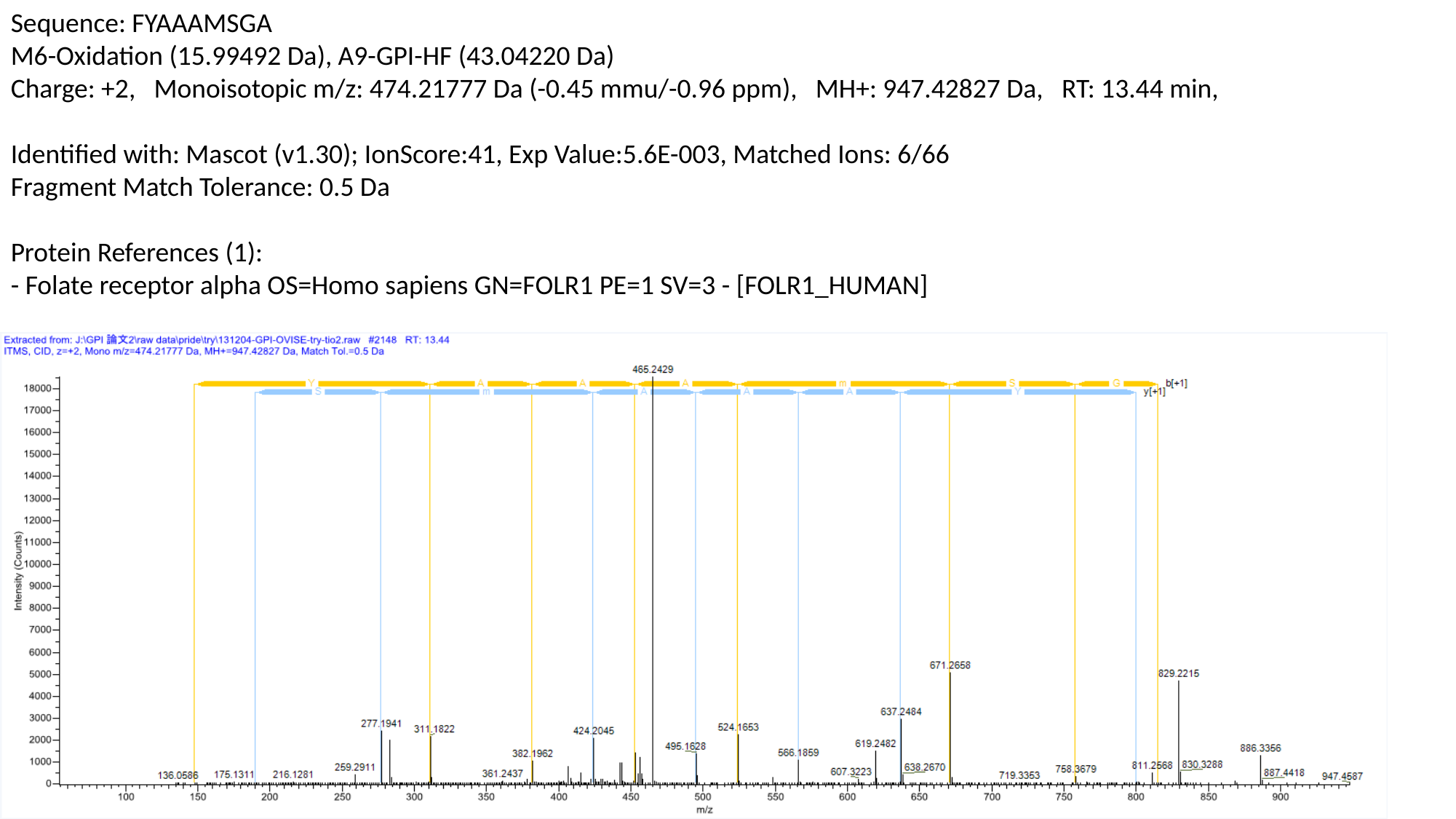

Sequence: FYAAAMSGA
M6-Oxidation (15.99492 Da), A9-GPI-HF (43.04220 Da)
Charge: +2, Monoisotopic m/z: 474.21777 Da (-0.45 mmu/-0.96 ppm), MH+: 947.42827 Da, RT: 13.44 min,
Identified with: Mascot (v1.30); IonScore:41, Exp Value:5.6E-003, Matched Ions: 6/66
Fragment Match Tolerance: 0.5 Da
Protein References (1):
- Folate receptor alpha OS=Homo sapiens GN=FOLR1 PE=1 SV=3 - [FOLR1_HUMAN]

## Slide 40
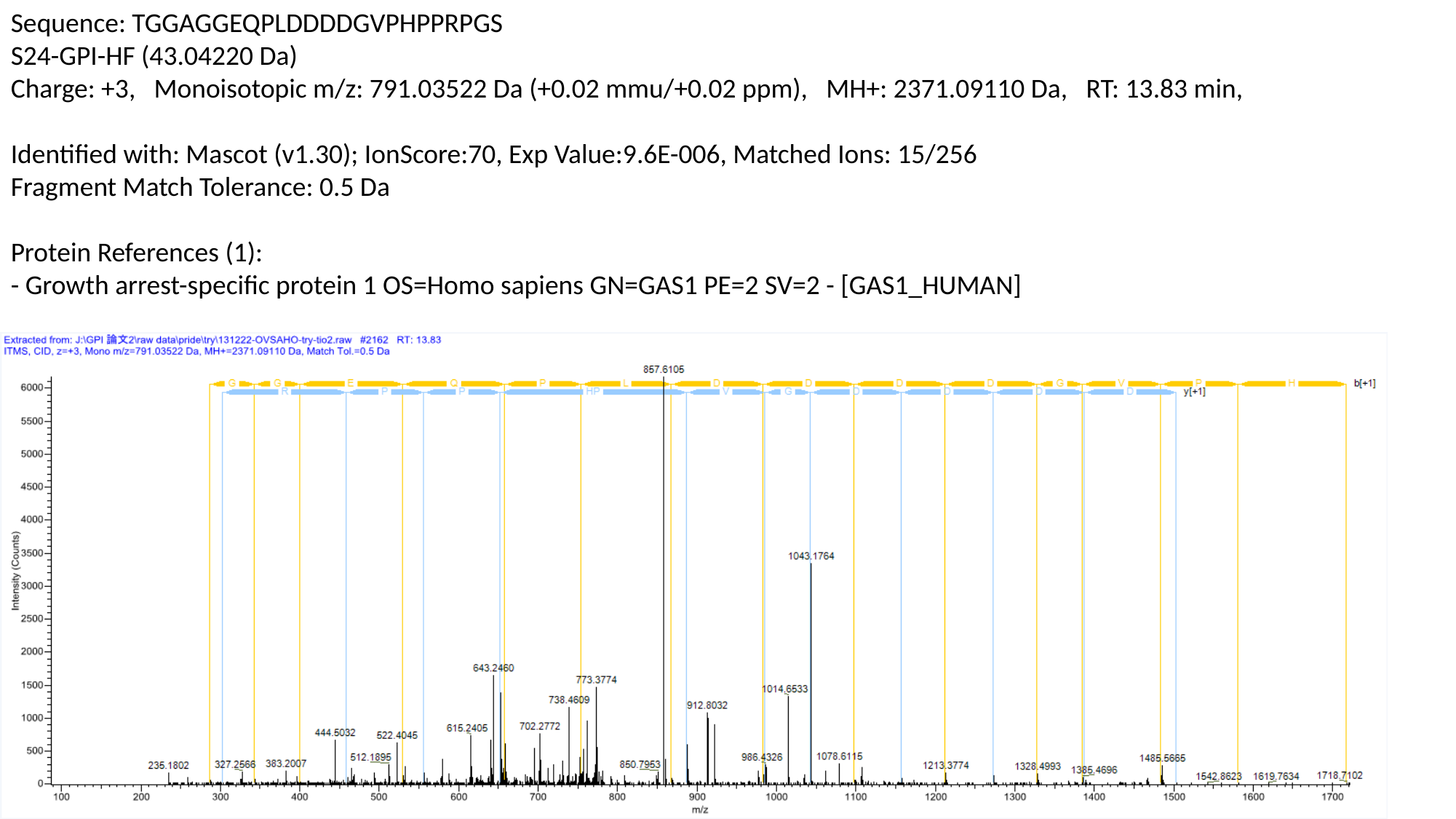

Sequence: TGGAGGEQPLDDDDGVPHPPRPGS
S24-GPI-HF (43.04220 Da)
Charge: +3, Monoisotopic m/z: 791.03522 Da (+0.02 mmu/+0.02 ppm), MH+: 2371.09110 Da, RT: 13.83 min,
Identified with: Mascot (v1.30); IonScore:70, Exp Value:9.6E-006, Matched Ions: 15/256
Fragment Match Tolerance: 0.5 Da
Protein References (1):
- Growth arrest-specific protein 1 OS=Homo sapiens GN=GAS1 PE=2 SV=2 - [GAS1_HUMAN]

## Slide 41
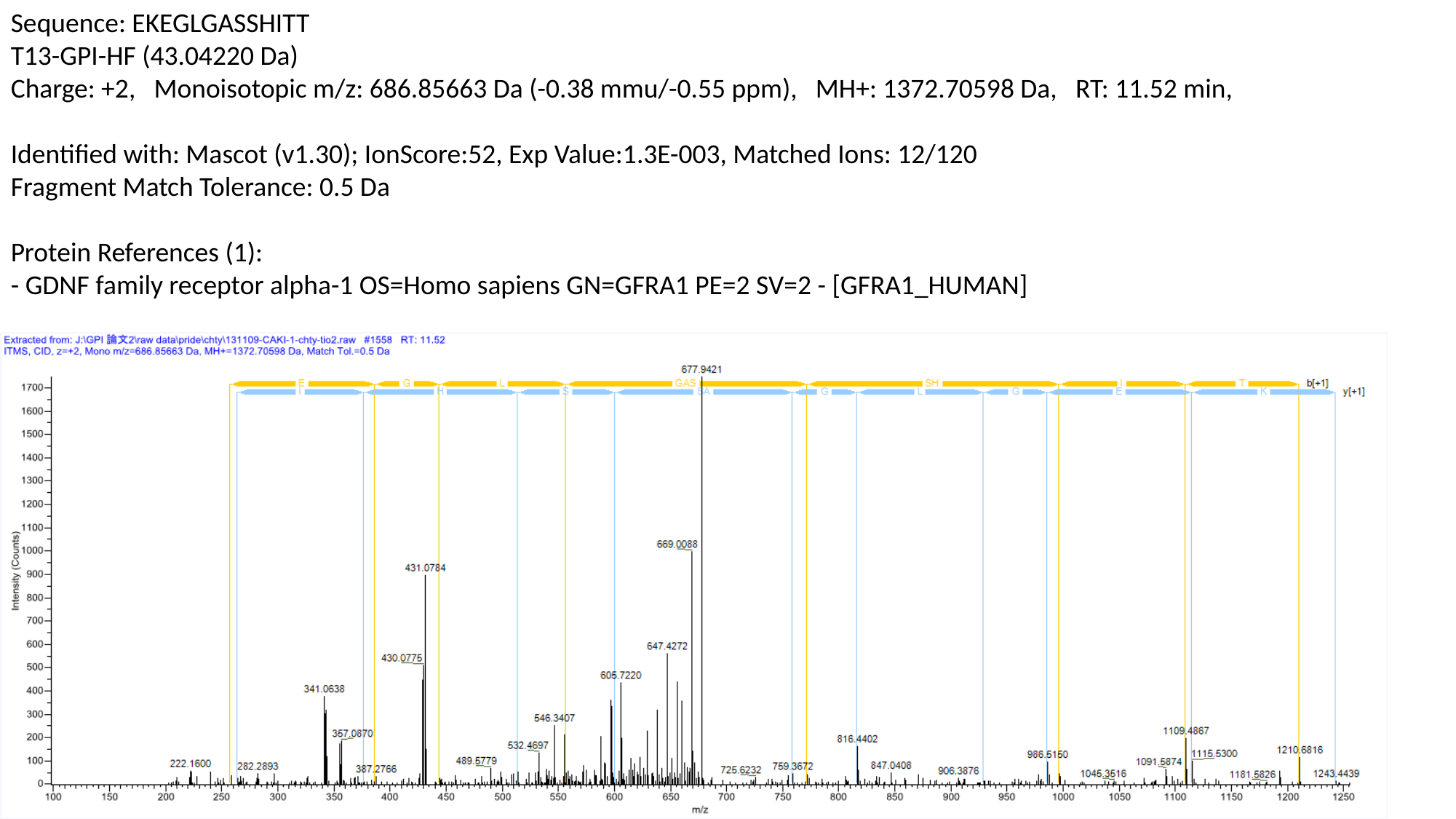

Sequence: EKEGLGASSHITT
T13-GPI-HF (43.04220 Da)
Charge: +2, Monoisotopic m/z: 686.85663 Da (-0.38 mmu/-0.55 ppm), MH+: 1372.70598 Da, RT: 11.52 min,
Identified with: Mascot (v1.30); IonScore:52, Exp Value:1.3E-003, Matched Ions: 12/120
Fragment Match Tolerance: 0.5 Da
Protein References (1):
- GDNF family receptor alpha-1 OS=Homo sapiens GN=GFRA1 PE=2 SV=2 - [GFRA1_HUMAN]

## Slide 42
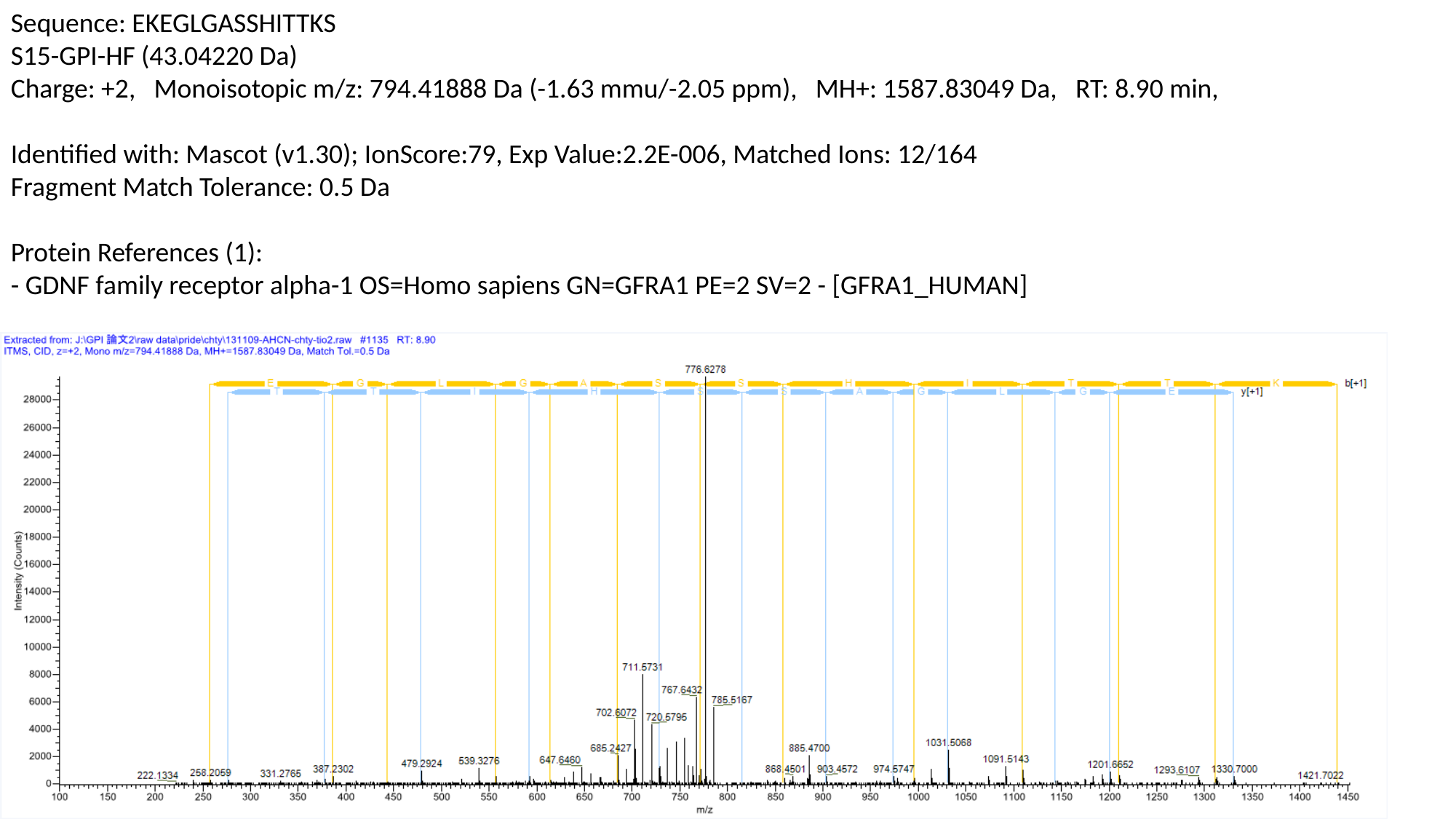

Sequence: EKEGLGASSHITTKS
S15-GPI-HF (43.04220 Da)
Charge: +2, Monoisotopic m/z: 794.41888 Da (-1.63 mmu/-2.05 ppm), MH+: 1587.83049 Da, RT: 8.90 min,
Identified with: Mascot (v1.30); IonScore:79, Exp Value:2.2E-006, Matched Ions: 12/164
Fragment Match Tolerance: 0.5 Da
Protein References (1):
- GDNF family receptor alpha-1 OS=Homo sapiens GN=GFRA1 PE=2 SV=2 - [GFRA1_HUMAN]

## Slide 43
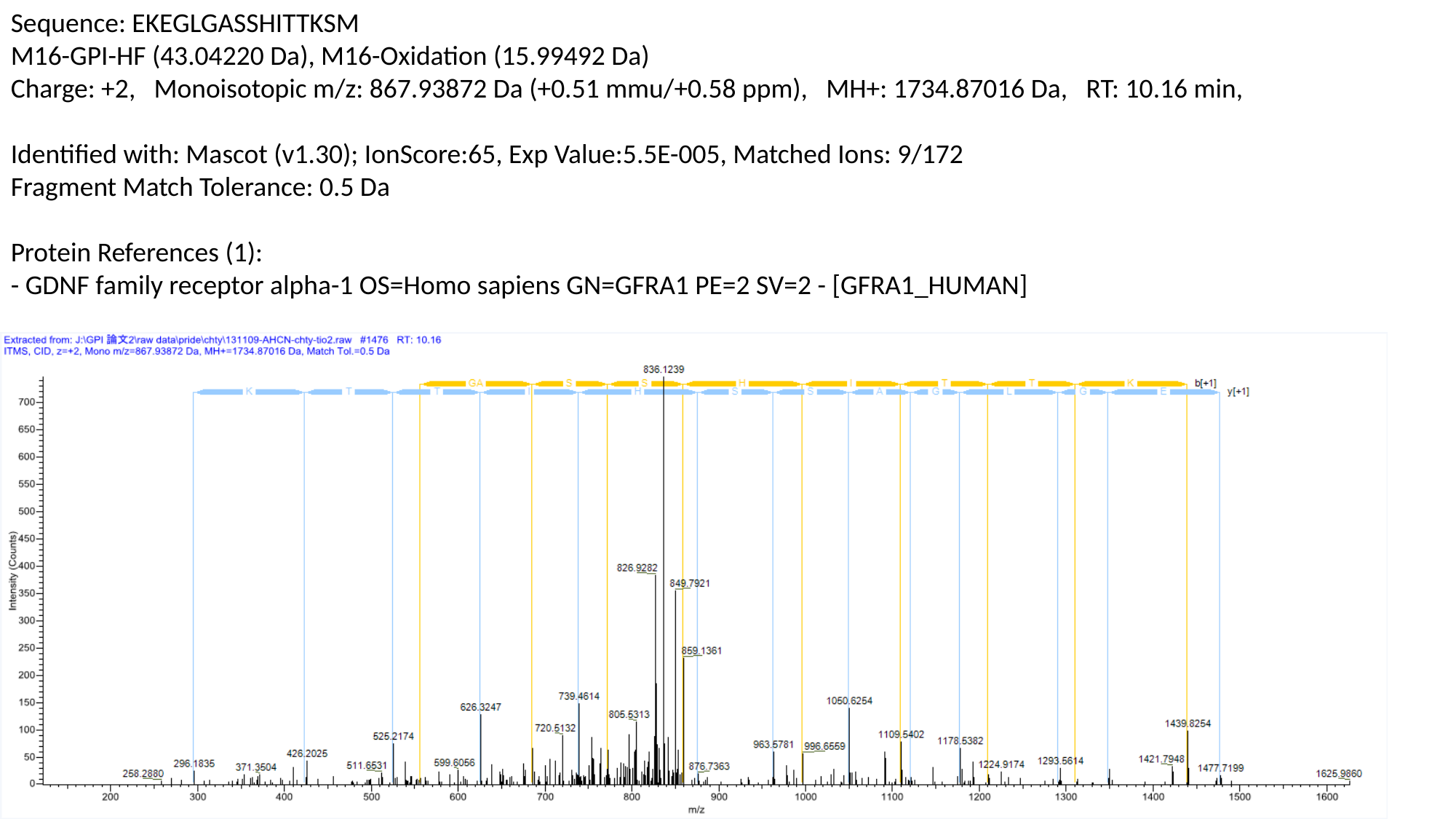

Sequence: EKEGLGASSHITTKSM
M16-GPI-HF (43.04220 Da), M16-Oxidation (15.99492 Da)
Charge: +2, Monoisotopic m/z: 867.93872 Da (+0.51 mmu/+0.58 ppm), MH+: 1734.87016 Da, RT: 10.16 min,
Identified with: Mascot (v1.30); IonScore:65, Exp Value:5.5E-005, Matched Ions: 9/172
Fragment Match Tolerance: 0.5 Da
Protein References (1):
- GDNF family receptor alpha-1 OS=Homo sapiens GN=GFRA1 PE=2 SV=2 - [GFRA1_HUMAN]

## Slide 44
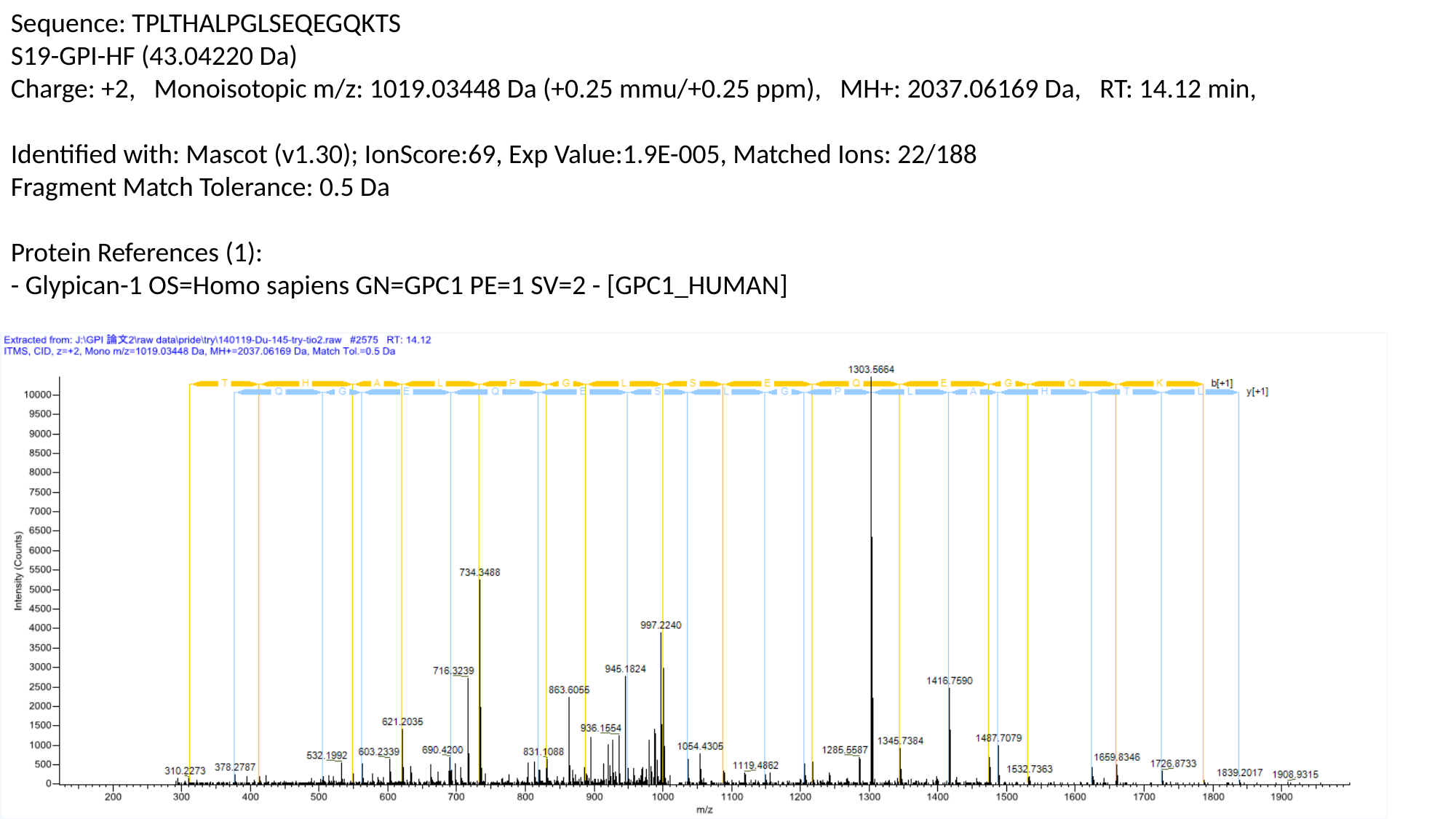

Sequence: TPLTHALPGLSEQEGQKTS
S19-GPI-HF (43.04220 Da)
Charge: +2, Monoisotopic m/z: 1019.03448 Da (+0.25 mmu/+0.25 ppm), MH+: 2037.06169 Da, RT: 14.12 min,
Identified with: Mascot (v1.30); IonScore:69, Exp Value:1.9E-005, Matched Ions: 22/188
Fragment Match Tolerance: 0.5 Da
Protein References (1):
- Glypican-1 OS=Homo sapiens GN=GPC1 PE=1 SV=2 - [GPC1_HUMAN]

## Slide 45
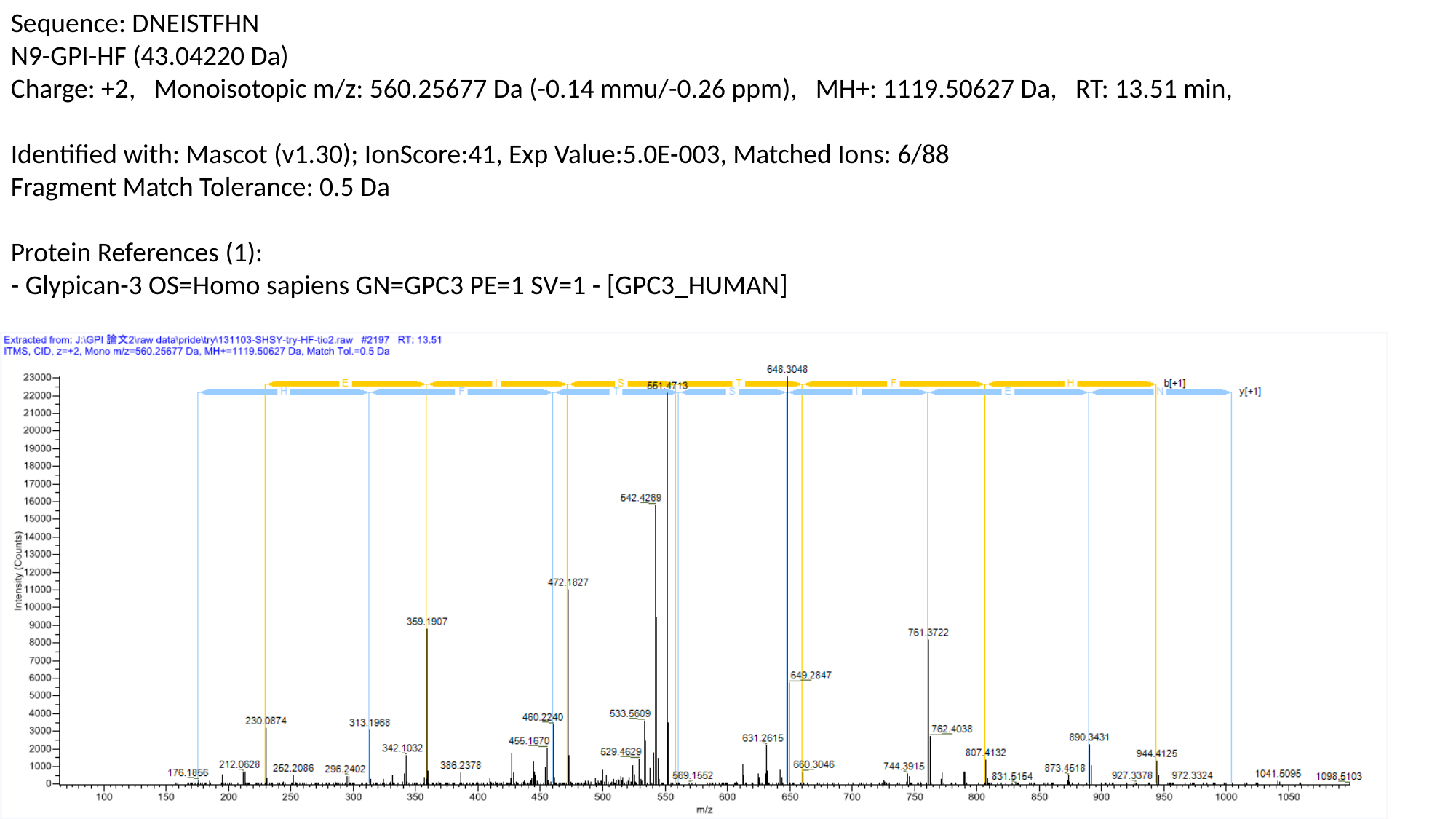

Sequence: DNEISTFHN
N9-GPI-HF (43.04220 Da)
Charge: +2, Monoisotopic m/z: 560.25677 Da (-0.14 mmu/-0.26 ppm), MH+: 1119.50627 Da, RT: 13.51 min,
Identified with: Mascot (v1.30); IonScore:41, Exp Value:5.0E-003, Matched Ions: 6/88
Fragment Match Tolerance: 0.5 Da
Protein References (1):
- Glypican-3 OS=Homo sapiens GN=GPC3 PE=1 SV=1 - [GPC3_HUMAN]

## Slide 46
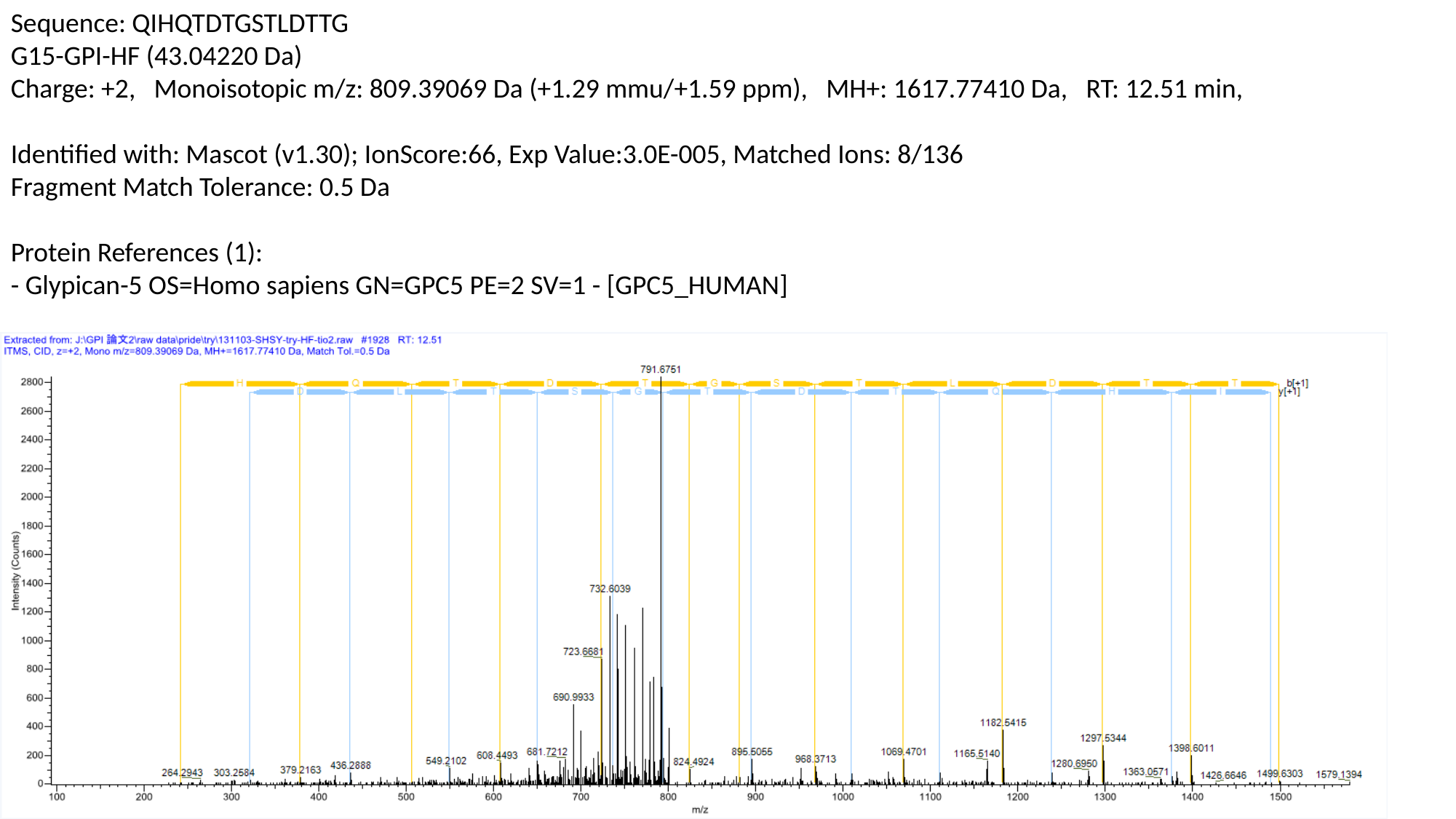

Sequence: QIHQTDTGSTLDTTG
G15-GPI-HF (43.04220 Da)
Charge: +2, Monoisotopic m/z: 809.39069 Da (+1.29 mmu/+1.59 ppm), MH+: 1617.77410 Da, RT: 12.51 min,
Identified with: Mascot (v1.30); IonScore:66, Exp Value:3.0E-005, Matched Ions: 8/136
Fragment Match Tolerance: 0.5 Da
Protein References (1):
- Glypican-5 OS=Homo sapiens GN=GPC5 PE=2 SV=1 - [GPC5_HUMAN]

## Slide 47
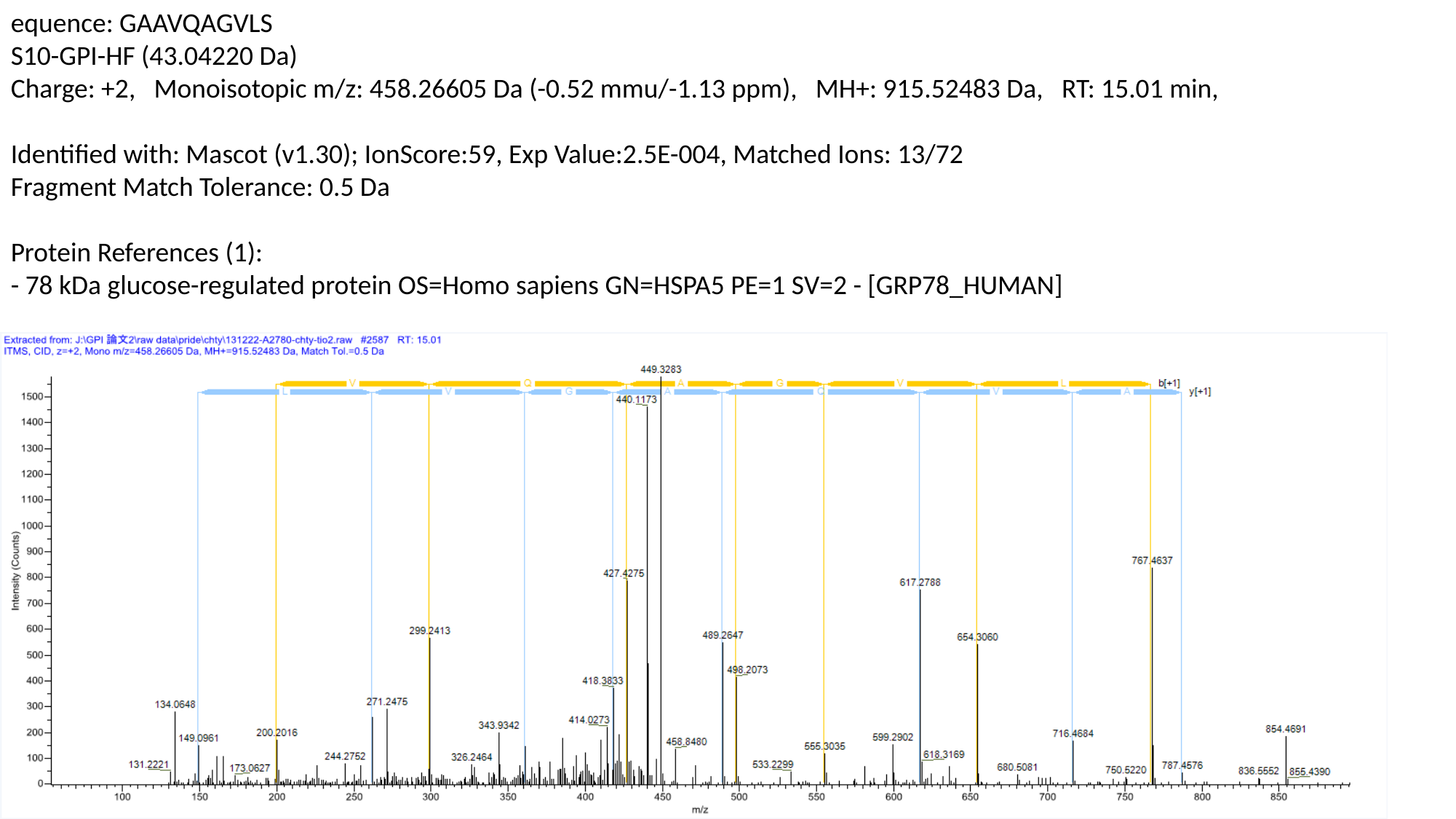

equence: GAAVQAGVLS
S10-GPI-HF (43.04220 Da)
Charge: +2, Monoisotopic m/z: 458.26605 Da (-0.52 mmu/-1.13 ppm), MH+: 915.52483 Da, RT: 15.01 min,
Identified with: Mascot (v1.30); IonScore:59, Exp Value:2.5E-004, Matched Ions: 13/72
Fragment Match Tolerance: 0.5 Da
Protein References (1):
- 78 kDa glucose-regulated protein OS=Homo sapiens GN=HSPA5 PE=1 SV=2 - [GRP78_HUMAN]

## Slide 48
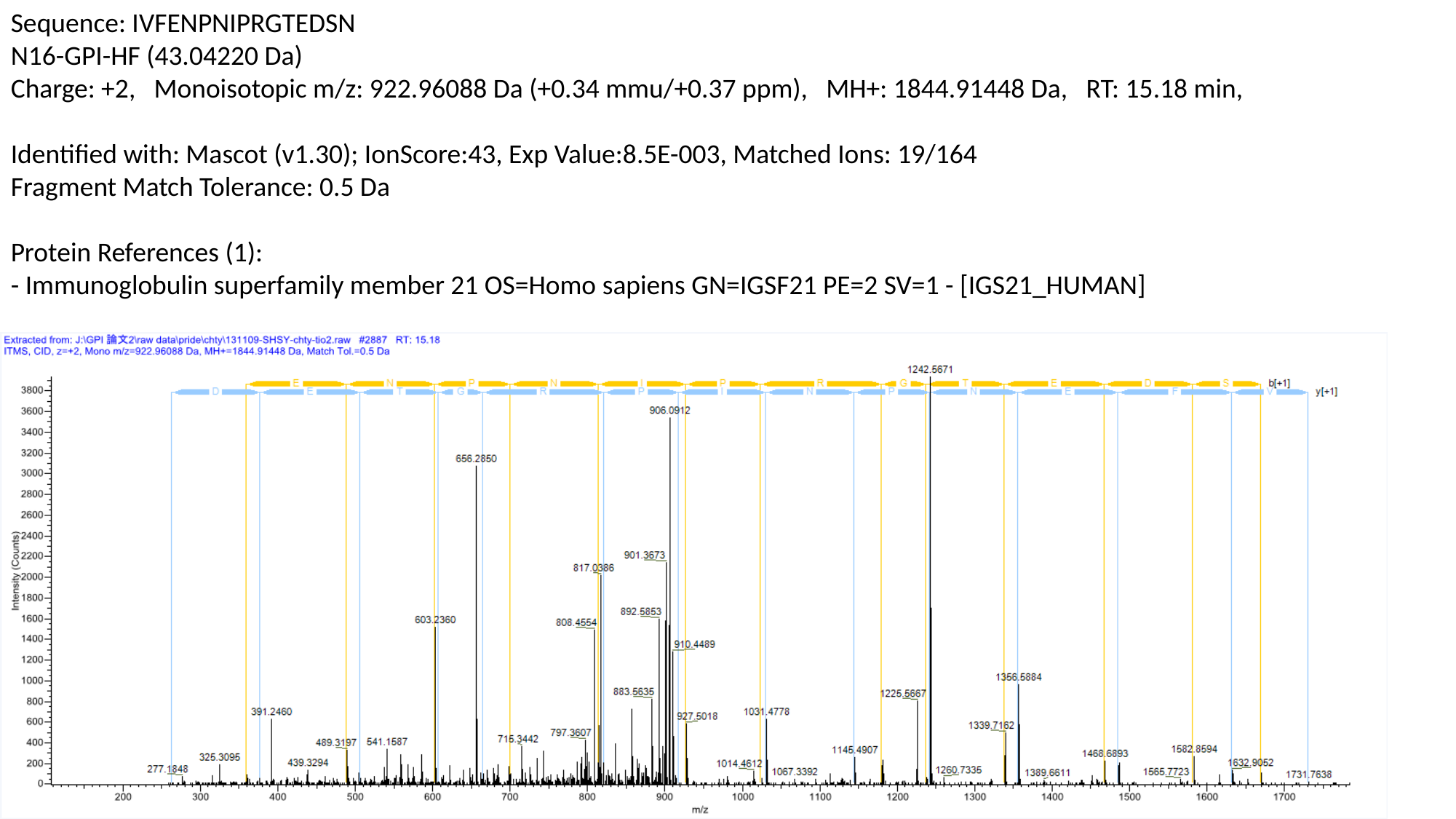

Sequence: IVFENPNIPRGTEDSN
N16-GPI-HF (43.04220 Da)
Charge: +2, Monoisotopic m/z: 922.96088 Da (+0.34 mmu/+0.37 ppm), MH+: 1844.91448 Da, RT: 15.18 min,
Identified with: Mascot (v1.30); IonScore:43, Exp Value:8.5E-003, Matched Ions: 19/164
Fragment Match Tolerance: 0.5 Da
Protein References (1):
- Immunoglobulin superfamily member 21 OS=Homo sapiens GN=IGSF21 PE=2 SV=1 - [IGS21_HUMAN]

## Slide 49
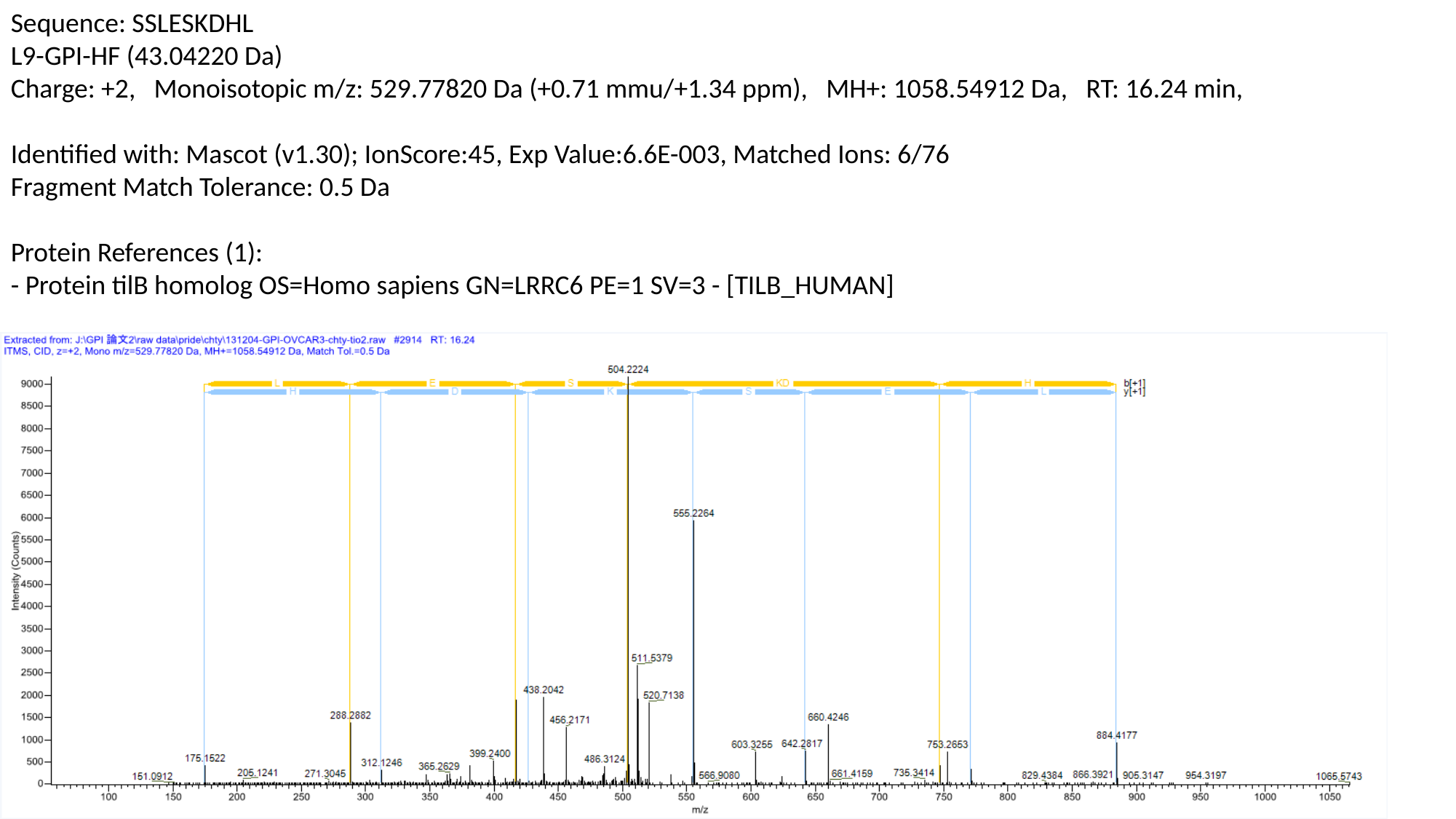

Sequence: SSLESKDHL
L9-GPI-HF (43.04220 Da)
Charge: +2, Monoisotopic m/z: 529.77820 Da (+0.71 mmu/+1.34 ppm), MH+: 1058.54912 Da, RT: 16.24 min,
Identified with: Mascot (v1.30); IonScore:45, Exp Value:6.6E-003, Matched Ions: 6/76
Fragment Match Tolerance: 0.5 Da
Protein References (1):
- Protein tilB homolog OS=Homo sapiens GN=LRRC6 PE=1 SV=3 - [TILB_HUMAN]

## Slide 50
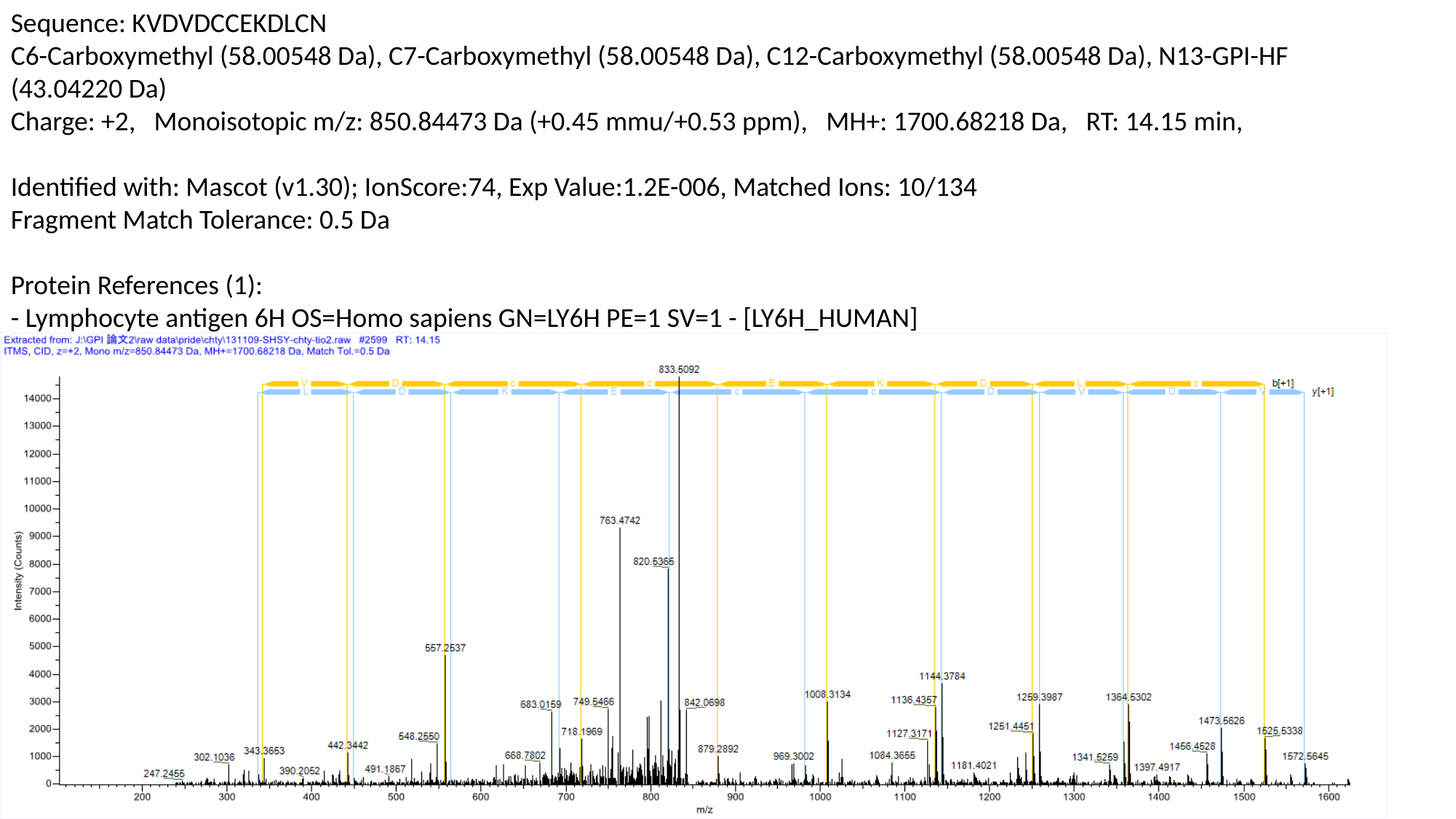

Sequence: KVDVDCCEKDLCN
C6-Carboxymethyl (58.00548 Da), C7-Carboxymethyl (58.00548 Da), C12-Carboxymethyl (58.00548 Da), N13-GPI-HF (43.04220 Da)
Charge: +2, Monoisotopic m/z: 850.84473 Da (+0.45 mmu/+0.53 ppm), MH+: 1700.68218 Da, RT: 14.15 min,
Identified with: Mascot (v1.30); IonScore:74, Exp Value:1.2E-006, Matched Ions: 10/134
Fragment Match Tolerance: 0.5 Da
Protein References (1):
- Lymphocyte antigen 6H OS=Homo sapiens GN=LY6H PE=1 SV=1 - [LY6H_HUMAN]

## Slide 51
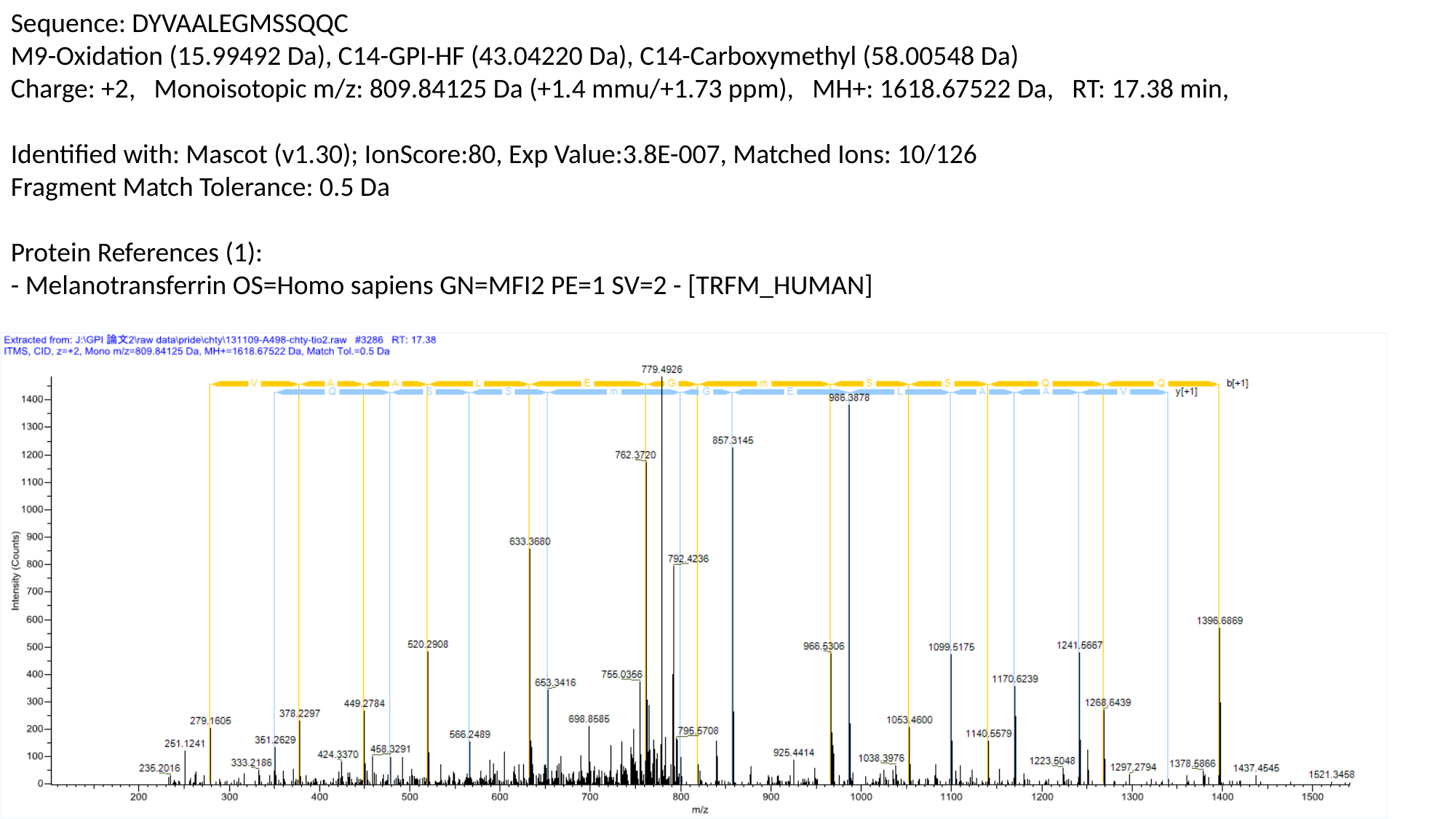

Sequence: DYVAALEGMSSQQC
M9-Oxidation (15.99492 Da), C14-GPI-HF (43.04220 Da), C14-Carboxymethyl (58.00548 Da)
Charge: +2, Monoisotopic m/z: 809.84125 Da (+1.4 mmu/+1.73 ppm), MH+: 1618.67522 Da, RT: 17.38 min,
Identified with: Mascot (v1.30); IonScore:80, Exp Value:3.8E-007, Matched Ions: 10/126
Fragment Match Tolerance: 0.5 Da
Protein References (1):
- Melanotransferrin OS=Homo sapiens GN=MFI2 PE=1 SV=2 - [TRFM_HUMAN]

## Slide 52
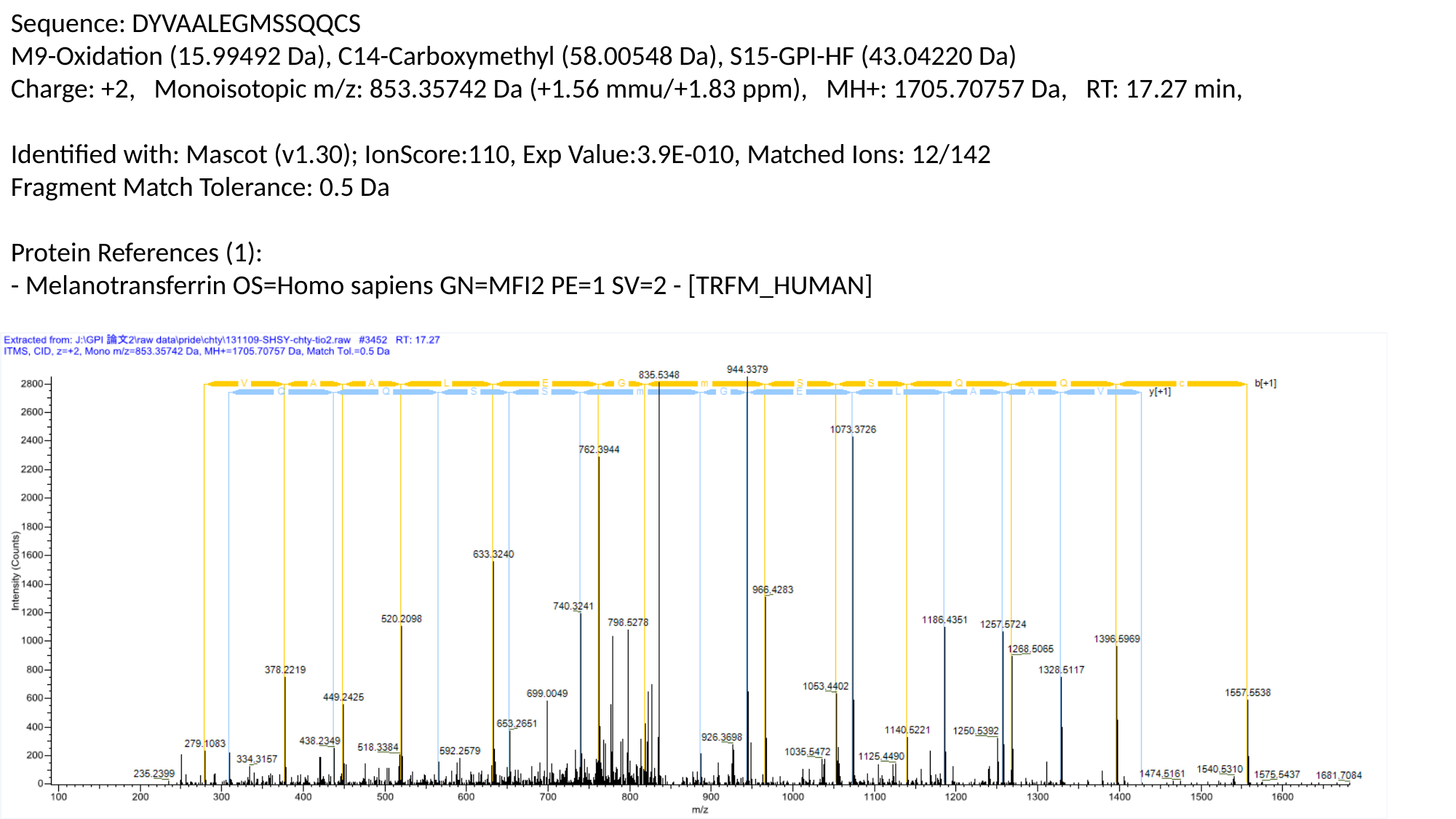

Sequence: DYVAALEGMSSQQCS
M9-Oxidation (15.99492 Da), C14-Carboxymethyl (58.00548 Da), S15-GPI-HF (43.04220 Da)
Charge: +2, Monoisotopic m/z: 853.35742 Da (+1.56 mmu/+1.83 ppm), MH+: 1705.70757 Da, RT: 17.27 min,
Identified with: Mascot (v1.30); IonScore:110, Exp Value:3.9E-010, Matched Ions: 12/142
Fragment Match Tolerance: 0.5 Da
Protein References (1):
- Melanotransferrin OS=Homo sapiens GN=MFI2 PE=1 SV=2 - [TRFM_HUMAN]

## Slide 53
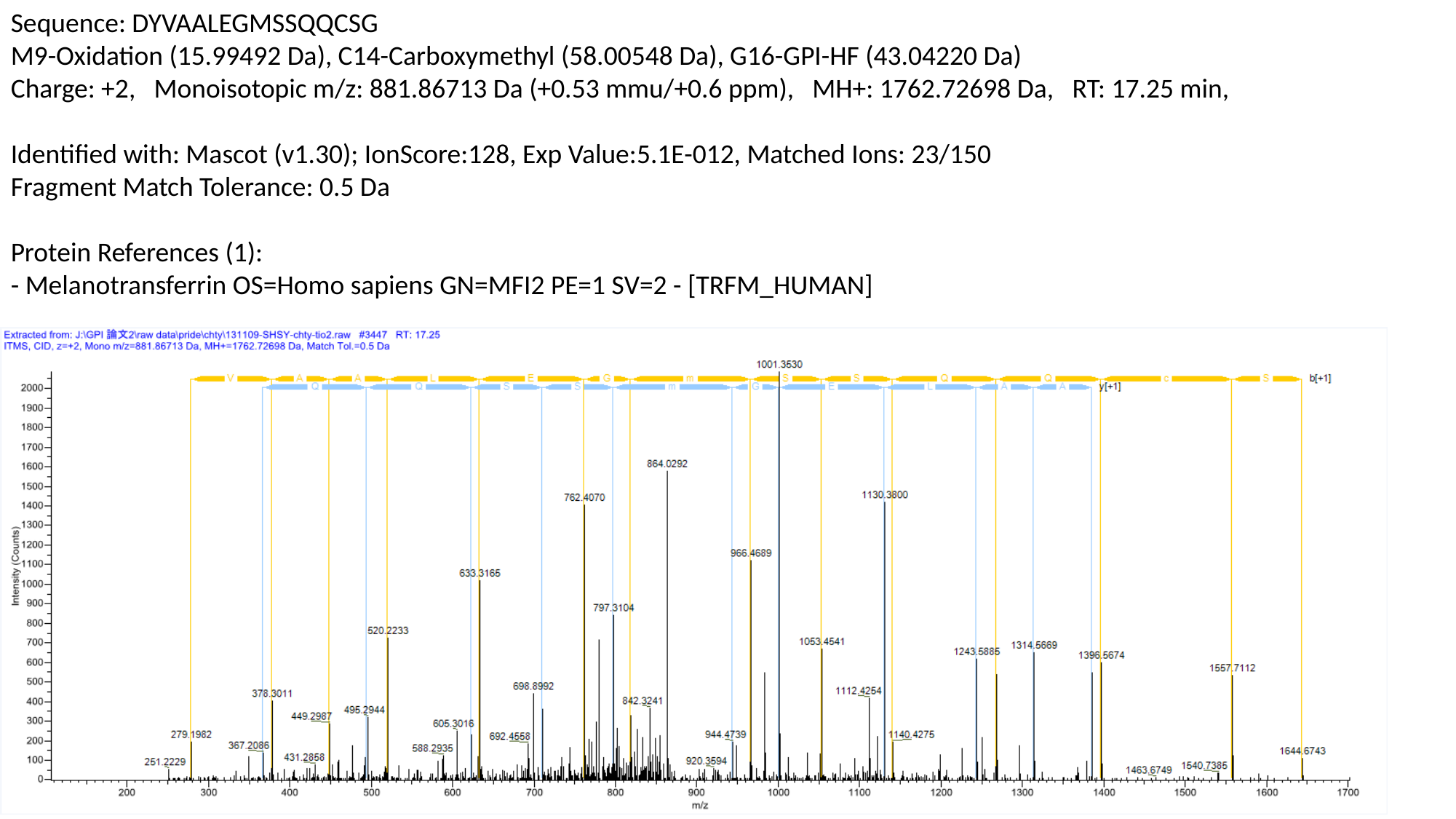

Sequence: DYVAALEGMSSQQCSG
M9-Oxidation (15.99492 Da), C14-Carboxymethyl (58.00548 Da), G16-GPI-HF (43.04220 Da)
Charge: +2, Monoisotopic m/z: 881.86713 Da (+0.53 mmu/+0.6 ppm), MH+: 1762.72698 Da, RT: 17.25 min,
Identified with: Mascot (v1.30); IonScore:128, Exp Value:5.1E-012, Matched Ions: 23/150
Fragment Match Tolerance: 0.5 Da
Protein References (1):
- Melanotransferrin OS=Homo sapiens GN=MFI2 PE=1 SV=2 - [TRFM_HUMAN]

## Slide 54
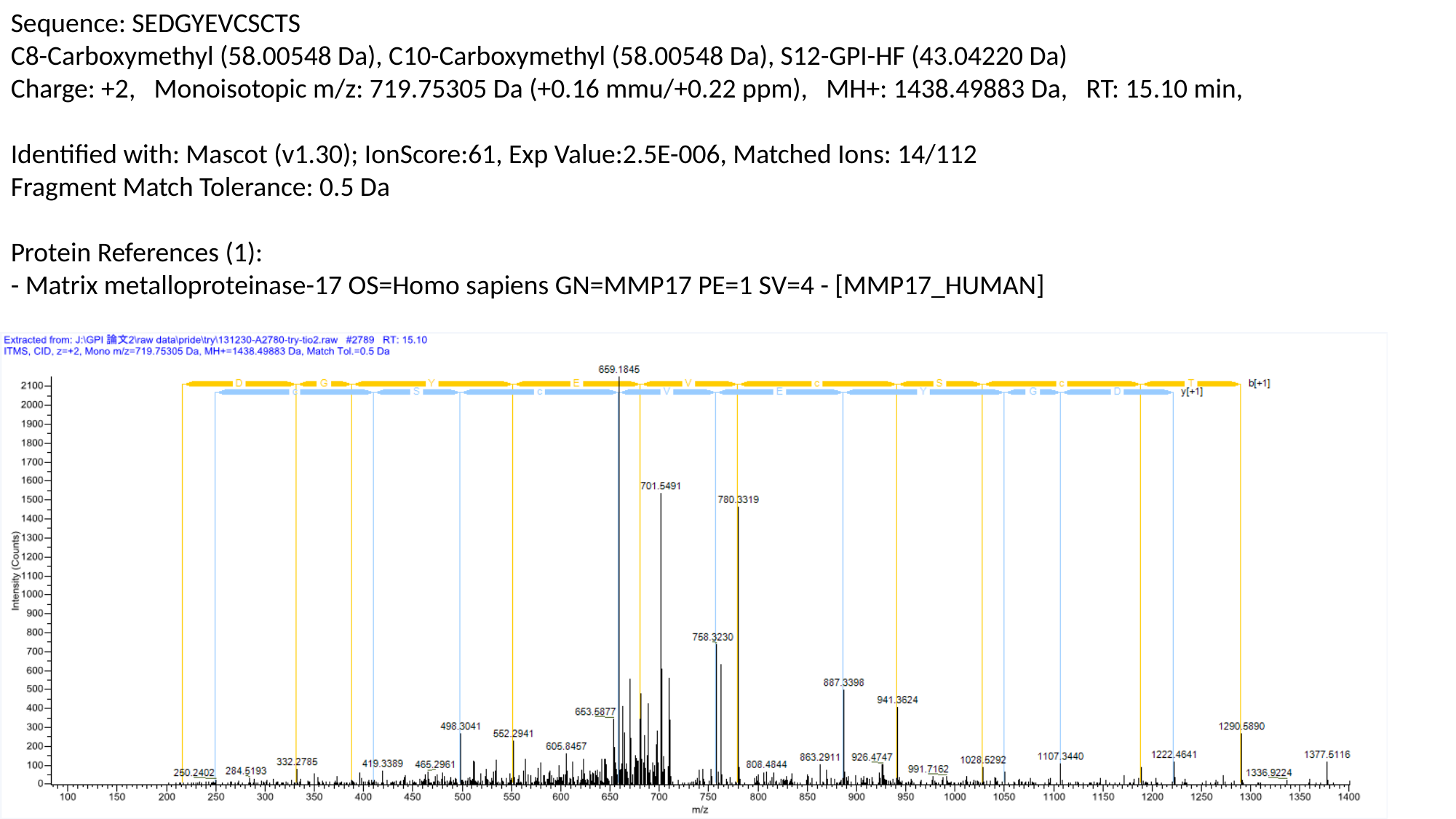

Sequence: SEDGYEVCSCTS
C8-Carboxymethyl (58.00548 Da), C10-Carboxymethyl (58.00548 Da), S12-GPI-HF (43.04220 Da)
Charge: +2, Monoisotopic m/z: 719.75305 Da (+0.16 mmu/+0.22 ppm), MH+: 1438.49883 Da, RT: 15.10 min,
Identified with: Mascot (v1.30); IonScore:61, Exp Value:2.5E-006, Matched Ions: 14/112
Fragment Match Tolerance: 0.5 Da
Protein References (1):
- Matrix metalloproteinase-17 OS=Homo sapiens GN=MMP17 PE=1 SV=4 - [MMP17_HUMAN]

## Slide 55
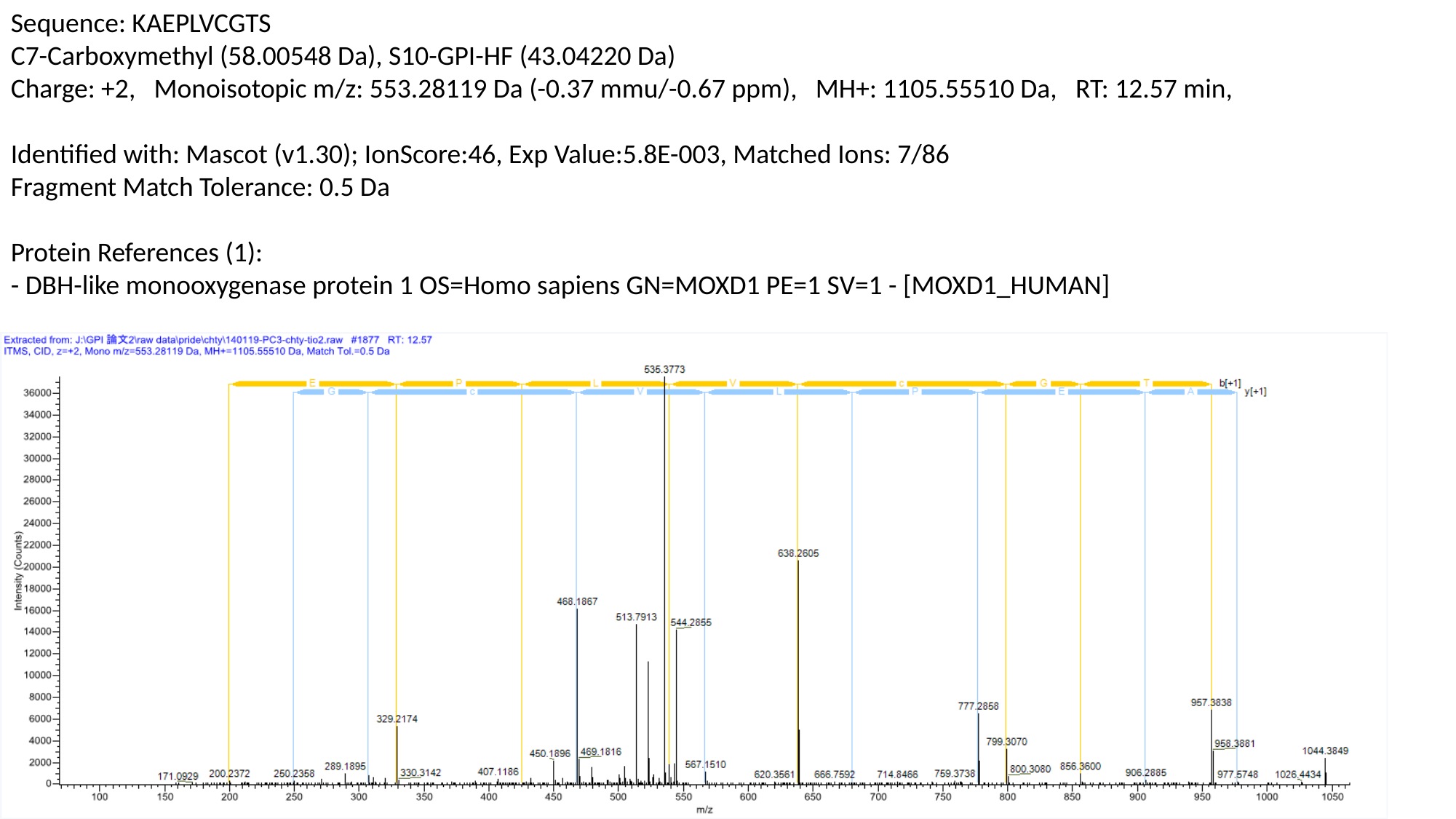

Sequence: KAEPLVCGTS
C7-Carboxymethyl (58.00548 Da), S10-GPI-HF (43.04220 Da)
Charge: +2, Monoisotopic m/z: 553.28119 Da (-0.37 mmu/-0.67 ppm), MH+: 1105.55510 Da, RT: 12.57 min,
Identified with: Mascot (v1.30); IonScore:46, Exp Value:5.8E-003, Matched Ions: 7/86
Fragment Match Tolerance: 0.5 Da
Protein References (1):
- DBH-like monooxygenase protein 1 OS=Homo sapiens GN=MOXD1 PE=1 SV=1 - [MOXD1_HUMAN]

## Slide 56
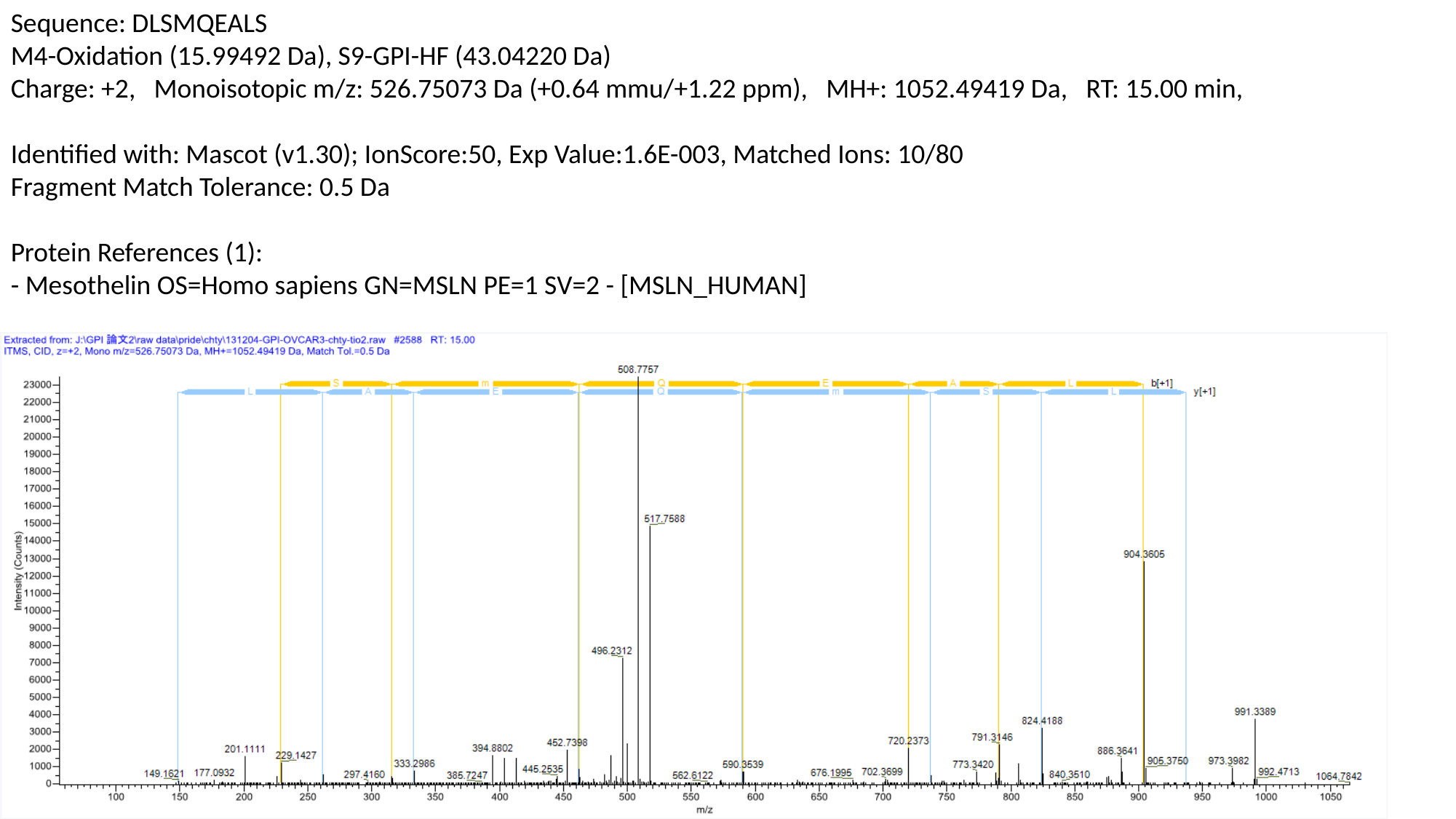

Sequence: DLSMQEALS
M4-Oxidation (15.99492 Da), S9-GPI-HF (43.04220 Da)
Charge: +2, Monoisotopic m/z: 526.75073 Da (+0.64 mmu/+1.22 ppm), MH+: 1052.49419 Da, RT: 15.00 min,
Identified with: Mascot (v1.30); IonScore:50, Exp Value:1.6E-003, Matched Ions: 10/80
Fragment Match Tolerance: 0.5 Da
Protein References (1):
- Mesothelin OS=Homo sapiens GN=MSLN PE=1 SV=2 - [MSLN_HUMAN]

## Slide 57
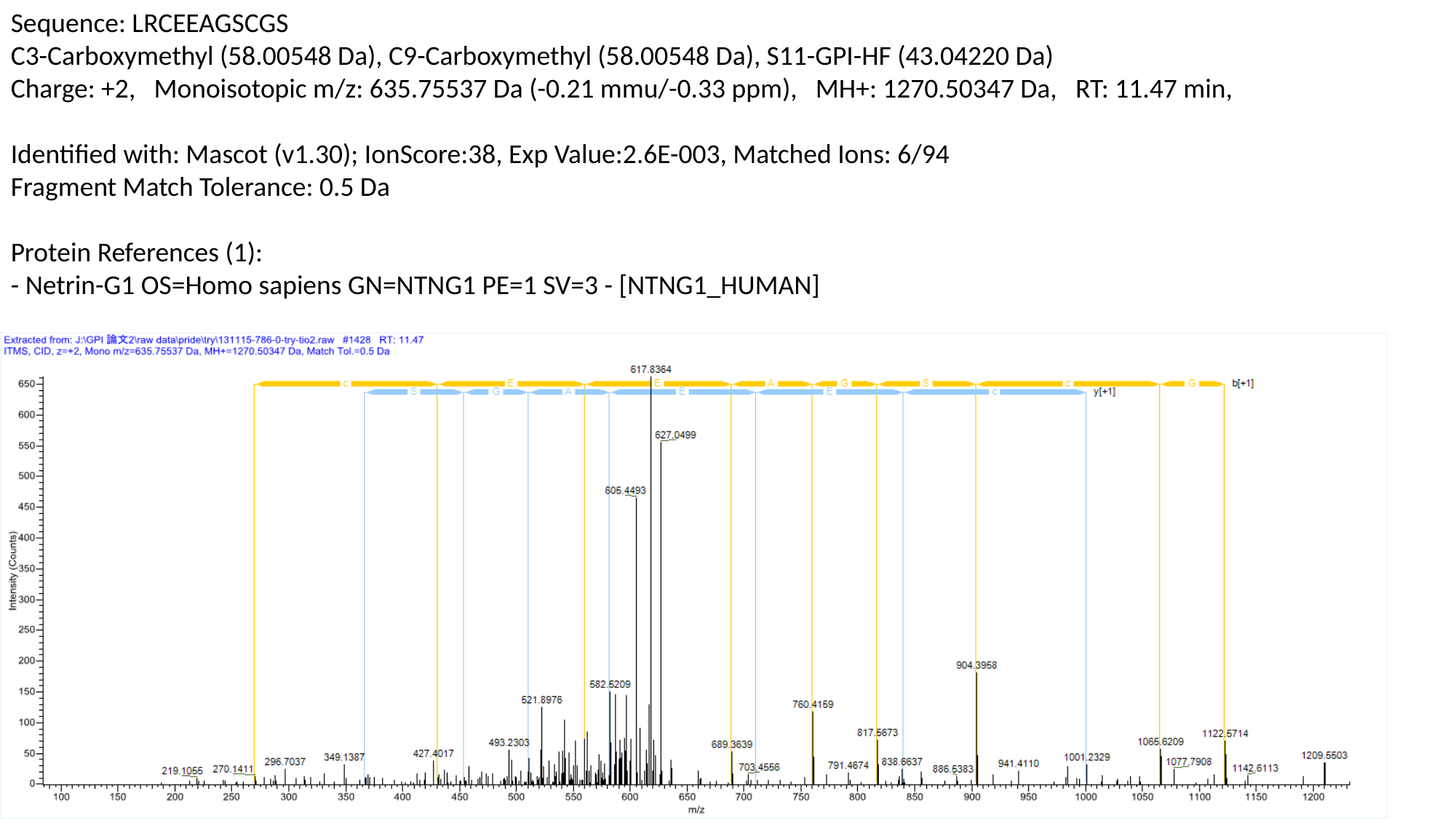

Sequence: LRCEEAGSCGS
C3-Carboxymethyl (58.00548 Da), C9-Carboxymethyl (58.00548 Da), S11-GPI-HF (43.04220 Da)
Charge: +2, Monoisotopic m/z: 635.75537 Da (-0.21 mmu/-0.33 ppm), MH+: 1270.50347 Da, RT: 11.47 min,
Identified with: Mascot (v1.30); IonScore:38, Exp Value:2.6E-003, Matched Ions: 6/94
Fragment Match Tolerance: 0.5 Da
Protein References (1):
- Netrin-G1 OS=Homo sapiens GN=NTNG1 PE=1 SV=3 - [NTNG1_HUMAN]

## Slide 58
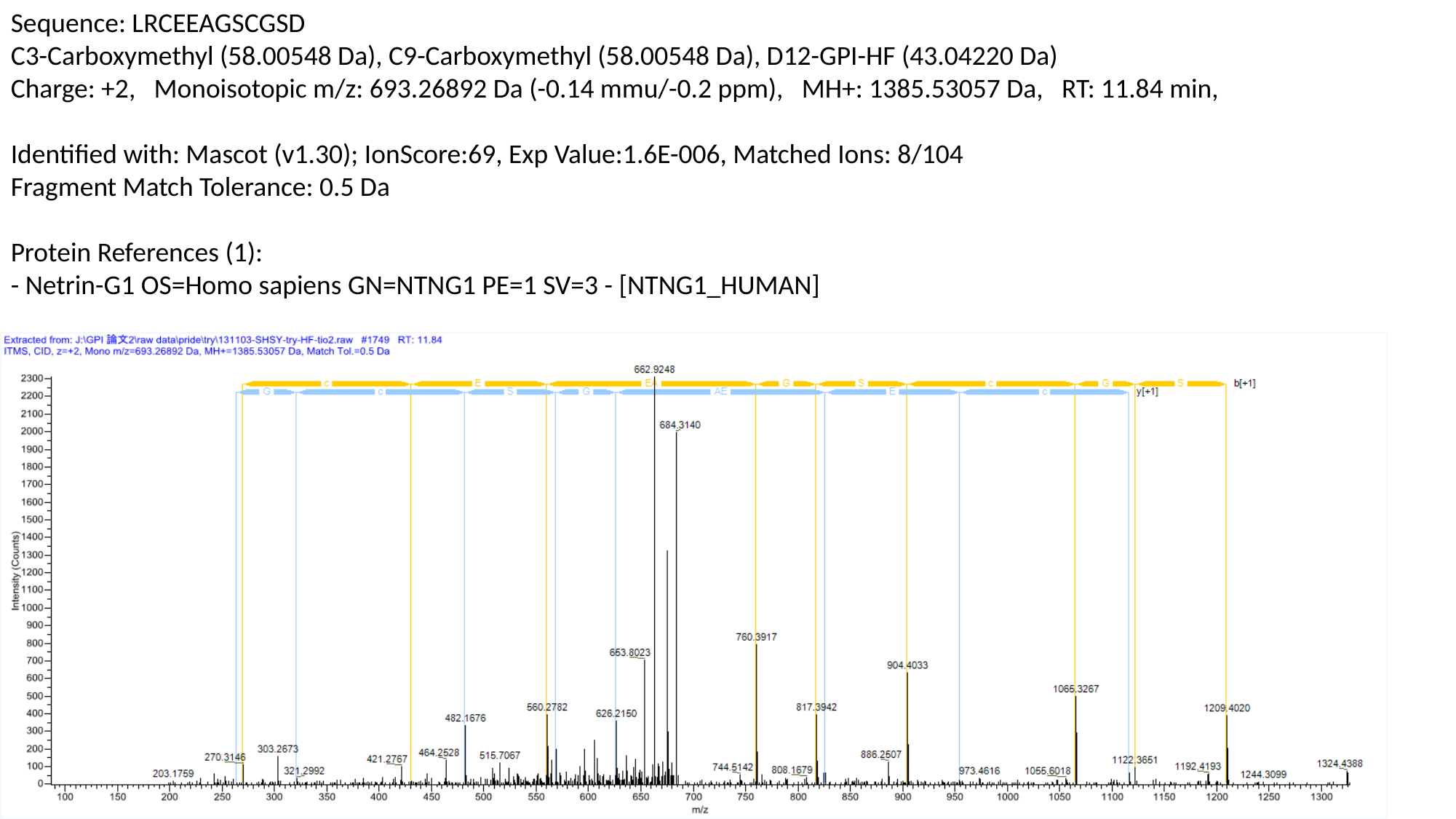

Sequence: LRCEEAGSCGSD
C3-Carboxymethyl (58.00548 Da), C9-Carboxymethyl (58.00548 Da), D12-GPI-HF (43.04220 Da)
Charge: +2, Monoisotopic m/z: 693.26892 Da (-0.14 mmu/-0.2 ppm), MH+: 1385.53057 Da, RT: 11.84 min,
Identified with: Mascot (v1.30); IonScore:69, Exp Value:1.6E-006, Matched Ions: 8/104
Fragment Match Tolerance: 0.5 Da
Protein References (1):
- Netrin-G1 OS=Homo sapiens GN=NTNG1 PE=1 SV=3 - [NTNG1_HUMAN]

## Slide 59
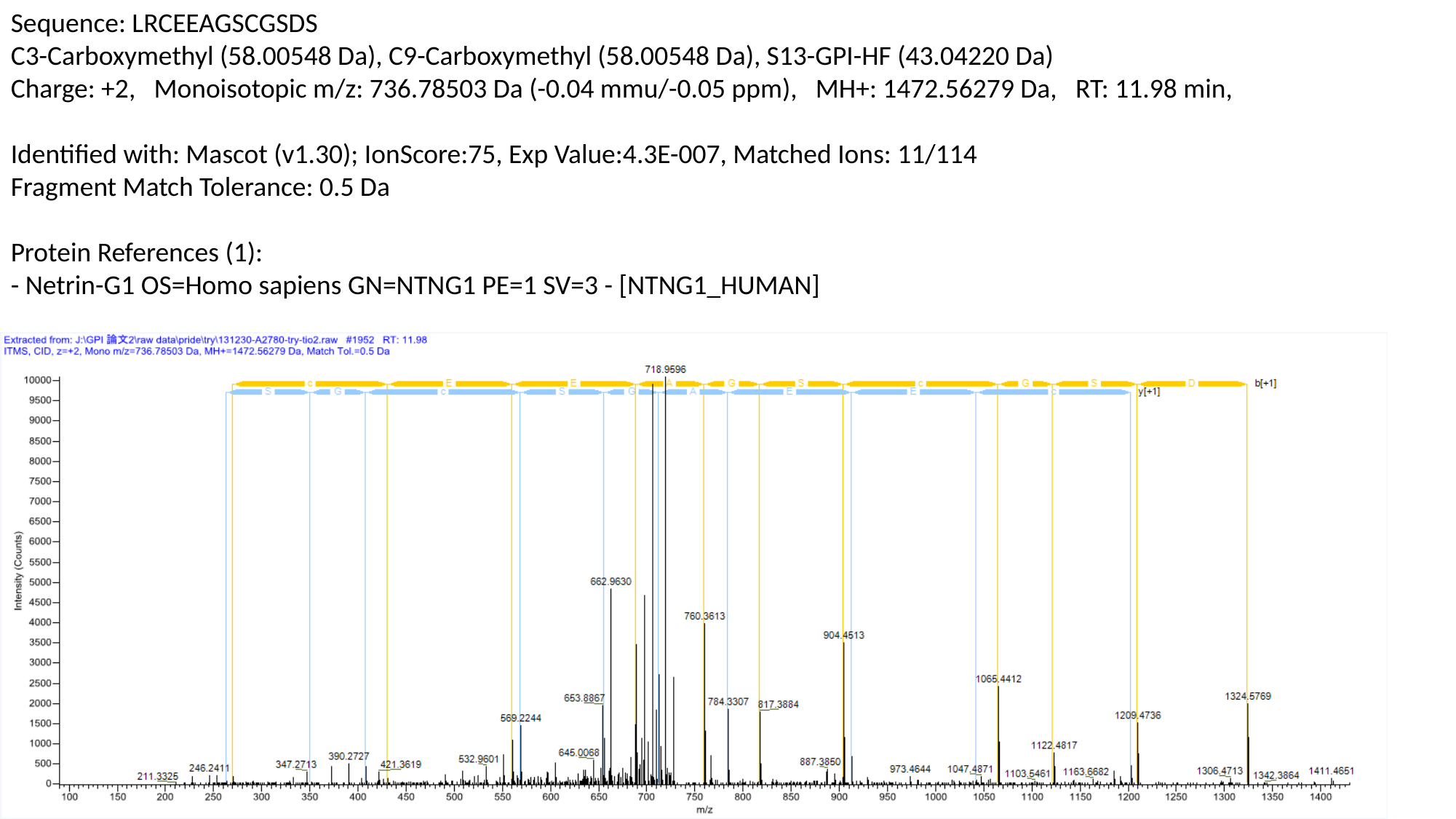

Sequence: LRCEEAGSCGSDS
C3-Carboxymethyl (58.00548 Da), C9-Carboxymethyl (58.00548 Da), S13-GPI-HF (43.04220 Da)
Charge: +2, Monoisotopic m/z: 736.78503 Da (-0.04 mmu/-0.05 ppm), MH+: 1472.56279 Da, RT: 11.98 min,
Identified with: Mascot (v1.30); IonScore:75, Exp Value:4.3E-007, Matched Ions: 11/114
Fragment Match Tolerance: 0.5 Da
Protein References (1):
- Netrin-G1 OS=Homo sapiens GN=NTNG1 PE=1 SV=3 - [NTNG1_HUMAN]

## Slide 60
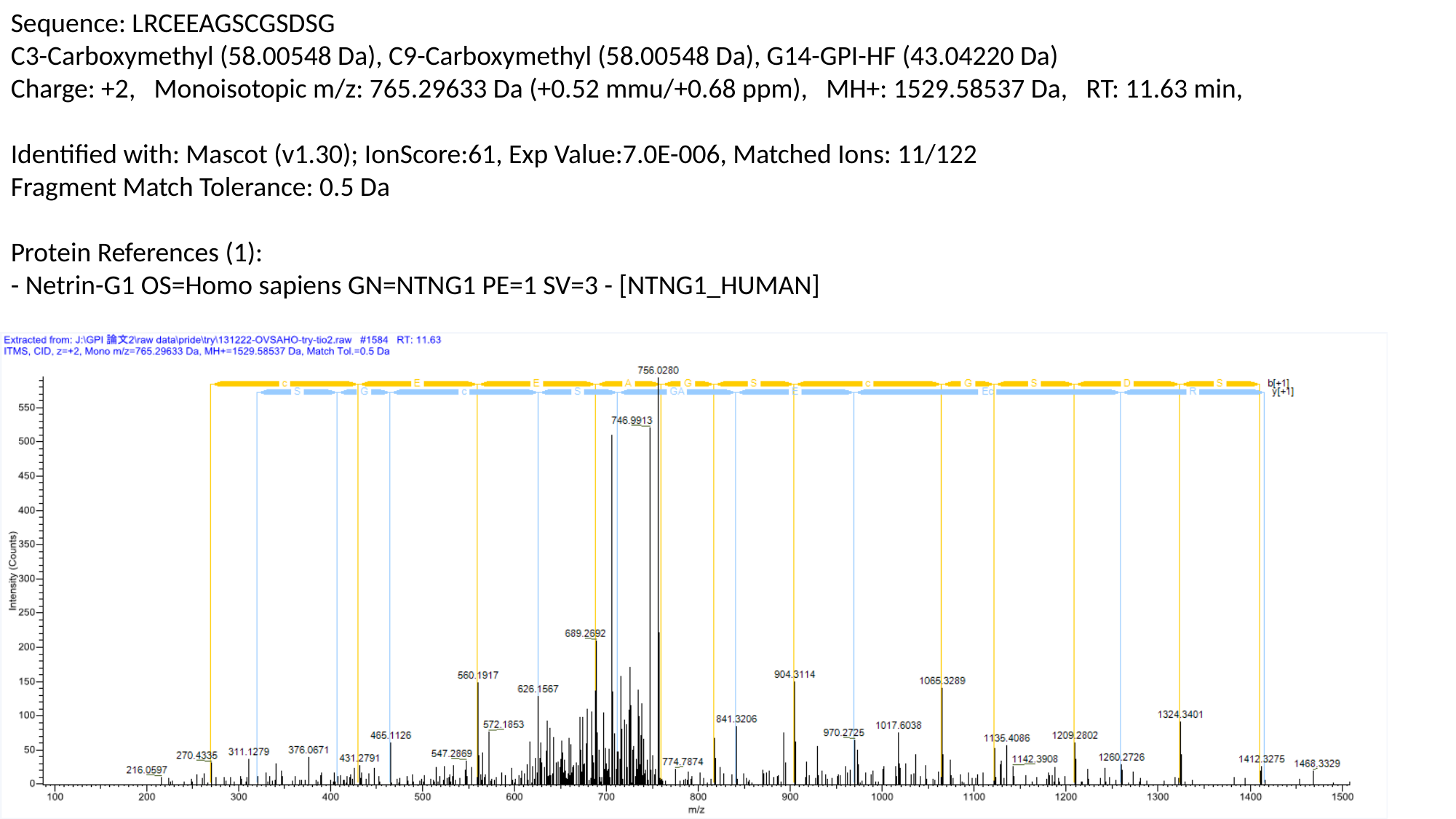

Sequence: LRCEEAGSCGSDSG
C3-Carboxymethyl (58.00548 Da), C9-Carboxymethyl (58.00548 Da), G14-GPI-HF (43.04220 Da)
Charge: +2, Monoisotopic m/z: 765.29633 Da (+0.52 mmu/+0.68 ppm), MH+: 1529.58537 Da, RT: 11.63 min,
Identified with: Mascot (v1.30); IonScore:61, Exp Value:7.0E-006, Matched Ions: 11/122
Fragment Match Tolerance: 0.5 Da
Protein References (1):
- Netrin-G1 OS=Homo sapiens GN=NTNG1 PE=1 SV=3 - [NTNG1_HUMAN]

## Slide 61
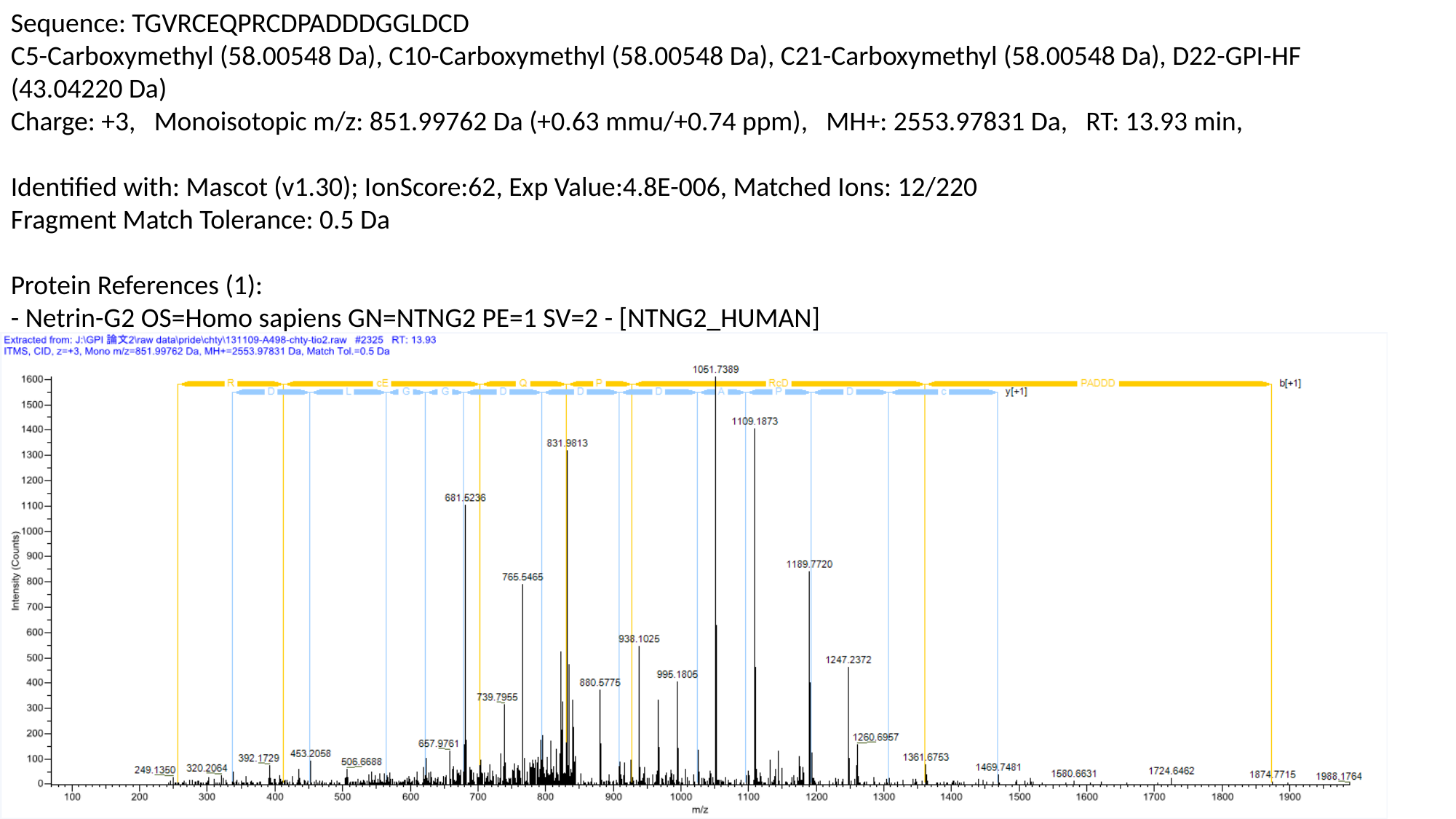

Sequence: TGVRCEQPRCDPADDDGGLDCD
C5-Carboxymethyl (58.00548 Da), C10-Carboxymethyl (58.00548 Da), C21-Carboxymethyl (58.00548 Da), D22-GPI-HF (43.04220 Da)
Charge: +3, Monoisotopic m/z: 851.99762 Da (+0.63 mmu/+0.74 ppm), MH+: 2553.97831 Da, RT: 13.93 min,
Identified with: Mascot (v1.30); IonScore:62, Exp Value:4.8E-006, Matched Ions: 12/220
Fragment Match Tolerance: 0.5 Da
Protein References (1):
- Netrin-G2 OS=Homo sapiens GN=NTNG2 PE=1 SV=2 - [NTNG2_HUMAN]

## Slide 62
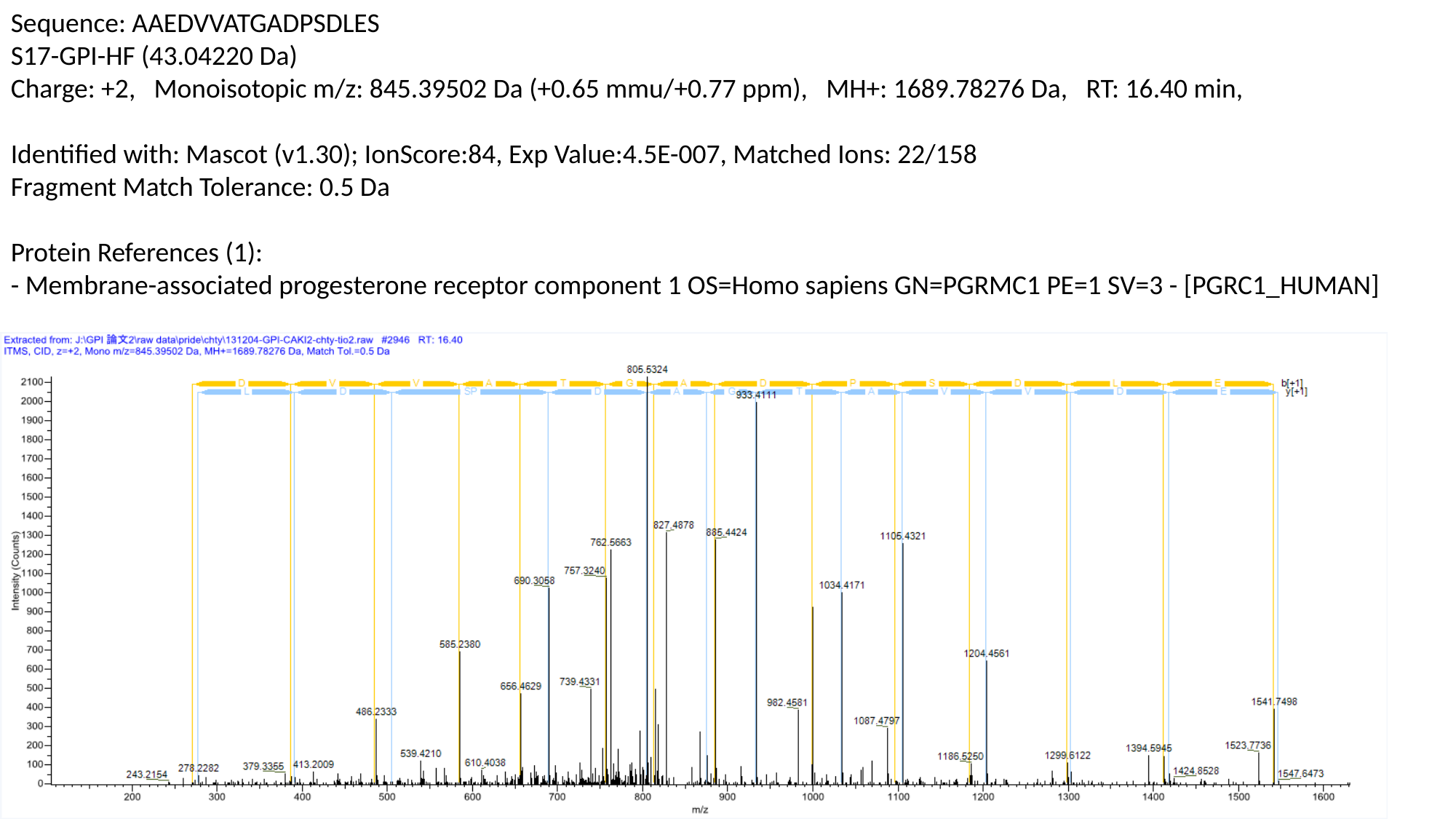

Sequence: AAEDVVATGADPSDLES
S17-GPI-HF (43.04220 Da)
Charge: +2, Monoisotopic m/z: 845.39502 Da (+0.65 mmu/+0.77 ppm), MH+: 1689.78276 Da, RT: 16.40 min,
Identified with: Mascot (v1.30); IonScore:84, Exp Value:4.5E-007, Matched Ions: 22/158
Fragment Match Tolerance: 0.5 Da
Protein References (1):
- Membrane-associated progesterone receptor component 1 OS=Homo sapiens GN=PGRMC1 PE=1 SV=3 - [PGRC1_HUMAN]

## Slide 63
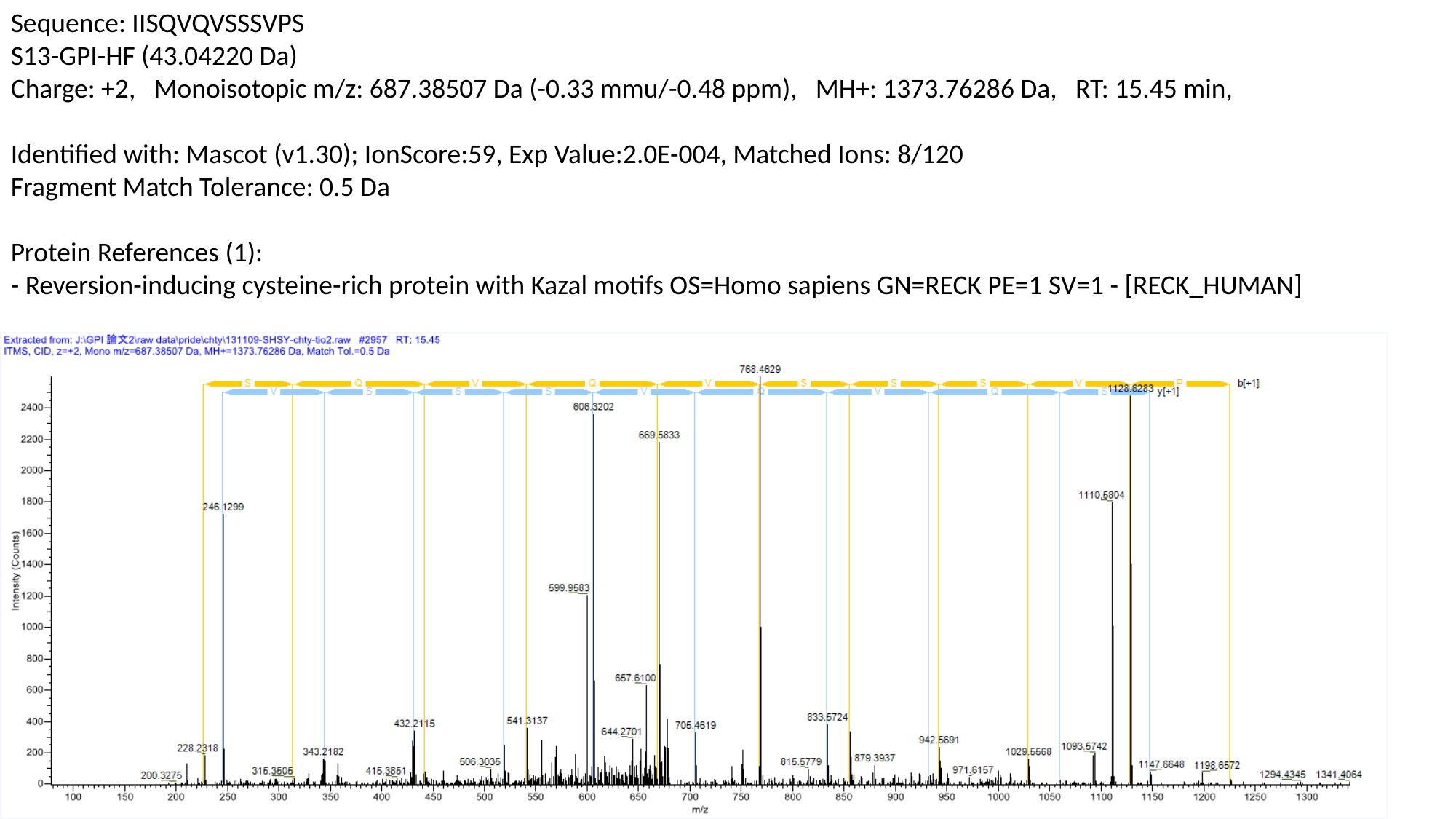

Sequence: IISQVQVSSSVPS
S13-GPI-HF (43.04220 Da)
Charge: +2, Monoisotopic m/z: 687.38507 Da (-0.33 mmu/-0.48 ppm), MH+: 1373.76286 Da, RT: 15.45 min,
Identified with: Mascot (v1.30); IonScore:59, Exp Value:2.0E-004, Matched Ions: 8/120
Fragment Match Tolerance: 0.5 Da
Protein References (1):
- Reversion-inducing cysteine-rich protein with Kazal motifs OS=Homo sapiens GN=RECK PE=1 SV=1 - [RECK_HUMAN]

## Slide 64
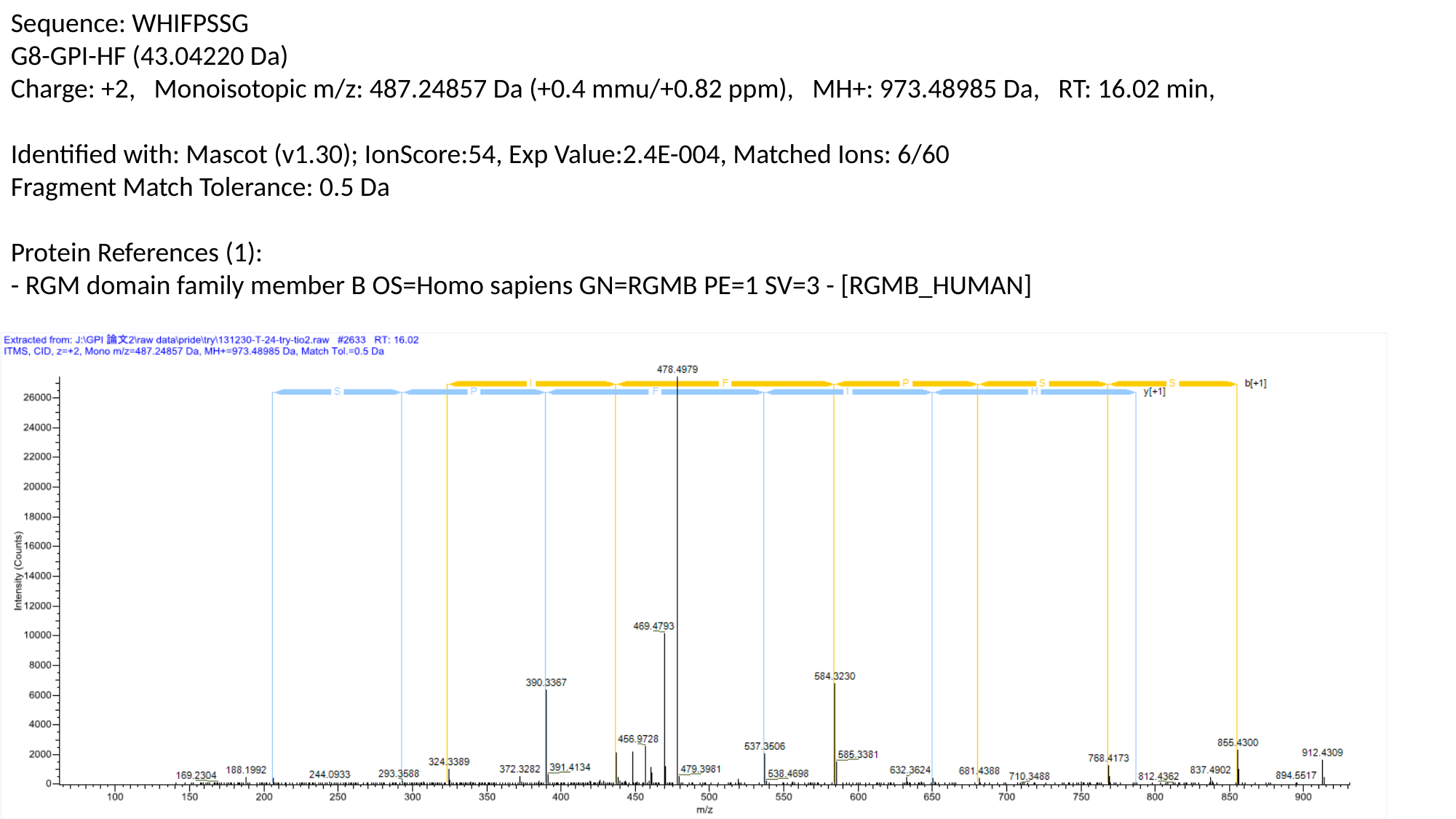

Sequence: WHIFPSSG
G8-GPI-HF (43.04220 Da)
Charge: +2, Monoisotopic m/z: 487.24857 Da (+0.4 mmu/+0.82 ppm), MH+: 973.48985 Da, RT: 16.02 min,
Identified with: Mascot (v1.30); IonScore:54, Exp Value:2.4E-004, Matched Ions: 6/60
Fragment Match Tolerance: 0.5 Da
Protein References (1):
- RGM domain family member B OS=Homo sapiens GN=RGMB PE=1 SV=3 - [RGMB_HUMAN]

## Slide 65
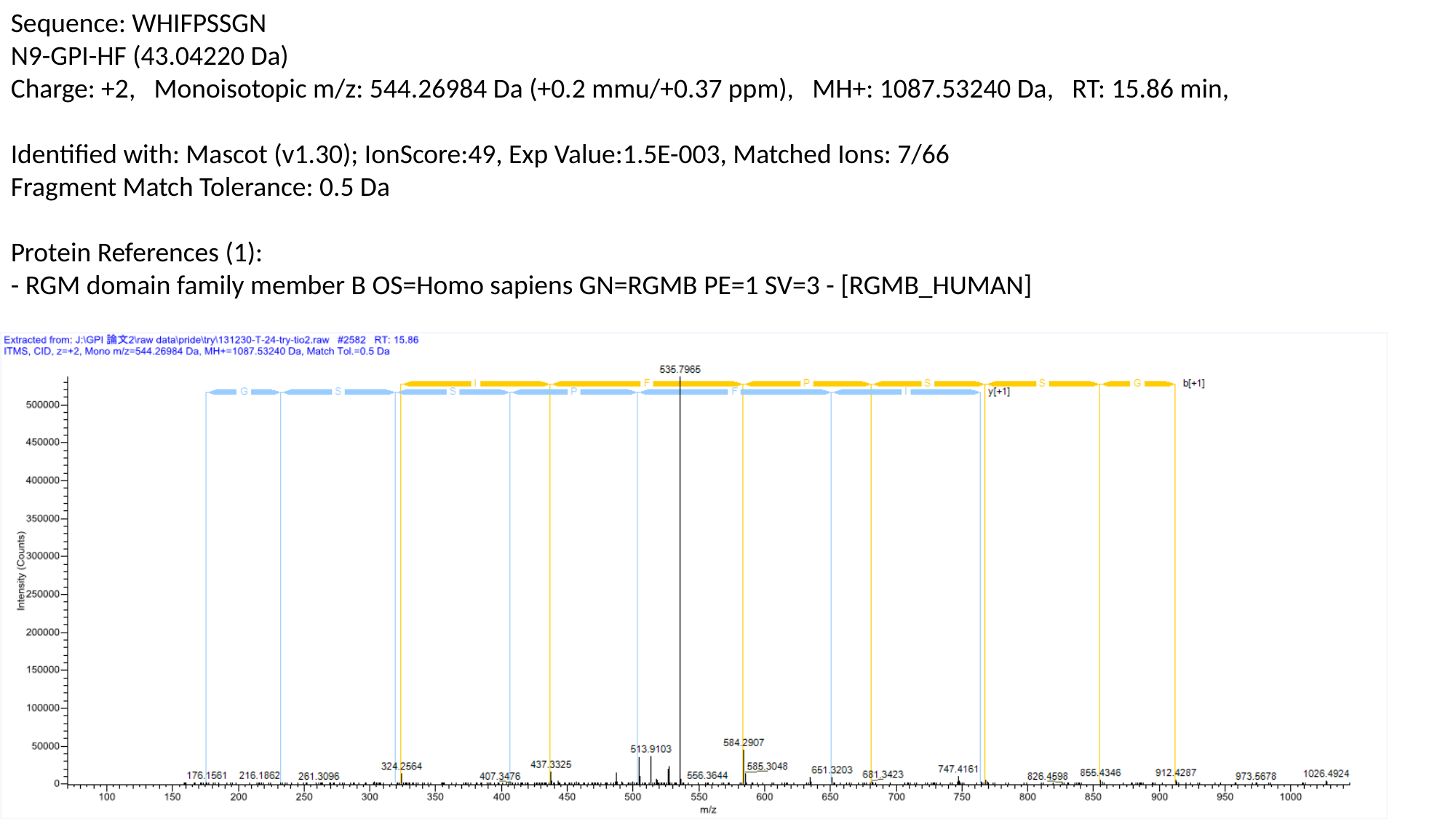

Sequence: WHIFPSSGN
N9-GPI-HF (43.04220 Da)
Charge: +2, Monoisotopic m/z: 544.26984 Da (+0.2 mmu/+0.37 ppm), MH+: 1087.53240 Da, RT: 15.86 min,
Identified with: Mascot (v1.30); IonScore:49, Exp Value:1.5E-003, Matched Ions: 7/66
Fragment Match Tolerance: 0.5 Da
Protein References (1):
- RGM domain family member B OS=Homo sapiens GN=RGMB PE=1 SV=3 - [RGMB_HUMAN]

## Slide 66
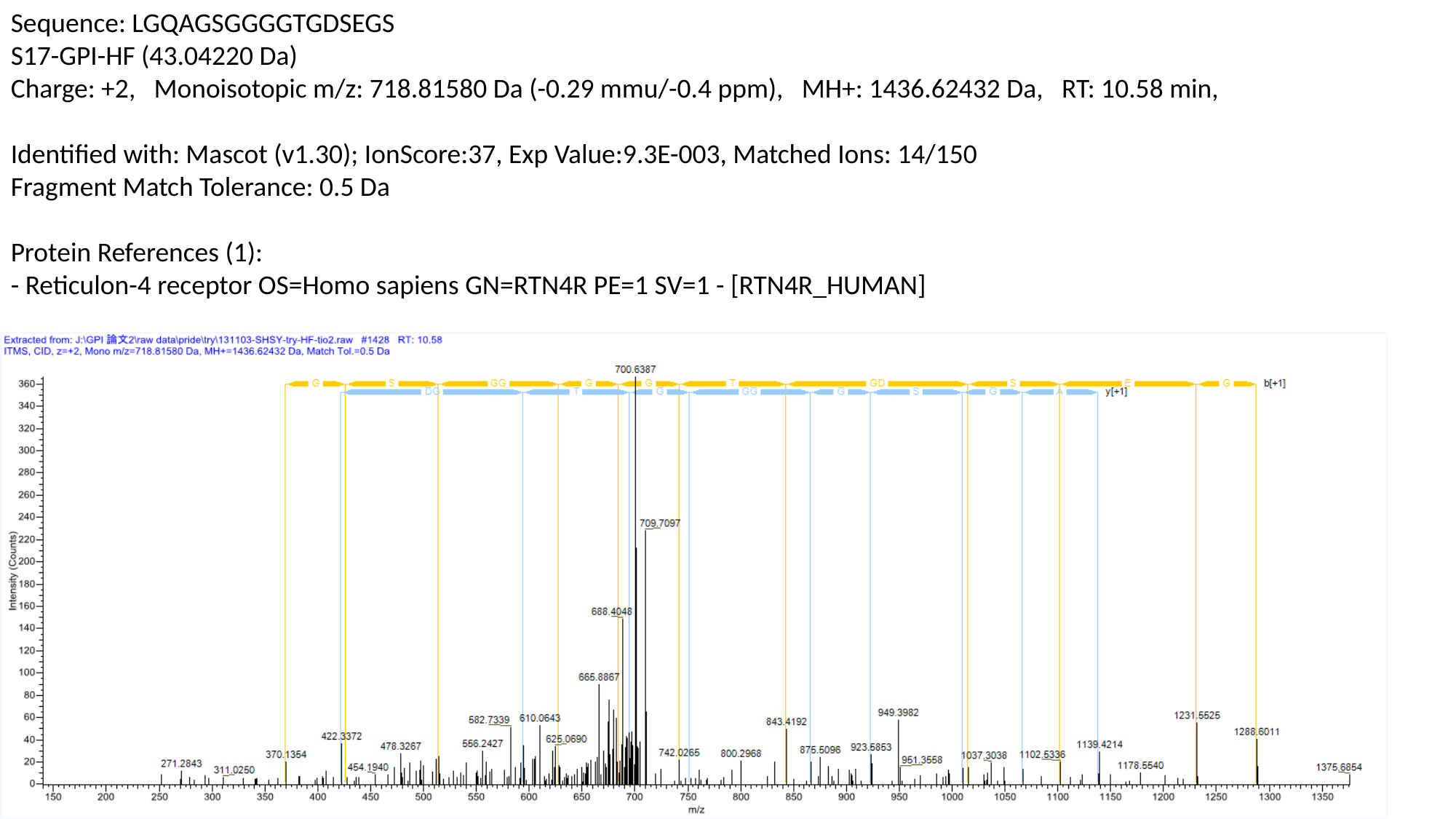

Sequence: LGQAGSGGGGTGDSEGS
S17-GPI-HF (43.04220 Da)
Charge: +2, Monoisotopic m/z: 718.81580 Da (-0.29 mmu/-0.4 ppm), MH+: 1436.62432 Da, RT: 10.58 min,
Identified with: Mascot (v1.30); IonScore:37, Exp Value:9.3E-003, Matched Ions: 14/150
Fragment Match Tolerance: 0.5 Da
Protein References (1):
- Reticulon-4 receptor OS=Homo sapiens GN=RTN4R PE=1 SV=1 - [RTN4R_HUMAN]

## Slide 67
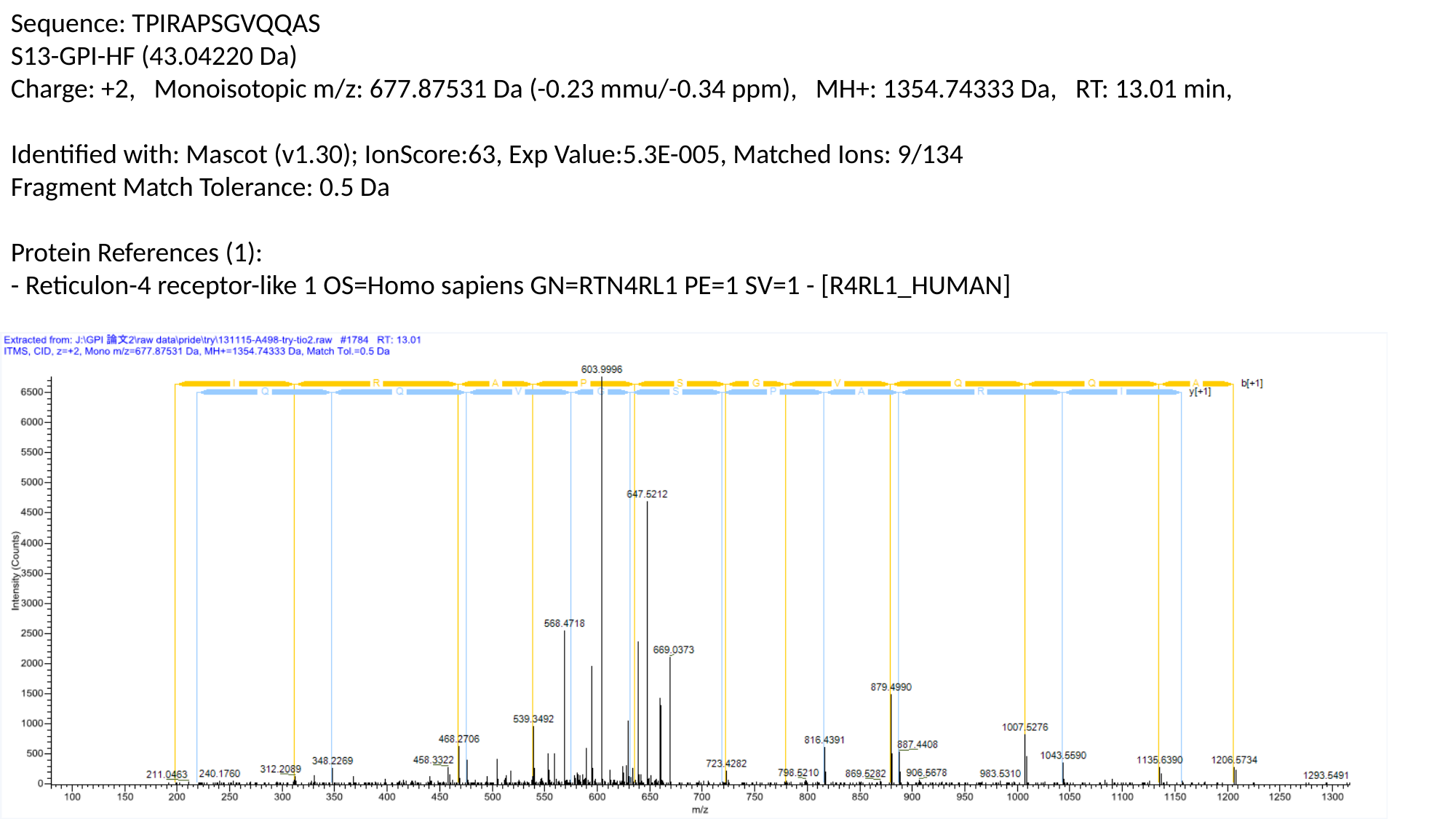

Sequence: TPIRAPSGVQQAS
S13-GPI-HF (43.04220 Da)
Charge: +2, Monoisotopic m/z: 677.87531 Da (-0.23 mmu/-0.34 ppm), MH+: 1354.74333 Da, RT: 13.01 min,
Identified with: Mascot (v1.30); IonScore:63, Exp Value:5.3E-005, Matched Ions: 9/134
Fragment Match Tolerance: 0.5 Da
Protein References (1):
- Reticulon-4 receptor-like 1 OS=Homo sapiens GN=RTN4RL1 PE=1 SV=1 - [R4RL1_HUMAN]

## Slide 68
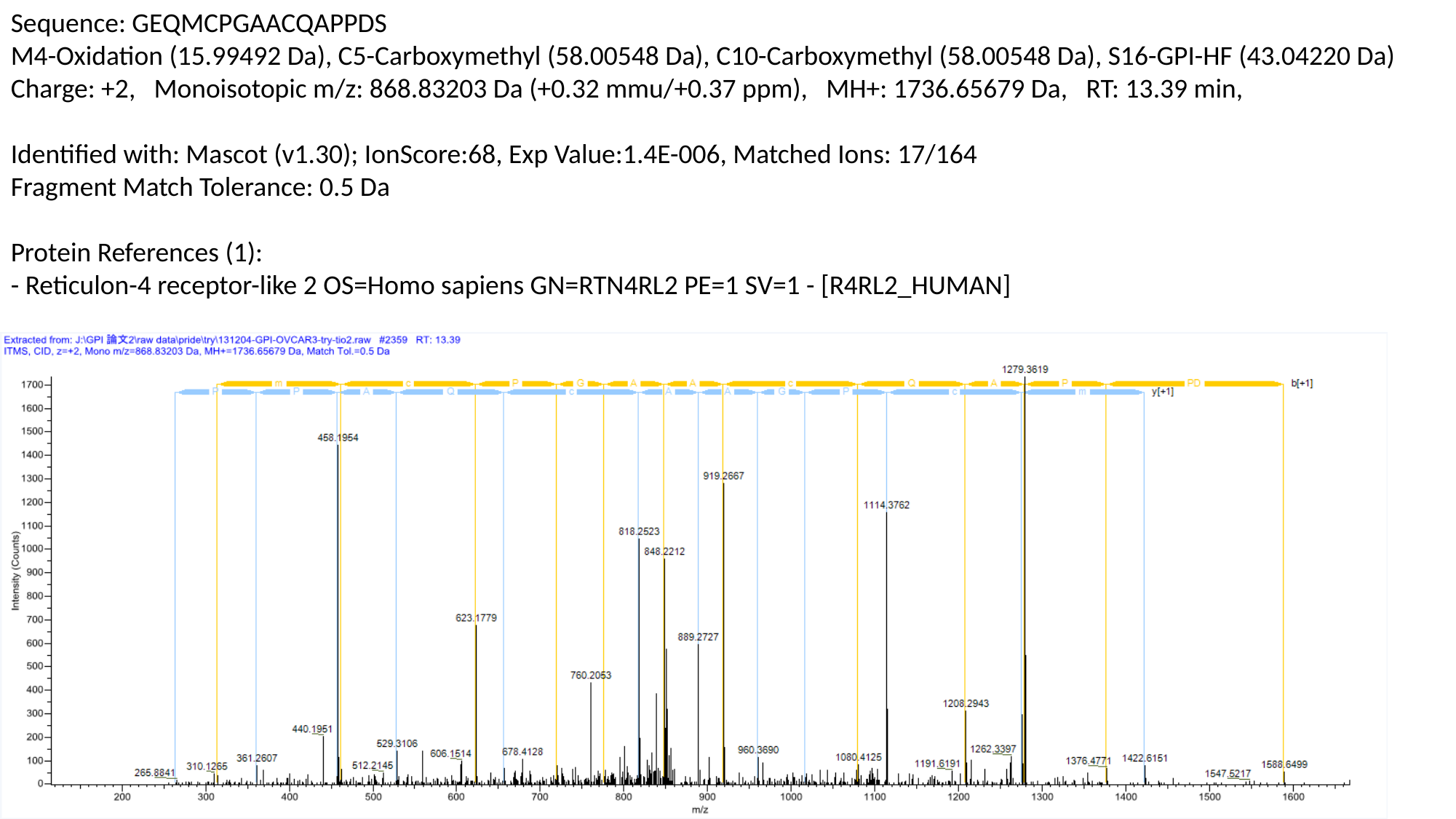

Sequence: GEQMCPGAACQAPPDS
M4-Oxidation (15.99492 Da), C5-Carboxymethyl (58.00548 Da), C10-Carboxymethyl (58.00548 Da), S16-GPI-HF (43.04220 Da)
Charge: +2, Monoisotopic m/z: 868.83203 Da (+0.32 mmu/+0.37 ppm), MH+: 1736.65679 Da, RT: 13.39 min,
Identified with: Mascot (v1.30); IonScore:68, Exp Value:1.4E-006, Matched Ions: 17/164
Fragment Match Tolerance: 0.5 Da
Protein References (1):
- Reticulon-4 receptor-like 2 OS=Homo sapiens GN=RTN4RL2 PE=1 SV=1 - [R4RL2_HUMAN]

## Slide 69
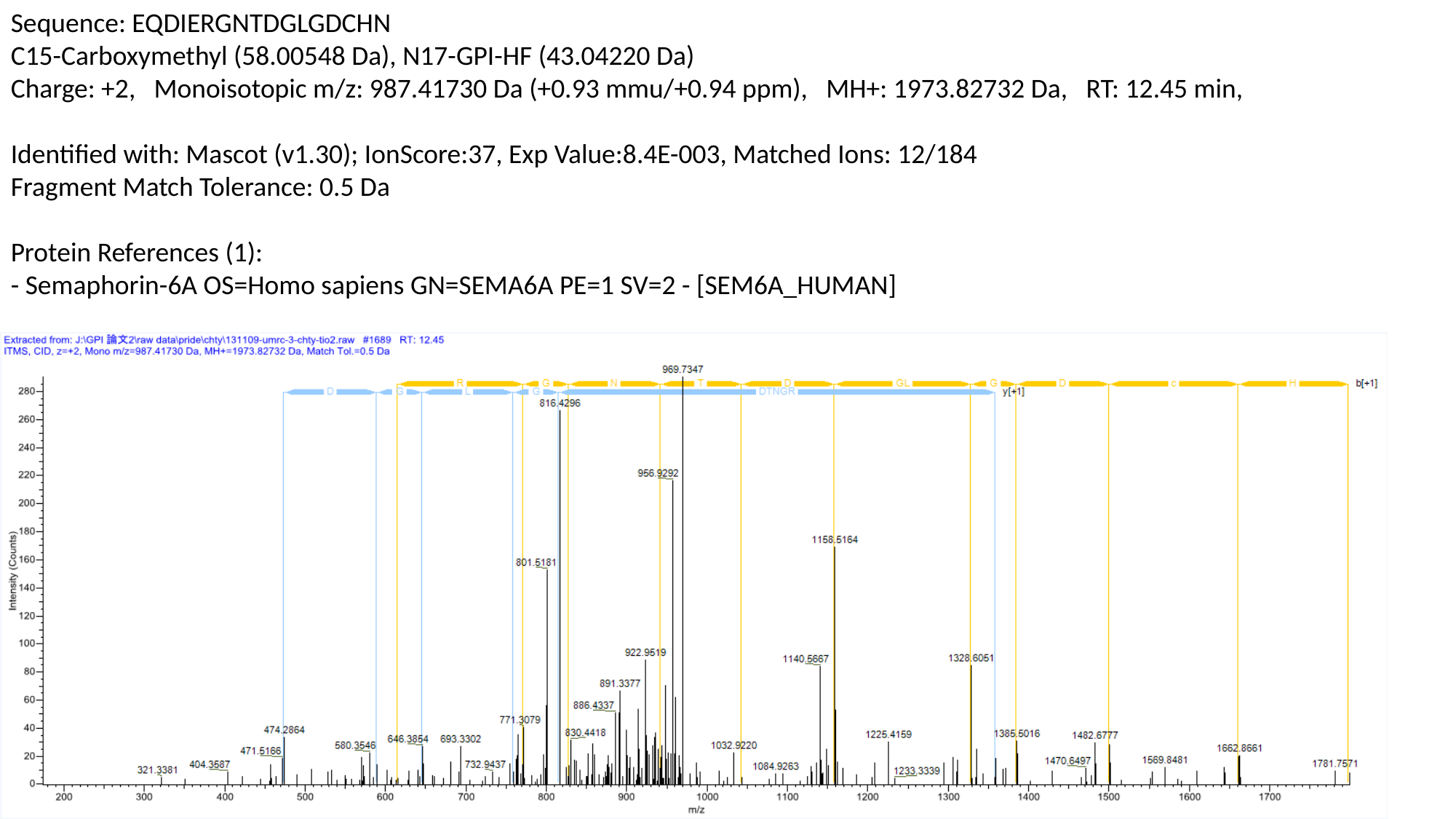

Sequence: EQDIERGNTDGLGDCHN
C15-Carboxymethyl (58.00548 Da), N17-GPI-HF (43.04220 Da)
Charge: +2, Monoisotopic m/z: 987.41730 Da (+0.93 mmu/+0.94 ppm), MH+: 1973.82732 Da, RT: 12.45 min,
Identified with: Mascot (v1.30); IonScore:37, Exp Value:8.4E-003, Matched Ions: 12/184
Fragment Match Tolerance: 0.5 Da
Protein References (1):
- Semaphorin-6A OS=Homo sapiens GN=SEMA6A PE=1 SV=2 - [SEM6A_HUMAN]

## Slide 70
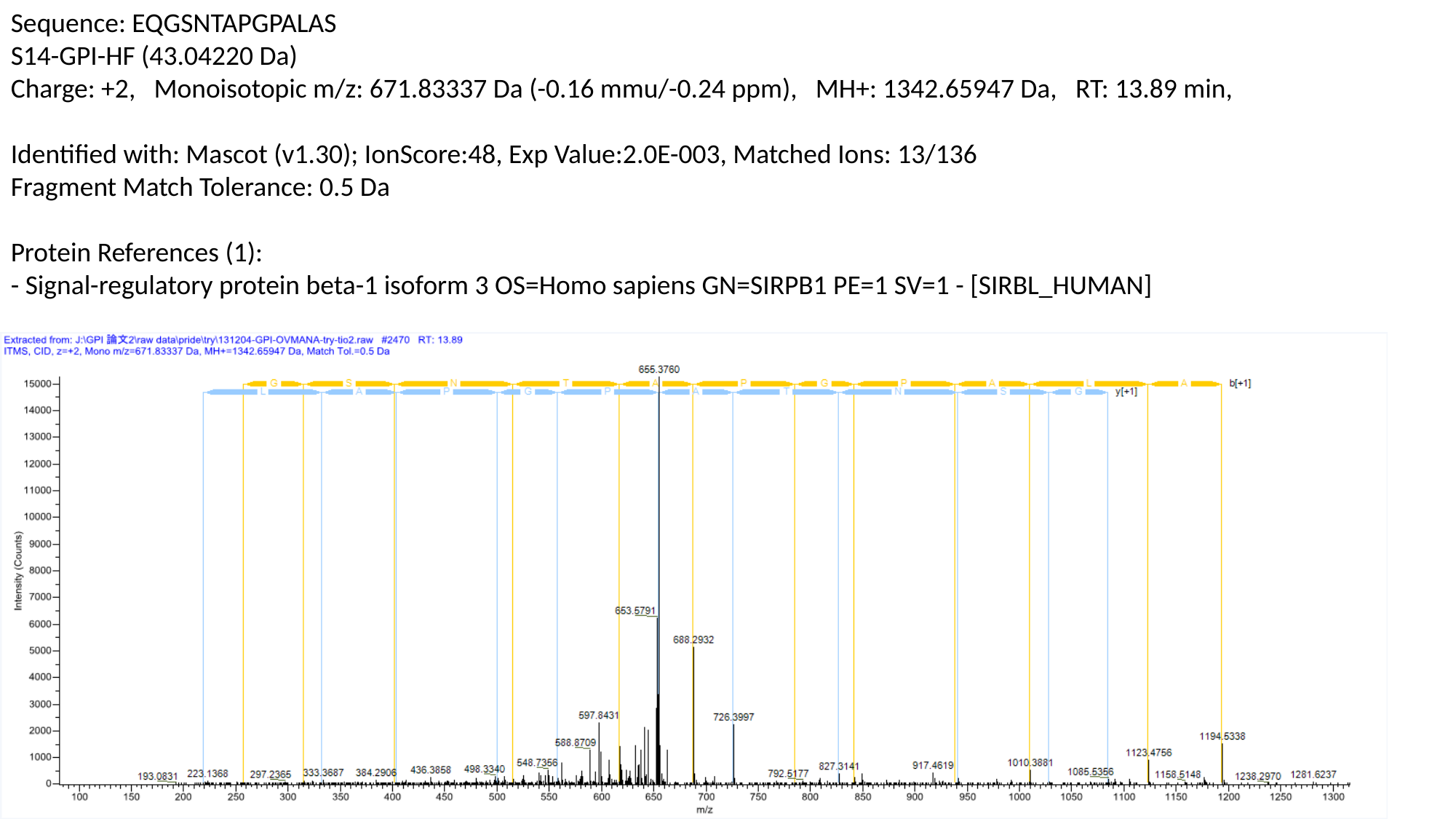

Sequence: EQGSNTAPGPALAS
S14-GPI-HF (43.04220 Da)
Charge: +2, Monoisotopic m/z: 671.83337 Da (-0.16 mmu/-0.24 ppm), MH+: 1342.65947 Da, RT: 13.89 min,
Identified with: Mascot (v1.30); IonScore:48, Exp Value:2.0E-003, Matched Ions: 13/136
Fragment Match Tolerance: 0.5 Da
Protein References (1):
- Signal-regulatory protein beta-1 isoform 3 OS=Homo sapiens GN=SIRPB1 PE=1 SV=1 - [SIRBL_HUMAN]

## Slide 71
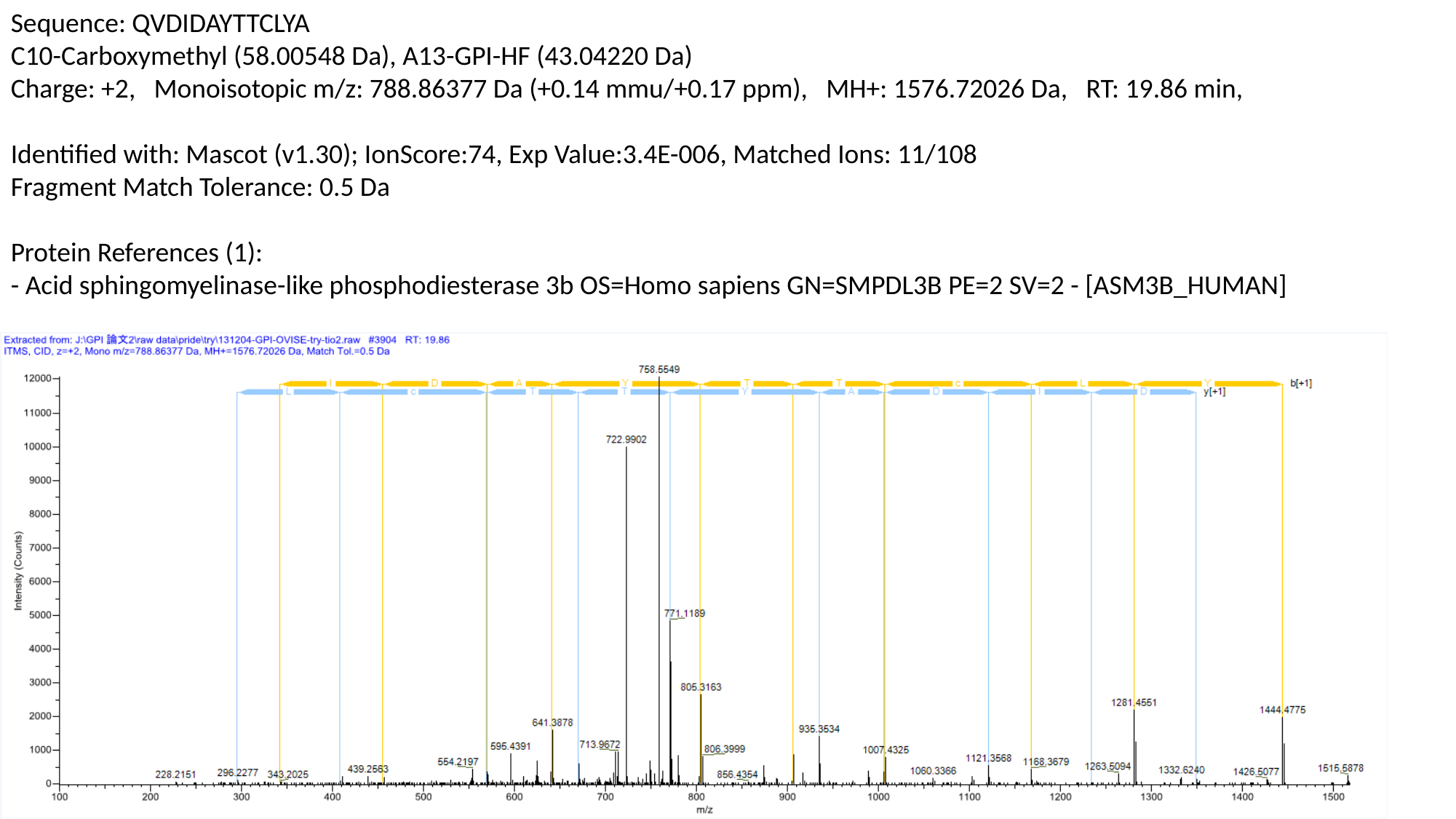

Sequence: QVDIDAYTTCLYA
C10-Carboxymethyl (58.00548 Da), A13-GPI-HF (43.04220 Da)
Charge: +2, Monoisotopic m/z: 788.86377 Da (+0.14 mmu/+0.17 ppm), MH+: 1576.72026 Da, RT: 19.86 min,
Identified with: Mascot (v1.30); IonScore:74, Exp Value:3.4E-006, Matched Ions: 11/108
Fragment Match Tolerance: 0.5 Da
Protein References (1):
- Acid sphingomyelinase-like phosphodiesterase 3b OS=Homo sapiens GN=SMPDL3B PE=2 SV=2 - [ASM3B_HUMAN]

## Slide 72
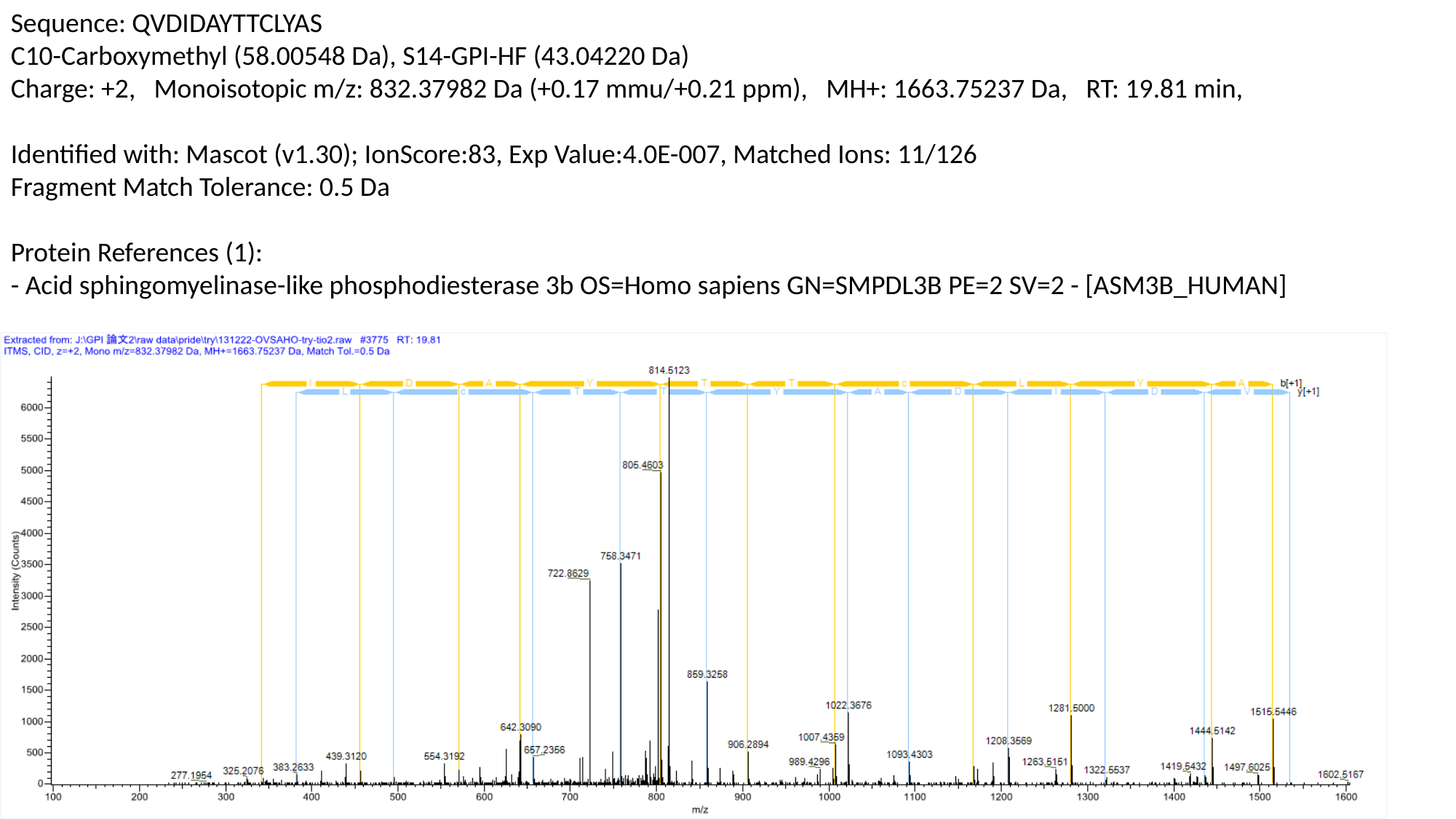

Sequence: QVDIDAYTTCLYAS
C10-Carboxymethyl (58.00548 Da), S14-GPI-HF (43.04220 Da)
Charge: +2, Monoisotopic m/z: 832.37982 Da (+0.17 mmu/+0.21 ppm), MH+: 1663.75237 Da, RT: 19.81 min,
Identified with: Mascot (v1.30); IonScore:83, Exp Value:4.0E-007, Matched Ions: 11/126
Fragment Match Tolerance: 0.5 Da
Protein References (1):
- Acid sphingomyelinase-like phosphodiesterase 3b OS=Homo sapiens GN=SMPDL3B PE=2 SV=2 - [ASM3B_HUMAN]

## Slide 73
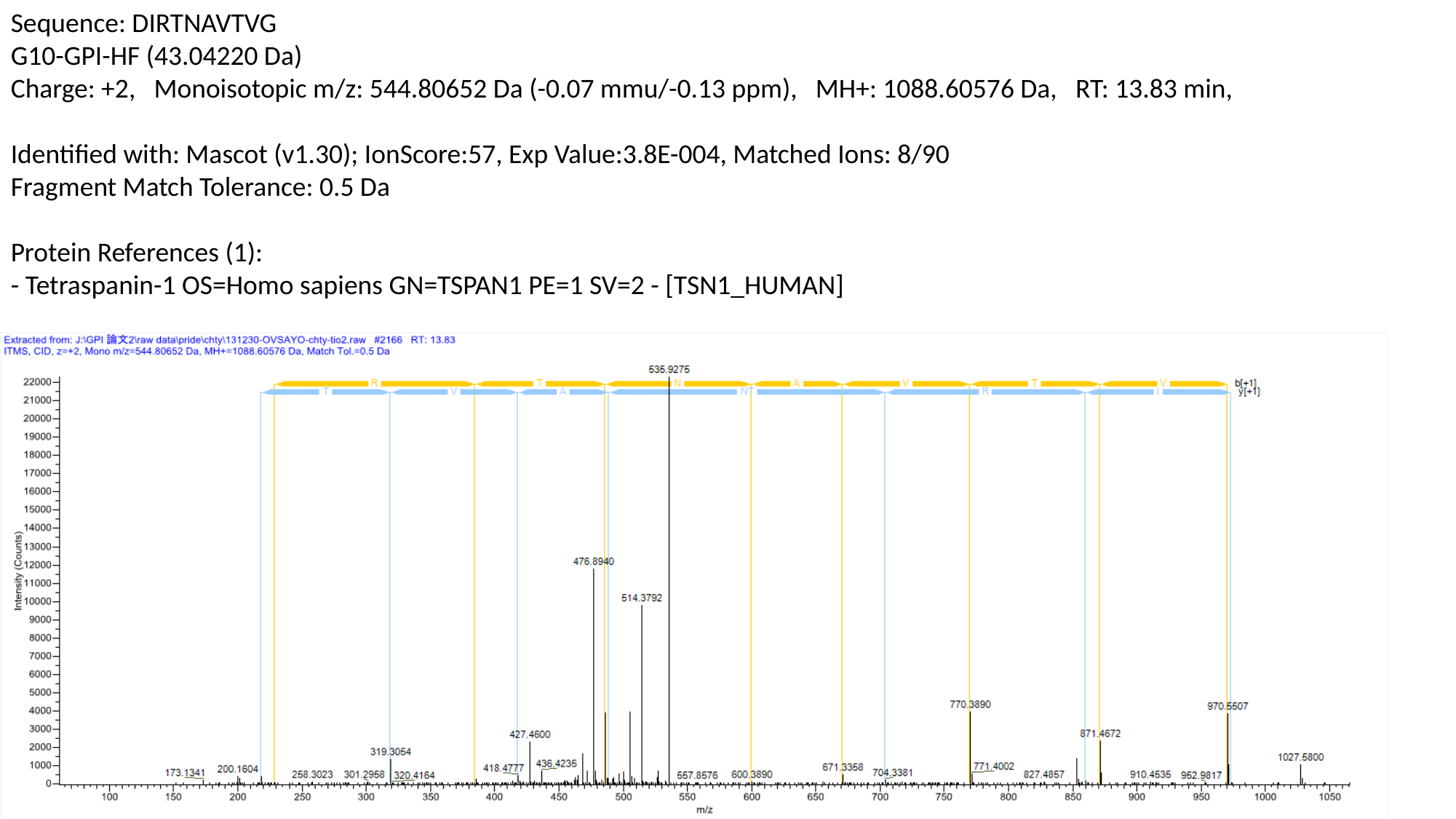

Sequence: DIRTNAVTVG
G10-GPI-HF (43.04220 Da)
Charge: +2, Monoisotopic m/z: 544.80652 Da (-0.07 mmu/-0.13 ppm), MH+: 1088.60576 Da, RT: 13.83 min,
Identified with: Mascot (v1.30); IonScore:57, Exp Value:3.8E-004, Matched Ions: 8/90
Fragment Match Tolerance: 0.5 Da
Protein References (1):
- Tetraspanin-1 OS=Homo sapiens GN=TSPAN1 PE=1 SV=2 - [TSN1_HUMAN]
